# The histone variant H2A.W restricts heterochromatic crossovers in *Arabidopsis*

**DOI:** 10.1073/pnas.2413698122

**Published:** 2025-04-04

**Authors:** Namil Son, Heejin Kim, Jaeil Kim, Jihye Park, Dohwan Byun, Sang-jun Park, Hyein Kim, Yeong Mi Park, Pierre Bourguet, Frédéric Berger, Kyuha Choi

**Affiliations:** ^a^Department of Life Sciences, Pohang University of Science and Technology, Pohang 37673, Republic of Korea; ^b^Gregor Mendel Institute, Austrian Academy of Sciences, Vienna BioCenter, Vienna 1030, Austria

**Keywords:** *Arabidopsis thaliana*, H2A.W, heterochromatin, meiotic crossover

## Abstract

Meiotic crossovers rearrange allele combinations and create offspring diversity. Crossovers occur nonrandomly along chromosomes, predominantly in distal euchromatin and less in pericentromeric heterochromatin marked with histone H3 lysine 9 dimethylation (H3K9me2) and the H2A variant H2A.W in *Arabidopsis thaliana*. Loss of H3K9me2 increases heterochromatic crossovers, but how H2A.W affects crossover formation in pericentromeric regions is unknown. Here, we report that H2A.W is required to restrict heterochromatic crossovers in *Arabidopsis*. Using meiosis-specific microRNA-induced gene silencing (meiMIGS) and fluorescence-tagged recombination reporters, we show that meiotic knockdown of *H2A.W.6*, *H2A.W.7*, and *H2A.W.12* (*meiMIGS-H2A.W.6/7/12*) increases pericentromeric crossovers. High-resolution genomic maps of crossovers show that *meiMIGS-H2A.W.6/7/12* enhances heterochromatic crossovers, similar to *meiMIGS* plants silencing the H3K9me2 pathway. Consistently, genome-wide crossover maps show that the mutants *h2a.w.6*, *h2a.w.7*, *h2a.w.6 h2a.w.7*, and *h2a.w.6 h2a.w.7 h2a.w.12,* but not *h2a.w.12,* exhibit a similar increase in heterochromatic crossovers to *meiMIGS-H2A.W.6/7/12*, demonstrating that H2A.W.6 and H2A.W.7 limit heterochromatic crossovers. Profiling of genome-wide nucleosome density using micrococcal nuclease sequencing reveals that *h2a.w* mutants with increased heterochromatic crossovers have increased heterochromatin accessibility, with lower H3K9me2 levels during meiosis. Our findings shed light on the role of H2A.W variants as heterochromatin compaction factors that suppress meiotic crossovers within the pericentromeric regions.

## Significance

Meiotic crossovers are crucial for generating genetic diversity in progeny by reshuffling existing variation present in the two parents. However, crossovers rarely occur within heterochromatic pericentromeres, and the molecular mechanisms underlying their suppression in these regions remain largely unexplored. Here, we demonstrate that the heterochromatic histone H2A variant H2A.W is required to limit pericentromeric crossovers in *Arabidopsis thaliana*. Meiosis-specific silencing of three *H2A.W* genes and their genetic loss are associated with increased heterochromatic crossovers, possibly through reduced heterochromatin compaction and H3K9me2 levels, which may facilitate the recombination process during meiosis. This study thus provides valuable insights into unlocking heterochromatic crossovers through the regulation of H2A.W.

During meiosis, homologous chromosomes reciprocally exchange genetic material through crossover events, creating a greater genetic diversity in the progeny (1). Meiotic recombination is initiated with the formation of DNA double-strand breaks (DSBs) by SPO11 and its associated proteins (2, 3). In plants, the number of meiotic DSBs that occur far exceeds the final number of crossovers, as only about 5% of these DSBs are repaired using nonsister chromatids as templates, eventually forming 1 to 3 crossovers per chromosome pair (2). The remaining DSBs are repaired by sister chromatids or processed into non-crossovers. The ends of meiotic DSBs are processed to produce single-strand DNAs that then undergo strand invasion into sister or nonsister chromatids by homology search, generating displacement loop structures (4, 5). A subset of these are repaired as crossovers through two pathways: class I and class II (2, 6). Class I crossovers are mediated by a group of pro-crossover proteins, including the E3 ligase HEI10 (2, 7, 8). The class I pathway is responsible for most crossovers (~85 to 90%) in *Arabidopsis thaliana* (*A. thaliana*) (9–12), which exhibit interference. In crossover interference, one crossover inhibits the formation of another closely spaced crossover, resulting in multiple crossovers being more evenly distributed along chromosomes than a random distribution (2, 13). The number and positions of interfering crossovers can be explained by the dosage and coarsening dynamics of HEI10 proteins in *A. thaliana* (2, 13). The class II crossover pathway mediates the remaining minority of crossovers (~10 to 15%) via the endonuclease MUS81 (14). Class II crossovers are noninterfering and restricted by multiple anti-crossover factors (15–17).

Genetic inactivation of the anti-crossover factors, such as the RecQ helicases RECQ4A and RECQ4B in the class II pathway, elevates the number of noninterfering crossovers in *A. thaliana* (18, 19). Overexpression of the dosage-dependent *HEI10* and genetic mutations of anti-crossover genes *HIGH CROSSOVER RATE1* (*HCR1*), *HCR2*, *HCR3*, or of genes encoding components of the synaptonemal complex increase class I crossovers in *A. thaliana* (20–28). This increase in crossover number by genetic manipulation of the class I and class II pathways occurs predominantly along euchromatic chromosomal arms, while crossover formation remains suppressed at heterochromatic pericentromeres and centromeres (19, 23, 29). However, molecular mechanisms by which heterochromatic crossovers are suppressed remain largely unclear (30, 31).

In *A. thaliana*, meiotic DSBs and crossovers correlate with chromatin accessibility and gene density (30, 32–35), being strongly suppressed in transposon-rich pericentromeric regions (29, 33). Plant constitutive heterochromatic nucleosomes in the pericentromeric regions are characterized by methylation of DNA wrapped around them, the posttranslational methylation of histone H3 at lysine 9 (H3K9me1 and H3K9me2) and lysine 27 (H3K27me1), some degree of enrichment of the linker histone H1, and the specific association of the histone H2A variant H2A.W (36–42). In *A. thaliana*, mutations inactivating the histone methyltransferases, SU(VAR)3-9 HOMOLOG 4 (SUVH4), SUVH5, and SUVH6, which catalyze H3K9me2 increase pericentromeric crossovers (30). Consistent with a self-reinforcing loop between H3K9me2 and non-CG methylation (42, 43), loss of the DNA methyltransferase CHROMOMETHYLASE 3 (CMT3), catalyzing non-CG methylation in the CHG context, leads to more heterochromatic crossovers (30, 44). However, mutations of *DNA METHYLTRANSFERASE 1* (*MET1*), required for maintaining CG DNA methylation at heterochromatin, or of *DECREASE IN DNA METHYLATION 1* (*DDM1*), required for both CG and non-CG methylation, are associated with fewer pericentromeric crossovers and more crossovers along chromosome arms related to remodeling the crossover landscape (35, 45, 46), suggesting a complex relationship between heterochromatin-organizing factors and crossover formation.

*A. thaliana* has three genes, *H2A.W.6*, *H2A.W.7*, and *H2A.W.12*, that encode the histone H2A variant H2A.W isoforms required for heterochromatin compaction (37, 38, 47). H2A.W plays a cooperative role with linker histone H1, non-CG DNA methylation, and H3K9me2 in heterochromatin condensation and transcriptional silencing of transposons (48, 49). However, whether H2A.Ws limit pericentromeric crossovers remains unexplored. To investigate the role of H2A.W in the frequency and distribution of crossovers in *A. thaliana* heterochromatic and pericentromeric regions—which occupy about 27% of the genome and contain approximately 70.8% of transposons marked densely with DNA methylation, H3K9me2, H3K27me1, and histone H1—we use here the meiosis-specific microRNA-induced gene silencing (meiMIGS) system, which allows multiple gene knockdown and high-throughput measurements of crossover frequency (24, 50), as well as the *h2a.w* mutant alleles newly generated by genome editing, genomic mapping of crossovers and nucleosome occupancy, and immunocytology. We demonstrate that meiotic knockdown or loss of H2A.W leads to more crossovers in heterochromatic regions, with increased heterochromatin accessibility and decreased meiotic H3K9me2 levels. Therefore, our findings reveal an additional layer for epigenetic reactivation of heterochromatic crossovers via control of H2A.Ws in plants.

## Results

### Meiotic Silencing of Three *H2A.W* Genes Increases Heterochromatic Crossovers.

**Fig. 1.**
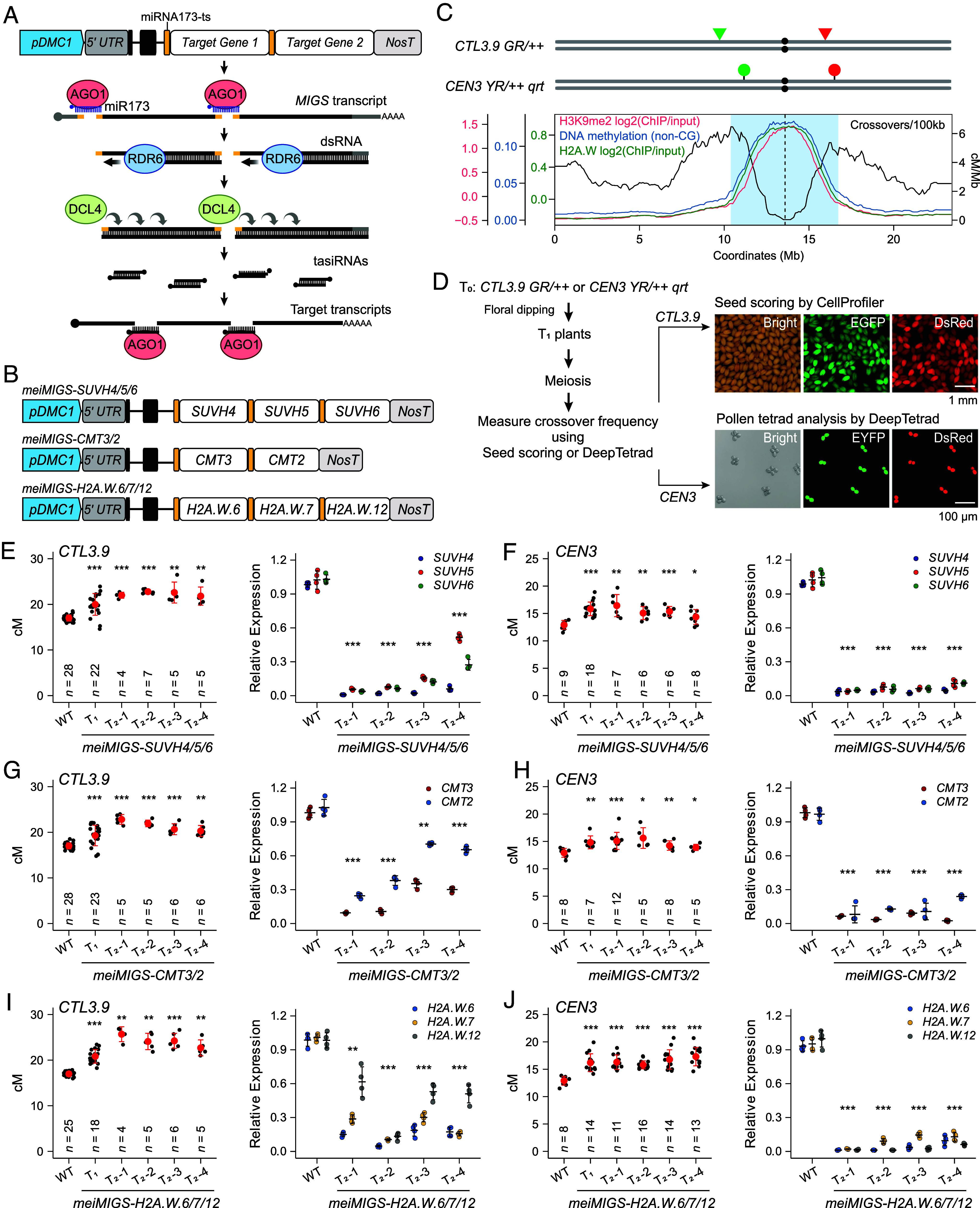
Meiotic knockdown of *H2A.W* increases crossover frequencies in pericentromeric *CTL3.9* and *CEN3* FTLs. (*A*) Diagram of the meiosis-specific miR173-induced gene silencing (meiMIGS) construct targeting multiple genes. miR173-ts, 22-nt miR173 target sequence. (*B*) Plasmids used for meiMIGS of *H2A.W6/7/12, SUVH4/5/6*, and *CMT3/2*. (*C*) Landscapes of crossovers, H2A.W density, H3K9me2, DNA methylation, and T-DNA insertions in the *CTL3.9* and *CEN3* FTLs for chromosome 3. Triangles and circles indicate *NapA-* and *LAT52*-driven FTL transgenes, respectively. The centromere gap is marked with a vertical dashed line and the pericentromeric region is shaded blue. (*D*) Diagram of meiMIGS application to seed (*CTL3.9*) and pollen tetrad (*CEN3*) FTLs for high-throughput measurements of crossover frequency. (*E* and *F*) *CTL3.9* (*E*) and *CEN3* (*F*) crossover frequencies (cM) and RT-qPCR analysis of relative *SUVH4, SUVH5*, and *SUVH6* transcript levels to *DMC1* in *meiMIGS-SUVH4/5/6* lines. (*G* and *H*) As for (*E* and *F*), but showing *meiMIGS-CMT3/2*. (*I* and *J*) As for (*E* and *F*), but showing *meiMIGS-H2A.W.6/7/12*. In (*E*–*J*, *Left*), data are means ± SD of cM values from individual plants (*n* = number of plants). In (*E*–*J*, *Right*), data are means ± SD of *n* = 4 biological replicates. For (*E*–*J*), asterisks indicate significant differences (*P < 0.05, **P < 0.01, ***P < 0.001; two-sided Welch’s *t* test). Datasets and *P-*values are in *SI Appendix*, Tables S1–S6 (crossover frequency), and Dataset S1 (RT-qPCR).

To simultaneously silence multiple genes encoding heterochromatin factors that are required for deposition of H3K9me2, non-CG methylation, or H2A.W during meiosis in *A. thaliana*, we adapted the microRNA-induced gene silencing (MIGS) approach to meiosis using the promoter of meiosis-specific gene *DISRUPTION OF MEIOTIC CONTROL 1* (*DMC1*); we designated this method meiMIGS (Fig. 1*A*) (24, 50). In meiMIGS constructs, we inserted a microRNA173 (miR173) target sequence (miR173-ts, 5′-GTGATTTTTCTCTACAAGCGAA-3′) upstream of the coding sequence to be targeted; multiple miR173-ts–target constructs can be assembled as a single transcript unit (Fig. 1 *A* and *B*). The resulting transcripts are expressed during meiosis and are cleaved by the endonuclease activity of the ARGONAUTE 1 (AGO1)–miR173 complex, which triggers the production of multiple 22-nucleotide *trans*-acting short interfering RNAs (siRNAs) from each target sequence by RDR6 and DCL4 (50, 51). The *trans*-acting siRNAs are then incorporated into AGO1 and the AGO1–siRNA complex silences their corresponding endogenous target transcripts (Fig. 1*A*). We generated *meiMIGS* transgenic plants in *A. thaliana* fluorescence-tagged lines (FTLs) with expression of the fluorescent reporters in seed or pollen. These two FTLs harbor two transgenes within the pericentromeric regions on chromosome 3, which are enriched in H3K9me2, H2A.W, DNA methylation, H3K27me1, linker histone H1, and transposons (Fig. 1 *C* and *D*). Hemizygous FTL plants carry the two transgenes in *cis* on the same chromosome 3, each expressing a fluorescent reporter gene (*GFP* or *RFP*) in the seed coat under the control of the *Napin A* (*NapA*) promoter or in pollen grains under the *LAT52* promoter (52–54). Meiotic crossover between the two *cis*-linked T-DNAs produces recombinant seeds or pollen grains with a single color. Approximately 1,000 to 1,500 seeds from the individual seed-based FTL hemizygous plant Columbia-0 *Traffic Line 3.9* (*CTL3.9*) can be automatically scored to measure crossover frequency (cM) by analyzing the frequency of each type of fluorescent seed using CellProfiler (Fig. 1*D*) (20, 27). For pollen, the crossover frequency can be automatically calculated using DeepTetrad, a deep learning-based image recognition pipeline for high-throughput pollen tetrad analysis in the *CEN3 YR/++* in the *quartet* (*qrt*) mutant background, where tetrads derived from meiotic divisions remain attached, allowing a true tetrad analysis (Fig. 1*D*) (55).

We wondered whether our meiMIGS system might increase the frequency of heterochromatic crossovers by simultaneously silencing *SUVH4*, *SUVH5*, and *SUVH6*, (30) (Fig. 1 *B* and *D*). To this end, we generated *meiMIGS-SUVH4/5/6* transgenic plants in the *CTL3.9/++* line and the *CEN3/++* FTL, as their intervals contain high levels of H3K9me2, DNA methylation, and H2A.W (Fig. 1 *C* and *D*). Similar to the *suvh4 suvh5 suvh6* triple mutant (30), independent *meiMIGS-SUVH4/5/6* T_1_ plants showed higher crossover frequencies in the *CTL3.9* and *CEN3* lines compared to wild-type (WT) plants, as did their T_2_ progeny (Fig. 1 *E* and *F* and *SI Appendix*, Tables S1 and S2). H3K9me2 is closely associated with DNA methylation in the CHG context, which is catalyzed by CMT3 (42–44). The *cmt3* mutant had an elevated number of heterochromatic crossovers similar to that observed in *suvh4*,*5*,*6*, but to a slightly lesser extent (30). CMT2, a CMT3 paralog, is required to maintain H3K9me2-dependent CHH methylation at heterochromatic regions (41, 56). Therefore, we generated *meiMIGS* plants that silenced both *CMT3* and *CMT2* (*meiMIGS-CMT3/2*), which also showed increased crossover frequencies (cM) in the *CTL3.9* and *CEN3* (Fig. 1 *G* and *H* and *SI Appendix*, Tables S3 and S4). Using RT-qPCR analysis, we confirmed that the *meiMIGS-SUVH4/5/6* and *meiMIGS-CMT3/2* transgenes are effective in silencing their endogenous targets in unopened floral buds (Fig. 1 *E*–*H* and Dataset S1). These results indicate that knockdown of *SUVH4/5/6* or *CMT3/2* genes by meiMIGS increases the heterochromatic crossovers, similar to that in the *suvh4 suvh5 suvh6* and *cmt3* mutants (30).

The histone H2A variant H2A.W specifically marks heterochromatin and contributes to heterochromatin compaction in *A. thaliana* (37, 38, 40, 48). To investigate the effect of H2A.W on heterochromatic crossover formation, we generated *meiMIGS-H2A.W.6/7/12* plants with simultaneous silencing of all three *H2A.W* genes in *CTL3.9/++* and *CEN3/++* hemizygous plants (Fig. 1 *B*–*D*, *I*, and *J*). Similar to *meiMIGS-SUVH4/5/6* and *meiMIGS-CMT3/2* transgenic plants, *meiMIGS-H2A.W.6/7/12* plants exhibited increased *CTL3.9* and *CEN3* crossover frequencies compared to the WT (Fig. 1 *I* and *J* and *SI Appendix*, Tables S5 and S6). RT-qPCR analysis determined that the transcript levels of all three *H2A.W* genes in unopen floral buds were decreased in the *meiMIGS-H2A.W.6/7/12* plants compared to the WT (Fig. 1 *I* and *J* and Dataset S1), suggesting that H2A.W.6, H2A.W.7, and H2A.W.12 are required to limit crossovers in heterochromatic regions.

### Genome-Wide Mapping of Crossovers in *meiMIGS-H2A.W.6/7/12* Progeny.

**Fig. 2.**
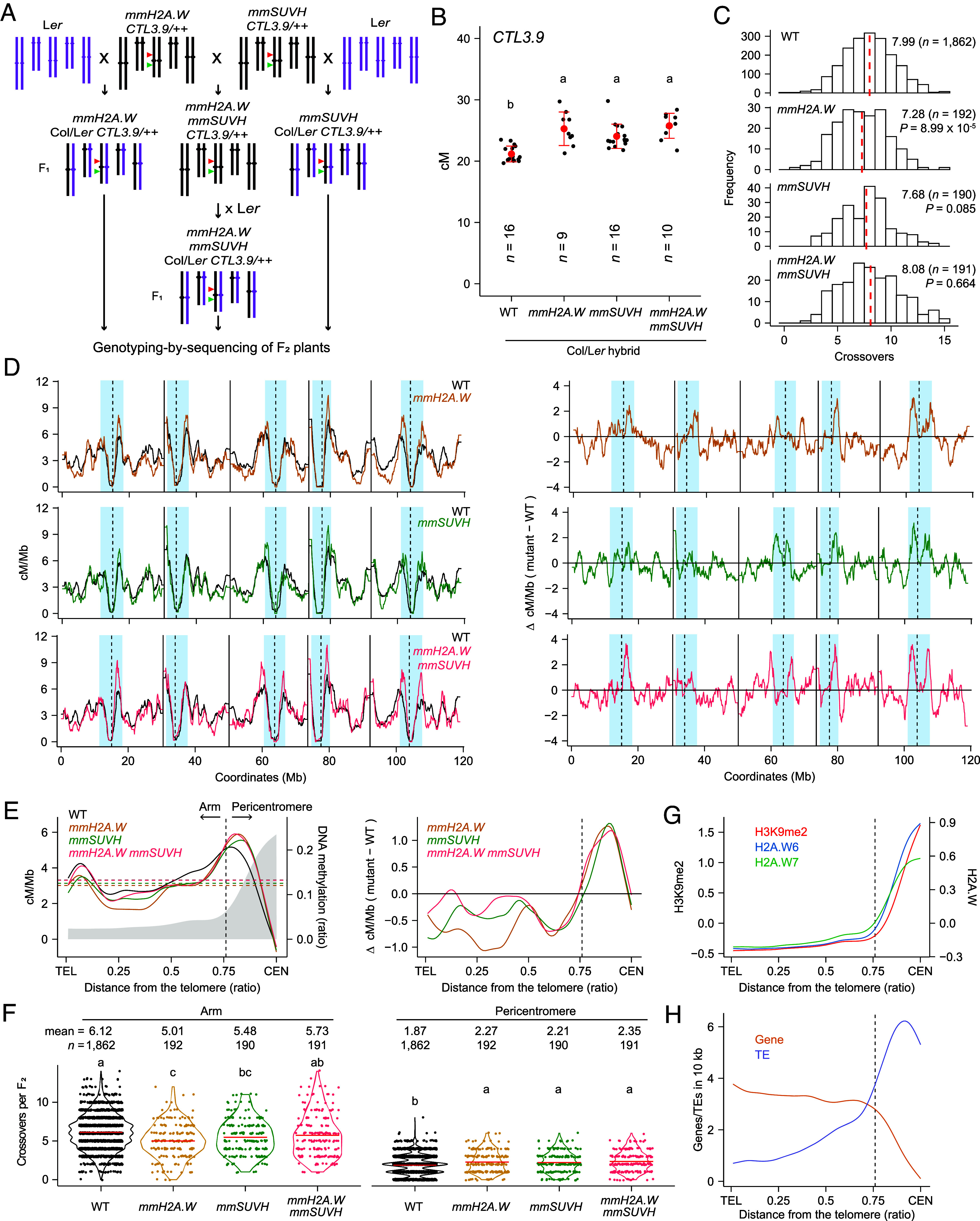
Genomic mapping of crossovers in *meiMIGS-H2A.W.6/7/12*. (*A*) Crossing scheme for GBS using *CTL3.9/++ meiMIGS-H2A.W.6/7/12* (*mmH2A.W*), *meiMIGS-SUVH4/5/6* (*mmSUVH*), *mmH2A.W mmSUVH*, and L*er* Green and red triangles indicate *CTL3.9* fluorescent reporters. (*B*) *CTL3.9* crossover frequencies (cM) in F_1_ hybrid from WT, *mmH2A.W, mmSUVH,* and *mmH2A.W mmSUVH* Col-0 × L*er* plants. Data are presented as means ± SD of individual F_1_ plants (*n* = number of plants). (*C*) Distribution of crossover numbers per F_2_ individual for WT and *meiMIGS* populations by GBS. Red dashed lines indicate mean values. Significance was determined by Welch’s *t* test. (*D*) Normalized frequency of crossovers (cM/Mb) mapped along the five *A. thaliana* chromosomes in WT (black), *mmH2A.W* (brown)*, mmSUVH* (green), and *mmH2A.W mmSUVH* (pink) (*Left*) and differential plots (meiMIGS − WT = Δ cM/Mb) (*Right*). Centromeres (dashed lines), telomeres (solid lines), and pericentromeres (blue shading) are indicated. (*E*) Crossover frequency (cM/Mb) along chromosome telomere (TEL)-to-centromere (CEN) axes in WT and *meiMIGS* populations (*Left*) and differential plot (meiMIGS − WT = Δ cM/Mb) (*Right*). The dashed line marks the chromosome arm–pericentromere border. (*F*) Number of crossovers in chromosome arms and pericentromeric regions per F_2_ plant. Black and colored dots indicate individual plants and red lines represent mean values. (*G* and *H*) As for (*E*), but showing H3K9me2, H2A.W.6, and H2A.W.7 ChIP-seq signals (*G*), and gene and transposable element (TE) enrichment (*H*). For (*B*, *C*, and *F*), significance (*P* < 0.05) was determined by ANOVA with Tukey’s HSD (*B*) or Games–Howell test (*F*); datasets in Dataset S2.

To elucidate the genome-wide effects of *H2A.W* and/or *SUVH* knockdown in the *meiMIGS-H2A.W.6/7/12* (*mmH2A.W*) and *meiMIGS-SUVH4/5/6* (*mmSUVH*) transgenes on the position of crossovers in the context of hybrids derived from crosses between the *A. thaliana* accessions Col-0 and L*er*, we performed genotyping-by-sequencing (GBS) to generate genomic crossover maps at high-resolution (Fig. 2). More precisely, we crossed *mmH2A.W CTL3.9/++*, *mmSUVH CTL3.9/++*, and *mmH2A.W mmSUVH CTL3.9/++* Col-0 primary transgenic plants with L*er*, to produce *meiMIGS CTL3.9/++* Col-0 × L*er* F_1_ hybrid plants (Fig. 2*A*). We then allowed these *meiMIGS* F_1_ hybrid plants to self-pollinate and measured their crossover frequency within the *CTL3.9* interval by analyzing the segregation of fluorescent F_2_ seeds from individual F_1_ hybrid plants (Fig. 2*B*). We observed a significant increase in *CTL3.9* crossover frequencies in the progeny of *mmH2A.W* and *mmSUVH* Col-0 × L*er* F_1_ hybrids compared to that from Col-0 × L*er* F_1_ hybrids (*SI Appendix*, Table S7 and Dataset S2), which was similar to the increase observed in the progeny of self-pollinated *CTL3.9/++* Col-0 plants (Figs. 1 *E* and *I* and 2 *B*). The *mmH2A.W mmSUVH* Col-0 × L*er* F_1_ hybrids showed a similarly elevated rate of crossover frequency within the *CTL3.9* interval as *mmH2A.W* Col-0 × L*er* and *mmSUVH* Col-0 × L*er* F_1_ hybrid plants compared to Col-0 × L*er* F_1_ hybrids. This result suggests that meiotic knockdown of both *H2A.W* and *SUVH4/5/6* genes increases pericentromeric crossovers in the Col-0 × L*er* hybrid, similar to silencing either the *H2A.W* genes or the *SUVH4/5/6* genes.

GBS-based crossover mapping analysis allowed us to query the recombination landscape outside of the *CTL3.9* interval in *meiMIGS* F_2_ individuals. We determined that the mean number of crossovers per F_2_ individual does not change in *mmSUVH* or *mmH2A.W mmSUVH* F_2_ progeny, but moderately decreased in *mmH2A.W.* F_2_ progeny compared to Col-0 × L*er* F_2_ progeny (Fig. 2*C* and Table 1). To investigate the significance of the change in genome-wide crossover distribution between *meiMIGS* transgenic plants and their nonsilenced controls, we combined GBS datasets for Col-0 × L*er* F_2_ individuals from our (*n* = 240) and other groups (*n* = 1,622) (20, 24, 28, 57). These two independent GBS datasets showed similar mean numbers of crossovers per F_2_ individual and similar genomic landscapes (*SI Appendix*, Fig. S1). To analyze genomic crossover distribution, we defined centromeres as regions surrounding gaps in the chromosome assembly where crossovers are absent. Pericentromeres are the contiguous regions flanking the centromeres and exhibiting higher DNA methylation than the genome-wide average, while chromosome arms represent the remainder of the genome (30). Consistent with the *CTL3.9* data obtained from the progeny of *CTL3.9*/++ Col-0 plants (Fig. 1 *E*–*J*), GBS analysis showed that the number of crossovers increased in the pericentromeric regions in *mmH2A.W*, *mmSUVH*, and *mmH2A.W mmSUVH* Col-0 × L*er* F_2_ individuals as compared to Col-0 × L*er* F_2_ controls (Fig. 2 *D*–*F*, Table 1 and Dataset S2), demonstrating that H2A.W is required to suppress pericentromeric crossovers, similar to H3K9m2. We confirmed that the silencing imposed by the *mmH2A.W* and *mmSUVH* silencing cassettes does not cause an additive increase in heterochromatic crossovers relative to that seen with either *mmH2A.W* or *mmSUVH* (Fig. 2*F* and Dataset S2). The number of crossovers in chromosome arms was lower in *mmH2A.W* and *mmSUVH* than in Col-0 × L*er* F_2_ controls (Fig. 2*F* and Dataset S2), suggesting that meiotic knockdown of *H2A.W.6/7/12* or *SUVH4/5/6* reshapes the landscape of crossovers along chromosomes, increasing crossovers in pericentromeric regions while decreasing crossovers in chromosome arms, likely due to crossover interference (*SI Appendix*, Fig. S5 *A* and *B*). In addition, simultaneous knockdown of *H2A.W.6/7/12* and *SUVH4/5/6* in *mmH2A.W mmSUVH* individuals increased the number of heterochromatic crossovers compared to Col-0 × L*er* F_2_ controls, but did not decrease the number of chromosome arm-associated crossovers, unlike for *mmH2A.W* and *mmSUVH* (Fig. 2*F*, Table 1 and Dataset S2). Consistent with the increased number of heterochromatic crossovers in *mmH2A.W*, *mmSUVH*, and *mmH2A.W mmSUVH*, the pericentromeric regions containing additional crossovers overlapped with TE-rich heterochromatic regions densely marked by H2A.W.6, H2A.W.7, and H3K9me2 (Fig. 2 *G* and *H*). Collectively, our GBS analysis of crossover maps in *mmH2A.W* Col-0 × L*er* F_2_ individuals demonstrates that meiotic silencing of *H2A.W* genes leads to more crossovers in heterochromatic pericentromeric regions.

**Table 1.** GBS analysis for the number and positions of genomic crossovers

Col-0 × L*er* genotype (*n*)	Total				Chromosome arms				Pericentromeric regions			
	COs	Mean	SD	*P-*value	COs	Mean	SD	*P-*value	COs	Mean	SD	*P-*value
WT (1,862)	14,883	7.99	2.31	1.000	11,395	6.12	2.20	1.000	3,488	1.87	1.23	1.000
*mmH2A.W* (192)	1,397	7.28	2.38	8.99 × 10^−5^	961	5.01	2.28	5.88 × 10^−10^	436	2.27	1.41	2.16. × 10^−4^
*mmSUVH* (190)	1,460	7.68	2.34	0.085	1,041	5.48	2.25	2.25 × 10^−4^	419	2.21	1.33	1.10. × 10^−3^
*mmH2A.W mmSUVH* (191)	1,544	8.08	2.78	0.664	1,095	5.73	2.79	0.064	449	2.35	1.32	3.05 × 10^−6^
*h2a.w.6* (191)	1,370	7.17	2.12	8.58 × 10^−7^	952	4.98	1.94	6.28 × 10^−13^	418	2.19	1.30	1.58. × 10^−3^
*h2a.w.7* (191)	1,409	7.38	2.14	2.19 × 10^−4^	994	5.20	1.88	1.26 × 10^−9^	415	2.17	1.30	2.66 × 10^−3^
*h2a.w.12* (192)	1,429	7.44	1.97	3.61 × 10^−4^	1,047	5.45	1.79	2.61 × 10^−6^	382	1.99	1.31	0.240
*h2a.w.6*,*7* (192)	1,404	7.31	2.25	8.96 × 10^−5^	950	4.95	2.20	2.37 × 10^−11^	454	2.36	1.37	3.24 × 10^−6^
*h2a.w.6*,*7*,*12* (287)	2,031	7.08	2.30	9.50 × 10^−10^	1,319	4.60	2.34	8.57 × 10^−24^	712	2.48	1.40	1.80 × 10^−11^

WT: Col-0 × L*er* F_2_; *mmH2A.W*: *meiMIGS-H2A.W.6/7/12* (Col-0) × L*er* F_2_; *mmSUVH*: *meiMIGS-SUVH4/5/6* (Col-0) × L*er* F_2_; *mmH2A.W mmSUVH*: *meiMIGS-H2A.W.6/7/12 meiMIGS-SUVH4/5/6* (Col-0) × L*er* F_2_; *h2a.w.6*: *h2a.w.6-3* (Col-0) × *h2a.w.6-4* (L*er*) F_2_; *h2a.w.7*: *h2a.w.7-3* (Col-0) × *h2a.w.7-4* (L*er*) F_2_; *h2a.w.12*: *h2a.w.12-3* (Col-0) × *h2a.w.12-4* (L*er*) F_2_; *h2a.w.6*,*7*: *h2a.w.6-3 h2a.w.7-3* (Col-0) × *h2a.w.6-4 h2a.w.7-4* (L*er*) F_2_; *h2a.w.6,7,12*: *h2a.w.6-3 h2a.w.7-3 h2a.w.12-3* (*h2a.w-3* in Col-0) × *h2a.w.6-4 h2a.w.7-4 h2a.w.12-4* (*h2a.w-4* in L*er*) F_2_. *n*: Number of F_2_ plants analyzed; COs: Crossovers identified by GBS; SD: Standard deviation. Significant differences were determined with Welch′s *t*-test.

### *H2A.W.6* and *H2A.W.7* Are Required to Suppress Heterochromatic Crossovers.

To gain genetic insight into how single and combinations of the *H2A.W.6*, *H2A.W.7*, and *H2A.W.12* genes affect heterochromatic crossovers, we crossed the *CTL3.9* reporter in the Col-0 background to the *h2a.w-2* triple null mutant (*h2a.w.6-2 h2a.w.7-1 h2a.w.12-1*) (48), allowing F_1_ plants to self-pollinate and produce F_2_ seeds. We then measured crossover frequencies (in centimorgans [cM]) over the *CTL3.9* interval in the *h2a.w.6*, *h2a.w.7*, *h2a.w.12*, *h2a.w.6 h2a.w.7* (*h2a.w.6*,*7*), *h2a.w.6*,*12*, *h2a.w.7*,*12*, and *h2a.w.6,7,12* (*h2a.w-2*) backgrounds with loss of function of one, two, or all three H2A.Ws (Table 2 and *SI Appendix*, Table S8 and Dataset S3). Crossover frequencies in the *CTL3.9* interval were increased in *h2a.w.6*, *h2a.w.7*, *h2a.w.6*,*7*, *h2a.w.6*,*12*, *h2a.w.7*,*12*, and *h2a.w.6,7,12*, but not in *h2a.w.12,* compared to the WT (Table 2 and *SI Appendix*, Table S8 and Dataset S3). Similarly, we observed an increased crossover frequency (cM) over the *CEN3* genetic interval as *CTL3.9* in F_2_ individuals of the *h2a.w.6*, *h2a.w.7*, *h2a.w.6*,*7*, and *h2a.w.6*,*7*,*12*, compared to the WT Col-0 (*SI Appendix*, Fig. S2 and Table S9). These results indicate that the genetic disruption of either *H2A.W.6* or *H2A.W.7*, or both, leads to a similar increase in heterochromatic crossovers. Thus, it is likely that *H2A.W.6* and *H2A.W.7* function redundantly to limit heterochromatic crossovers, with each gene individually playing a significant role. By contrast, *H2A.W.12* appears to be dispensable for limiting heterochromatic crossovers. Consistent with the heterochromatic crossover phenotype observed in the *h2a.w* mutants, our analysis of RNA-sequencing data from male meiocytes, unopened floral buds, and seedlings revealed that *H2A.W.6* and *H2A.W.7* are more abundantly expressed than *H2A.W.12* (*SI Appendix*, Fig. S3) (20, 28, 58), supporting a major role for H2A.W.6 and H2A.W.7 in suppressing heterochromatic crossovers.

**Table 2.** Crossover frequencies (cM) over the *CTL3.9* genetic interval in WT and *h2a.w* mutants

Genotype	*n*	cM (mean ± SD)	Groups (Tukey’s test)
WT	36	17.90 ± 1.11	c
*h2a.w.6*	13	19.48 ± 1.33	ab
*h2a.w.7*	22	19.25 ± 0.97	ab
*h2a.w.12*	12	18.53 ± 1.06	bc
*h2a.w.6,7*	10	20.53 ± 1.21	a
*h2a.w.6,12*	8	19.37 ± 1.43	ab
*h2a.w.7,12*	14	19.54 ± 1.06	ab
*h2a.w.6,7,12* (*h2a.w-2*)	9	20.44 ± 1.12	a
*h2a.w.6,7,12* (*h2a.w-3*)	15	20.29 ± 0.64	a
*h2a.w-2 × h2a.w-3*	16	20.04 ± 0.64	a

*n*: Number of plants measured; WT: Wild type (Col-0); SD: Standard deviation. Groups with different letters indicate statistically significant differences (ANOVA with Tukey–Kramer′s test).

### Heterochromatic Crossovers Are Increased in *h2a.w* Mutants.

**Fig. 3.**
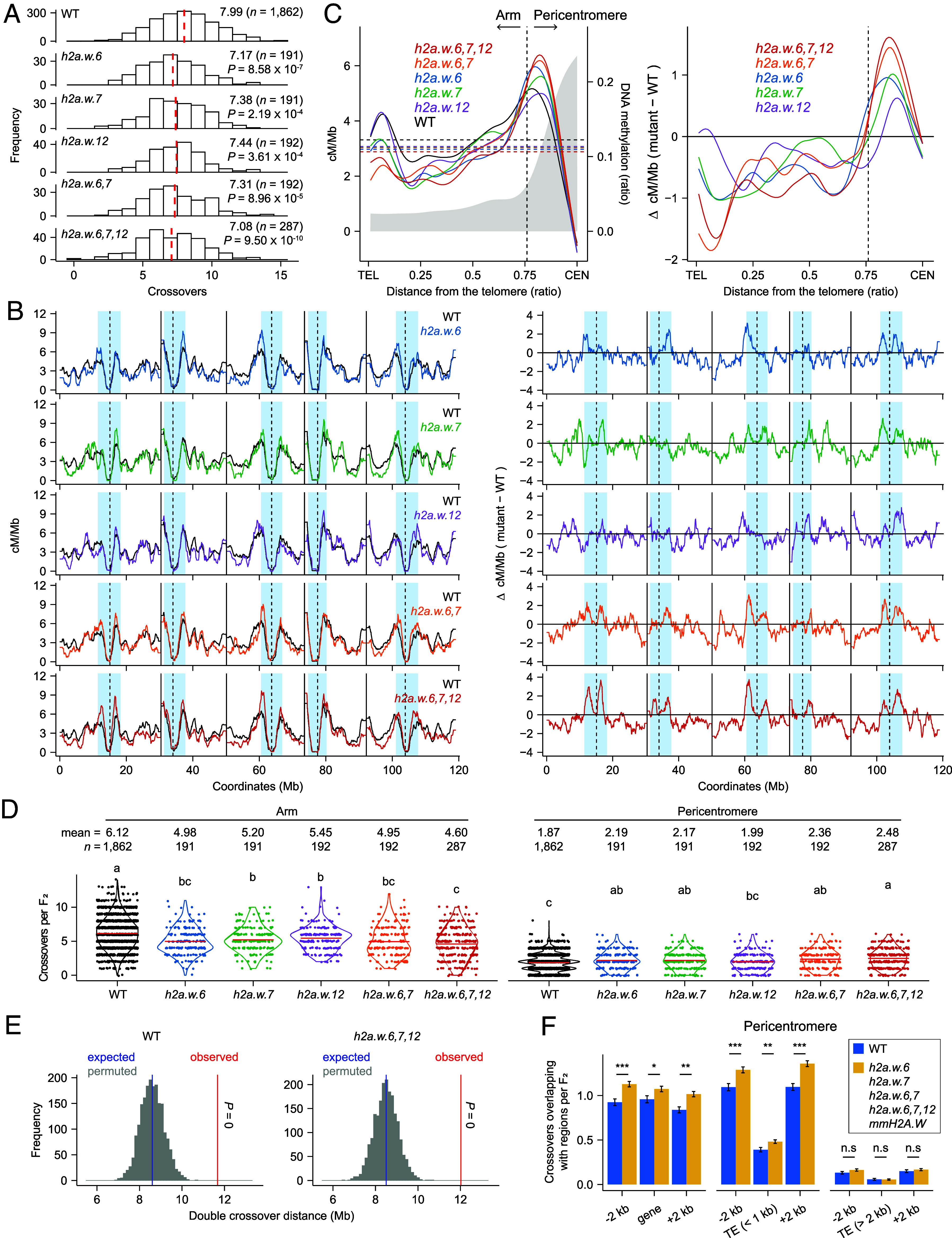
Genome-wide crossover maps in *h2a.w* mutants. (*A*) Distribution of crossover numbers per F_2_ individual for WT, *h2a.w.6*, *h2a.w.7*, *h2a.w.12*, *h2a.w.6,7*, and *h2a.w.6*,*7*,*12* Col-0 × L*er* hybrids. Dashed red lines indicate mean crossover numbers (*n* = F_2_ individuals). Significance was determined by Welch’s *t* test. (*B*) Normalized crossover frequency (cM/Mb) along the five *A. thaliana* chromosomes on a continuous *x*-axis in WT (black), *h2a.w.6* (blue)*, h2a.w.7* (green)*, h2a.w.12* (purple), *h2a.w.6*,*7* (brown), and *h2a.w.6*,*7*,*12* (red) F_2_ individual plants (*Left* panels). Differential plots (mutant − WT = Δ cM/Mb) are also shown (*Right* panels). Dashed lines indicate the centromere assembly gaps; solid lines mark telomeres. (*C*) Crossover frequency (cM/Mb) along chromosome telomere (TEL)-to-centromere (CEN) axes in WT, *h2a.w* mutants (*Left*) and differential plot (mutant − WT = Δ cM/Mb) (*Right*). (*D*) Number of crossovers along chromosome arms (*Left*) and pericentromeric regions (*Right*) per F_2_ individual. Black and colored dots indicate individual plants; red lines show mean values (*n* = F_2_ individuals). Significant differences were determined by one-way ANOVA with Games–Howell (*P* < 0.05). (*E*) Observed mean physical distances (Mb) of double crossovers (red line) in WT and *h2a.w.6*,*7*,*12*, compared to 2,000 random sets (gray lines). Blue lines indicate random mean distances; *P*-values were calculated by permutation tests. (*F*) Number of pericentromeric crossovers overlapping the separate gene and TE features in F_2_ individuals of *mmH2A.W* lines and *h2a.w* mutants, compared to WT. Data are shown as means ± SE. Asterisks indicate significance (*P < 0.05, **P < 0.01, ***P < 0.001; Wilcoxon test).

We investigated how single and compound mutations in the *H2A.W.6, H2A.W.7,* and *H2A.W.12* genes affect heterochromatic crossovers at high resolution using GBS-based crossover mapping (Fig. 3 *A*–*D* and Table 1). To prepare the populations for crossover mapping in *h2a.w* mutants, we generated new *h2a.w* deletion alleles using CRISPR/ Cas9-mediated mutagenesis in the Col-0 and L*er* backgrounds (*SI Appendix*, Fig. S4 *A* and *B*). We designed a pair of single guide RNAs to induce a loss-of-function deletion mutation in each of *H2A.W.6*, *H2A.W.7*, and *H2A.W.12*. We tested for a deletion in each of the *H2A.W* genes by PCR amplification followed by gel electrophoresis analysis and Sanger sequencing (*SI Appendix*, Fig. S4 *A* and *B* and Dataset S4). Using the *CTL3.9* line, we observed that the *CTL3.9* crossover frequency was increased in the new *h2a.w.6-3 h2a.w.7-3 h2a.w.12-3* triple mutant in the Col-0 background (*h2a.w-3*) and in *h2a.w-2* × *h2a.w-3* F_1_ plants compared to the WT Col-0, similar to *h2a.w-2* (Table 2, *SI Appendix*, Table S8 and Dataset S3), indicating that *h2a.w-3* is a triple loss-of-function allele, like *h2a.w-2*. We also confirmed that the *CEN3* crossover frequency was increased in *h2a.w.6-3* and *h2a.w.7-3* but not in *h2a.w.12-3* compared to the WT Col-0 (*SI Appendix*, Fig. S4*C* and Dataset S5). We crossed the *h2a.w* Col-0 alleles to the new *h2a.w.6-4*, *h2a.w.7-4*, *h2a.w.12-4*, *h2a.w.6*,*7-4*, and *h2a.w.6*,*7*,*12-4* (*h2a.w-4*) mutants in the L*er* background to generate *h2a.w.6-3* × *h2a.w.6-4*, *h2a.w.7-3* × *h2a.w.7-4*, *h2a.w.12-3* × *h2a.w.12-4, h2a.w.6*,*7-3* × *h2a.w.6*,*7-4*, and *h2a.w-3* × *h2a.w-4* Col-0 × L*er* F_1_ hybrids. We allowed these *h2a.w* Col-0 × L*er* F_1_ hybrids to self-pollinate, and performed GBS on their resulting F_2_ individuals to map crossovers genome-wide (Fig. 3 *A*–*D*, Table 1 and *SI Appendix*, Table S10).

An analysis of crossovers mapped by GBS of F_2_ individuals indicated that the mean number of crossovers per F_2_ individual was lower in the *h2a.w.6*, *h2a.w.7*, *h2a.w.12*, *h2a.w.6*,*7*, and *h2a.w.6*,*7*,*12* backgrounds than in F_2_ individuals derived from Col-0 × L*er* F_1_ hybrids, which was consistent with the results obtained with the *mmH2A.W* transgenes (Fig. 3*A*). When we plotted and quantified the crossovers on all five *A. thaliana* chromosomes (Fig. 3*B*), from telomeres to centromeres (Fig. 3*C*), and on chromosome arms and pericentromeres (Fig. 3*D*), we found fewer chromosome arm-associated euchromatic crossovers and more pericentromere-associated heterochromatic crossovers in the *h2a.w.6*, *h2a.w.7*, *h2a.w.6*,*7*, and *h2a.w.6*,*7*,*12* backgrounds compared to the WT Col-0 × L*er* F_2_ plants (Fig. 3 *B*–*D*, Table 1 and Dataset S3). We observed a similar increase in crossover numbers in the pericentromeric regions of *h2a.w.6* and *h2a.w.7* as in *h2a.w.6*,*7* and *h2a.w.6*,*7*,*12*, but no additive increase in *h2a.w.6*,*7* and *h2a.w.6*,*7*,*12* (Fig. 3 *C* and *D* and Dataset S3). These findings support the redundant effects of *H2A.W.6* and *H2A.W.7* mutations on heterochromatic crossover formation, with each having a significant individual effect, as earlier observed over the *CTL3.9* interval (Table 2). By contrast, *h2a.w.12* did not show a significant increase in the number of heterochromatic crossovers relative to WT Col-0 × L*er* F_2_ plants (Fig. 3*D* and Dataset S3), consistent with the *CTL3.9* crossover frequency measured in the *h2a.w.12* Col-0 background (Table 2).

To assess the consequences of H2A.W loss of function on crossover interference, we measured the physical distance between *cis* double crossovers along the same chromosomes using the F_2_ GBS data for WT Col-0 × L*er* hybrids, *meiMIGS* lines, and the *h2a.w* mutant hybrids. We observed that crossovers are more evenly spaced in the *h2a.w* mutants, *mmH2A.W*, *mmSUVH*, *mmH2A.W mmSUVH*, and WT but not in *recq4a recq4b* with increased noninterfering crossovers, compared to 2,000 sets of randomly distributed crossover distances (permutation test, all *P* < 0.006) (Fig. 3*E* and *SI Appendix*, Fig. S5*A*). Additionally, the presence of crossovers in pericentromeric regions led to a decrease in crossovers in the same chromosome arms across all tested genotypes, except for *recq4a recq4b,* where the predominant noninterfering crossovers in chromosome arms remained unaffected regardless of crossovers in pericentromeric regions (*SI Appendix*, Fig. S5*B*). These results suggest that crossover interference is active and may result in fewer crossovers in chromosomal arms of *h2a.w* mutants, due to increased heterochromatic crossovers. Together, our analysis of crossover maps in the *h2a.w* mutants demonstrates that H2A.W.6 and H2A.W.7 are required to suppress heterochromatic crossovers and that the *h2a.w.6*, *h2a.w.7*, *h2a.w.6*,*7*, and *h2a.w.6*,*7*,*12* mutants form more heterochromatic crossovers and fewer chromosome arm-associated crossovers than their WT controls.

### Heterochromatic Crossovers Are Increased Near Genes and Short TEs in *h2a.w* Mutants.

Using high-resolution GBS data from F_2_ individuals derived from crosses between Col-0 and L*er* with mean genomic coverage greater than 1.5 (WT, *n* = 622, mean resolution, 1,670 bp; *mmH2A.W* and *h2a.w* mutants, *n* = 1,053, mean resolution, 1,744 bp), we examined whether the additional pericentromeric crossovers in *mmH2A.W* lines and *h2a.w* mutants occur near genes or TEs in heterochromatic regions. We observed increased numbers of pericentromeric crossovers in F_2_ individuals derived from the *mmH2A.W* lines and *h2a.w* mutants upstream (−2 kb) and downstream (+2 kb) of the genes, in addition to the gene bodies, compared to WT Col-0 × L*er* F_2_ individuals (Fig. 3*F*), which was consistent with the recent observation that heterochromatic crossovers in WT occur predominantly around genes embedded in pericentromeric regions, with even more crossovers in *cmt3* (29). Furthermore, we found there were more heterochromatic crossovers in *mmH2A.W* and *h2a.w* mutants compared to WT upstream and downstream of TEs shorter than 1 kb in size, but not for TEs longer than 2 kb (Fig. 3*F*). Short TEs account for approximately 15% of all TEs, with 72% classified as DNA TEs and 28% as RNA TEs (*SI Appendix*, Fig. S6*A*). Among the short TEs associated with pericentromeric crossovers, the majority (~83%) were DNA TEs, including Helitrons and MuDR, while a smaller proportion (~17%) were RNA TEs (*SI Appendix*, Fig. S6*B* and Dataset S6). Notably, short Helitrons were more frequently associated with crossovers than expected, whereas the association of Gypsy RNA TEs with crossovers was lower than expected (*SI Appendix*, Fig. S6*C*). Consistent with the above observation, small euchromatic DNA TEs are closely associated with gene regulatory regions and meiotic DSB hotspots (35). Therefore, our GBS results suggest that, like *cmt3* (29), *mmH2A.W* plants and *h2a.w* mutants produce more heterochromatic crossovers that occur around genes and short TEs in the pericentromeric regions.

### Heterochromatin Accessibility Is Increased in *h2a.w* Mutants.

**Fig. 4.**
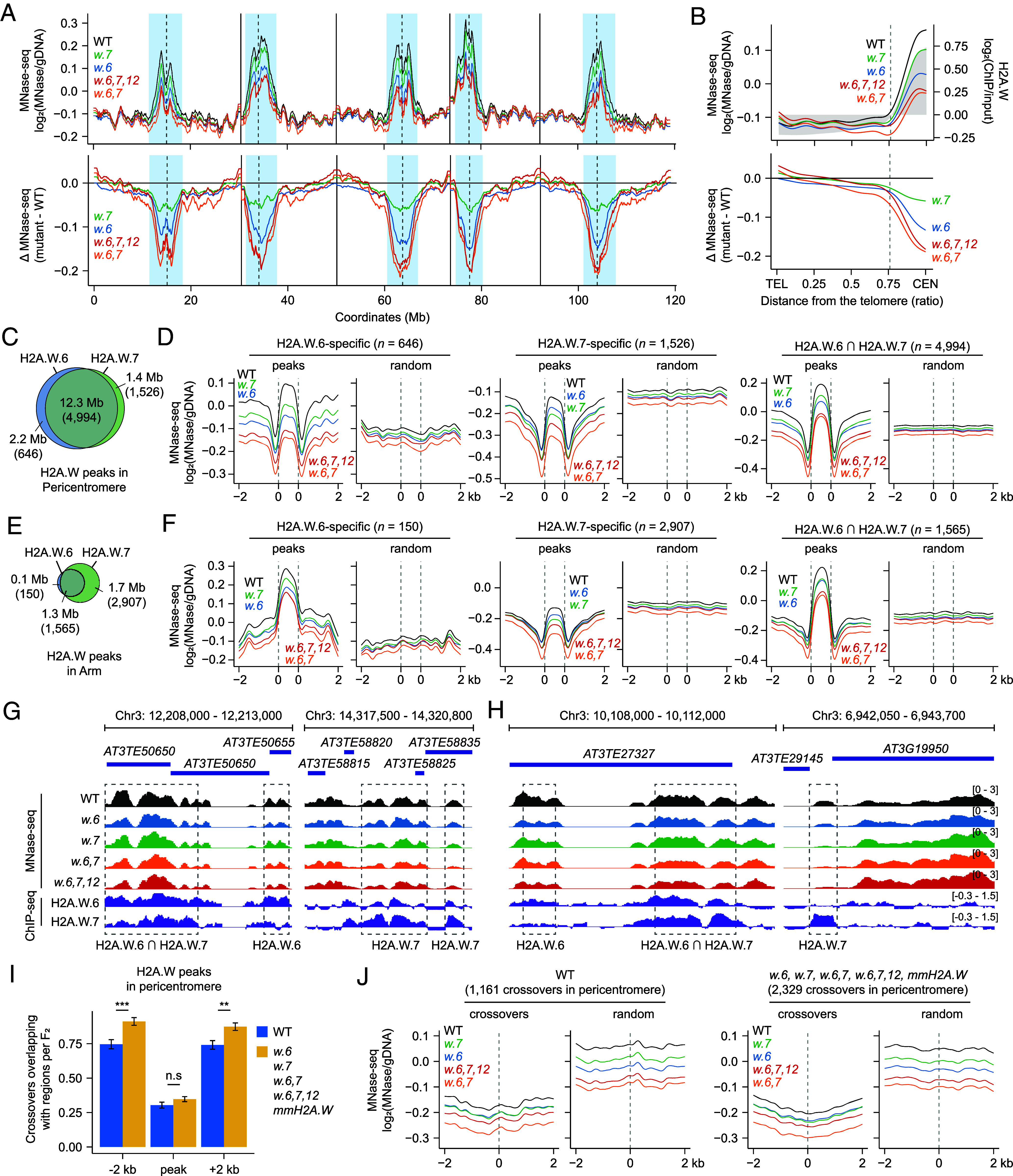
Genome-wide nucleosome density mapping in *h2a.w* mutants. (*A*) Normalized MNase-seq density (log_2_[MNase-seq/gDNA]) along *A. thaliana* chromosomes in WT Col-0 (black), *h2a.w.6-3* (*w.6*, blue)*, h2a.w.7-3* (*w.7*, green)*, h2a.w.6*,*7* (*w.6*,7, orange), and *h2a.w.6,7,12* (*w.6,7,12*, red) mutants (*Upper*), with differential plot (mutant − WT = Δ MNase-seq) (*Lower*). (*B*) MNase-seq density along chromosome TEL-to-CEN axes in WT and *h2a.w* mutants (*Upper*) and differential plot (mutant − WT = Δ MNase-seq) (*Lower*). Averaged H2A.W ChIP-seq data are in gray. The dashed line indicates chromosome arm–pericentromere border. (*C*) Venn diagram showing overlap between H2A.W.6 and H2A.W.7 ChIP-seq peaks in pericentromeres. (*D*) Metaplots of MNase-seq density for WT and *h2a.w* mutants in a 4-kb window around H2A.W.6/7 ChIP-seq peaks (shared or specific) in pericentromeres, compared to random positions. (*E* and *F*) As for (*C*) and (*D*), respectively, but showing chromosome arms. (*G* and *H*) Representative IGV snapshots of MNase-seq density and H2A.W.6/7 ChIP-seq [log_2_(ChIP-seq/input)] in pericentromeric (*G*) and chromosome arm (*H*) regions. Dashed boxes highlight peaks specific to H2A.W.6, H2A.W.7, or shared peaks. (*I*) Number of pericentromeric crossovers per F_2_ individual of *h2a.w* mutants and *mmH2A.W* near H2A.W.6/7 ChIP-seq peaks. Data are shown as means ± SE. Asterisks indicate significance (*P < 0.05, **P < 0.01, ***P < 0.001; Wilcoxon test). (*J*) Metaplots of nucleosome densities around pericentromeric crossover midpoints in WT, *h2a.w* mutants, and *mmH2A.W* lines, compared to random positions.

H2A.W variants facilitate the compaction of constitutive heterochromatin via their C-terminal DNA-binding KSPK motif (37, 38). To investigate whether and how the *h2a.w.6*, *h2a.w.7*, *h2a.w.6*,*7*, and *h2a.w.6*,*7*,*12* mutants with a greater number of heterochromatic crossovers affect nucleosome density and stability in the pericentromeric regions, we performed micrococcal nuclease sequencing (MNase-seq) in flower buds (< ~2 mm or ~0.3 to 0.5 mm) containing meiocytes, as well as in 10-d-old seedlings, and measured genome-wide chromatin accessibility in the *h2a.w* mutants along with the WT Col-0 (Fig. 4 and *SI Appendix*, Figs. S7 and S8). It is important to note that the smaller flower buds (~0.3 to 0.5 mm) contain more meiocytes compared to the larger buds (< ~2 mm). However, both sizes contain only a small proportion of meiotic cells and do not exclusively represent meiocytes, as the majority of their cells are somatic. A heatmap and clustering analysis showed that all three biological replicates of MNase-seq libraries for each genotype are highly correlated, with the various *h2a.w* mutants forming tighter clusters, compared to the WT (*SI Appendix*, Fig. S7). Genome-wide analysis of MNase-seq reads revealed that the *h2a.w.6*, *h2a.w.7*, *h2a.w.6*,*7*, and *h2a.w.6,7,12* mutants exhibit a lower nucleosome density and thus increased chromatin accessibility in pericentromeric regions compared to the WT in small flower buds as well as seedlings (Fig. 4 *A* and *B* and *SI Appendix*, Fig. S8), further supporting a structural role of H2A.W variants in heterochromatin compaction. Moreover, the *h2a.w.6* mutant experienced higher heterochromatin accessibility than *h2a.w.7*, which was consistent with the RNA-seq data showing that *H2A.W.6* is more highly expressed than *H2A.W.7* in seedlings, flower buds, and meiocytes (*SI Appendix*, Fig. S3) and the H2A.W ChIP-seq data showing that H2A.W.6 is more enriched at pericentromeric regions and near centromeres compared to H2A.W.7 (Fig. 2*G*). The *h2a.w.6*,*7* and *h2a.w.6,7,12* mutants also showed comparably increased heterochromatin accessibility and higher chromatin accessibility than the *h2a.w.6* and *h2a.w.7* single mutants (Fig. 4 *A* and *B* and *SI Appendix*, Fig. S8), suggesting that H2A.W.6 and H2A.W.7 play important and partially redundant roles in heterochromatin condensation in agreement with the distinct distributions of these two histone variants (39).

Most nucleosomes contain homotypic H2A variant–H2B dimers, such as two copies of H2A.W.6–H2B, but not heterotypic H2A.W.6–H2B and H2A.W.7–H2B dimers in immunoprecipitation assays (39, 47, 59). Therefore, to investigate nucleosome density and dynamics in the *h2a.w.6* and *h2a.w.7* mutants, we defined H2A.W.6- and H2A.W.7-specific ChIP-seq peaks in pericentromeric regions and chromosome arms and assessed their degree of overlap with MNase-seq reads from the *h2a.w* mutants (Fig. 4 *C*–*F* and *SI Appendix*, Table S11 and Fig. S9). By analyzing H2A.W.6/7 ChIP-seq data from seedlings (59), we found that the majority (~77.5%) of H2A.W.6 and H2A.W.7 ChIP-seq signals overlapped in heterochromatic pericentromeric regions (*SI Appendix*, Fig. S9 *A–E*). However, a subset of signals displayed partial distinctions, with some signals being specific to either H2A.W.6 (~13.7%) or H2A.W.7 (~8.8%). Additionally, we observed that nucleosomes containing H2A.W.6 and/or H2A.W.7 (~85.5%) overlapped with H3K9me2-enrich regions (*SI Appendix*, Fig. S9 *B–E*). Given that H3K9me2 is tightly associated with H2A.W variants (39, 47, 59), and deposited into heterochromatin during meiosis (30), it is likely that H2A.W proteins localize to pericentromeric regions during meiosis. We determined that the *h2a.w.6* mutant exhibits higher chromatin accessibility at the H2A.W.6-specific ChIP-seq peaks (pericentromeres *n* = 646, arms *n* = 150) than the *h2a.w.7* mutant, which conversely showed higher chromatin accessibility at the H2A.W.7-specific ChIP-seq peaks (pericentromeres *n* = 1,526, arms *n* = 2,907) than *h2a.w.6* (Fig. 4 *D* and *F*). Using the Integrative Genomic Viewer, we confirmed that the nucleosome densities of representative H2A.W.6- and H2A.W.7-specific ChIP-seq peaks, as well as shared ChIP-seq peaks corresponding to the metaplots (Fig. 4 *D* and *F* and *SI Appendix*, Fig. S9 *C* and *E*) are specifically decreased in the pericentromeric regions and chromosome arms according to the *h2a.w* genotype (Fig. 4 *G* and *H*). This suggests that H2A.W.6 and H2A.W.7 specifically contribute to dense compaction of heterochromatin. The *h2a.w.6*, *h2a.w.7*, *h2a.w.6*,*7*, and *h2a.w.6,7,12* mutants were characterized by increased heterochromatin accessibility at the H2A.W.6 and H2A.W.7 shared ChIP-seq peaks as well as the H2A.W.6- or H2A.W.7-specific peaks, compared to the WT. Moreover, the *h2a.w.6,7* and *h2a.w.6,7,12* mutants showed greater chromatin accessibility at the H2A.W.6/7 ChIP-seq peaks compared to the *h2a.w.6* and *h2a.w.7* single mutants, as also observed at a chromosome scale (Fig. 4 *D* and *F*–*H*), suggesting their partially redundant roles in condensing heterochromatin.

To further examine how increased heterochromatin accessibility is correlated with heterochromatic crossovers in *h2a.w* mutants, we plotted the pericentromeric crossovers of Col-0 × L*er* F_2_ individuals of WT, *mmH2A.W* lines, and *h2a.w* mutants upstream and downstream of a 2-kb window around the H2A.W.6/7 ChIP-seq peaks. The additional crossovers seen in the *mmH2A.W* lines and *h2a.w* mutants were enriched upstream and downstream of the H2A.W.6/7 ChIP-seq peaks, but not within the peaks (Fig. 4*I*). We observed that the nucleosome densities around the midpoints of two switching SNPs at heterochromatic crossover sites were lower in WT, *mmH2A.W* lines, and *h2a.w* mutants compared to the same number of randomly selected sites (Fig. 4*J*), indicating a tight association between heterochromatic crossovers and accessible chromatin in pericentromeric regions. Taken together, our MNase-seq analysis suggests that H2A.W.6 and H2A.W.7 play critical roles in heterochromatin condensation during meiosis.

### H3K9me2 Levels Are Decreased in Meiotic Heterochromatin of *mmH2A.W* Lines and *h2a.w* Mutants.

**Fig. 5.**
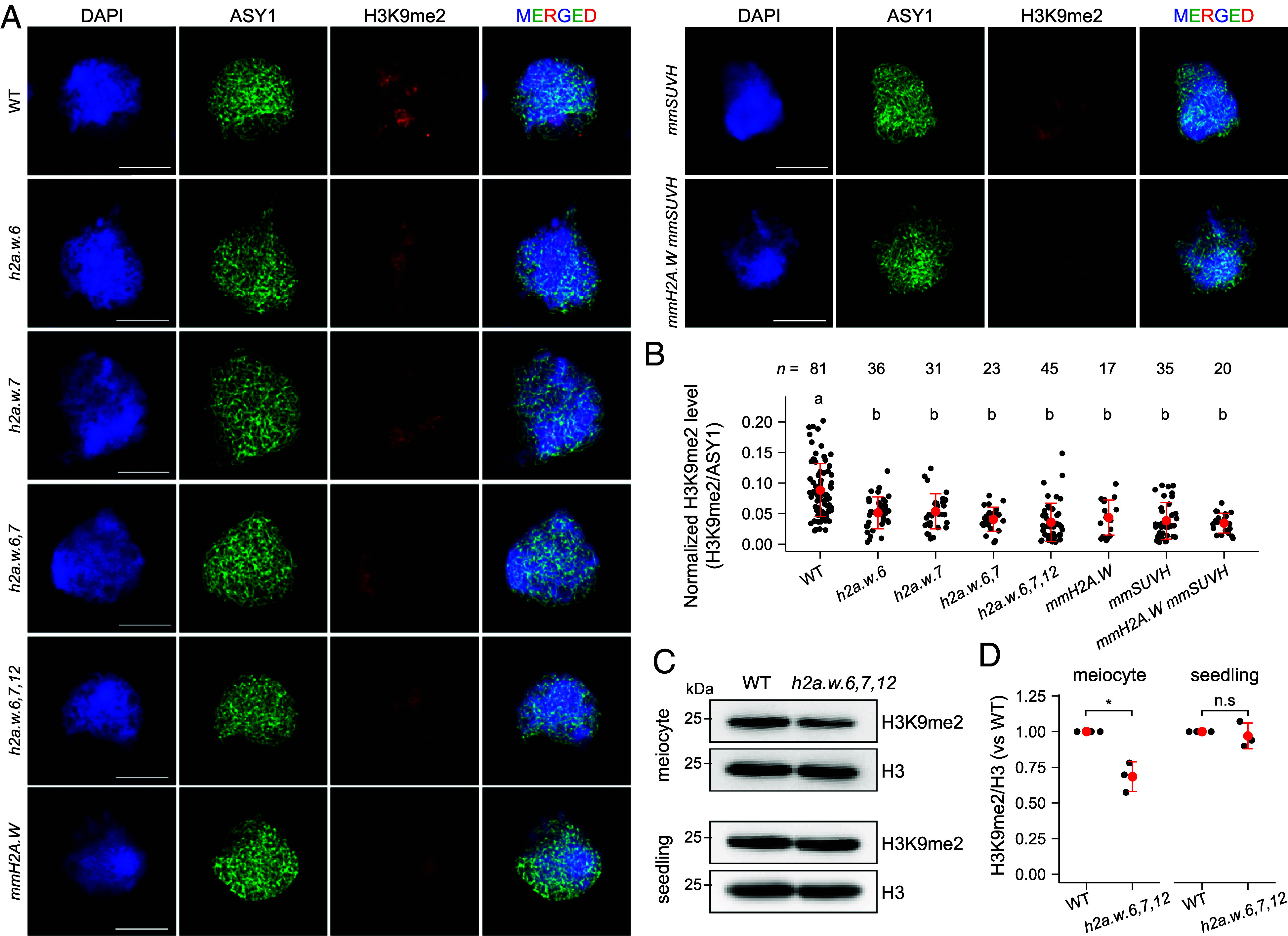
Reduced H3K9me2 levels in *mmH2A.W* lines and *h2a.w* mutants. (*A*) Male meiocyte images from WT (Col-0), *h2a.w.6-3*, *h2a.w.7-3*, *h2a.w.6*,*7*, *h2a.w.6,7,12* (*h2a.w-2*), *meiMIGS-H2A.W.6/7/12* (*mmH2A.W*), *meiMIGS-SUVH4/5/6* (*mmSUVH*), and *mmH2A.W mmSUVH* immunostained for ASY1 (green) and H3K9me2 (red), with DNA stained by DAPI (blue) at zygotene. (Scale bars, 10 µm.) (*B*) Quantification of H3K9me2 immunostaining in (*A*). Black dots represent H3K9me2 levels normalized to ASY1 per cell. Data are means ± SD, with *n* indicating the number of meiocytes. Significant differences were determined by one-way ANOVA with Games–Howell tests (*P* < 0.05). (*C* and *D*) Immunoblot (*C*) and quantification (*D*) of H3K9me2 in male meiocytes and 10-d-old seedlings from WT and *h2a.w.6,7,12*. H3 served as a loading control. Data represent mean ± SD from three independent experiments. Asterisks indicate significant differences (*P < 0.05; Welch’s *t* test); n.s., not significant.

H2A.W.6, H2A.W.7, and H3K9me2 are tightly associated with heterochromatin organization (33, 37, 39), and we also observed that H3K9me2 is densely deposited at meiotic heterochromatin during prophase I as previously described (30) (*SI Appendix*, Fig. S10). We therefore performed immunostaining for H3K9me2 and ASYNAPTIC 1 (ASY1), a component of the meiotic chromosome axis, at the zygotene stage of prophase I in male meiocytes to investigate whether the *h2a.w* mutants, *mmH2A.W*, *mmSUHV*, and *mmH2A.W mmSUHV* lines with more heterochromatic crossovers might exhibit altered H3K9me2 levels during meiosis (Fig. 5 *A* and *B*). By normalizing the immunostaining H3K9me2 level to that of ASY1, we found a decrease in H3K9me2 intensity per cell in the *h2a.w.6*, *h2a.w.7*, *h2a.w.6*,*7*, and *h2a.w.6,7,12* mutants and *mmH2A.W* lines as well as in the *mmSUVH* and *mmH2A.W mmSUVH* lines compared to the WT Col-0 (Fig. 5 *A* and *B* and Dataset S7). To further examine the effect of *h2a.w.6,7,12* on H3K9me2 deposition during meiosis, we purified male meiocytes from ~0.3 to 0.5 mm flower buds to perform immunoblot analysis. We observed that H3K9me2 levels were reduced in *h2a.w.6,7,12* meiocytes compared to the WT, whereas no changes were detected in *h2a.w.6,7,12* seedlings, consistent with previous report (Fig. 5 *C* and *D* and Dataset S8) (48). The H3K9me2 immunostaining and immunoblot results, combined with the GBS and MNase-seq analyses in *h2a.w* mutants, suggest that loss of H2A.W.6 and/or H2A.W.7 leads to heterochromatin decondensation and diminished H3K9me2 levels in heterochromatic nucleosomes during meiosis, thereby increasing the number of heterochromatic crossovers.

## Discussion

We show here that meiosis-specific knockdown and knockout of genes encoding H2A.W variants increase the number of crossovers at heterochromatic pericentromeres in *A. thaliana* (Figs. 1–3). Thus, we suggest that genetic loss or silencing of *H2A.W* is a potential strategy to increase the number of heterochromatic crossovers in other plants with a comparable heterochromatin organization. Genetic inactivation of the non-CG methylation/H3K9me2 pathway was previously shown to increase the number of pericentromeric crossovers in *A. thaliana* (30). Our analysis of crossovers using transgenic *meiMIGS* plants confirmed that meiotic silencing of *SUVH4/5/6* or *CMT2*/*3* can similarly increase the number of heterochromatic crossovers in *A. thaliana* (Figs. 1 and 2), as previously shown in the *suvh4*,*5*,*6* and *cmt3* mutants (30). In particular, an increase in heterochromatic crossovers mediated by a meiMIGS or meiosis-specific RNA interference system might be useful for recombination or mapping of favorable quantitative trait loci in crop species, since genetic inactivation of the non-CG methylation/H3K9me2 pathway results in death in maize or developmental defects in tomato (60–62).

H2A.Ws are deposited over constitutive heterochromatin at positions that overlap with H3K9me2 in pericentromeric regions (37, 47). However, the complete loss of three H2A.Ws has little effect on TE transcription or H3K9m2 levels in seedlings (48). Nevertheless, we observed that *h2a.w.6*, *h2a.w.7*, *h2a.w.6*,*7*, and *h2a.w.6*,*7*,*12* mutations, as well as meiotic knockdown of *H2A.W.6/7/12*, led to a similar increase in the number of crossovers in heterochromatic pericentromeric regions (Figs. 2, 3 and Tables 1 and 2). This finding suggests that the mutations and knockdown of three *H2A.W* genes may decrease heterochromatin compaction as reported in plant gametes (37), thereby increasing the formation of heterochromatic crossovers. Indeed, our genome-wide nucleosome density profiling by MNase-seq showed that heterochromatin accessibility had increased in the *h2a.w.6*, *h2a.w.7*, *h2a.w.6*,*7*, and *h2a.w.6*,*7*,*12* mutants (Fig. 4), strongly supporting the idea that H2A.Ws promote heterochromatin compaction via their DNA-binding KSPK motif (37, 38, 47). High-resolution profiling of chromatin accessibility by MNase-seq has been shown to correlate with the sequencing enrichment levels of SPO11-1 oligonucleotides covalently attached to SPO11-1, which are indicative of meiotic DSBs in *A. thaliana* (30, 35). Based on this correlation, it is plausible that *h2a.w.6*, *h2a.w.7*, *h2a.w.6*,*7*, and *h2a.w.6*,*7*,*12* mutants exhibit an increased number of meiotic DSBs in heterochromatic regions, similar to *suvh4,5,6* (30). The loss of H2A.W.6 and/or H2A.W.7 may result in a subset of these additional DSBs being repaired into crossovers, potentially by promoting downstream DSB repair processes, including DSB end resection, homology search, and crossover resolution. However, the sequencing of SPO11-1 oligonucleotides, the quantification and mapping of DSB foci using immunostaining and superresolution microscopy, and the mechanisms by which additional pericentromeric DSBs are repaired to crossovers in *h2a.w* mutants remain to be explored. Importantly, through immuno-cytological quantification analysis of H3K9me2 levels in male meiotic cells, we observed lower H3K9me2 levels in the genotypes with more pericentromeric crossovers (*h2a.w* mutants and *meiMIGS-H2A.W.6/7/12*, as well as in *meiMIGS-SUVH4/5/6* and *meiMIGS-H2A.W.6/7/12 meiMIGS-SUVH4/5/6*), suggesting that H2A.W-driven heterochromatin compaction is required to maintain H3K9me2 during meiosis prophase I (Fig. 5). Consistently, immunoblot analysis showed decreased H3K9me2 levels in *h2a.w.6,7,12* male meiocytes compared to WT (Fig. 5 *C* and *D*). However, as H3K9me2 ChIP-seq data showed no significant change in H3K9me2 levels in *h2a.w.6,7,12* seedlings compared to WT controls (48), it is possible that the decrease in H3K9me2 levels in *h2a.w.6,7,12* may be specific to meiosis prophase I.

Genetic disruption of *H2A.W*s in combination with loss of linker histone H1, non-CG methylation, or H3K9me2 results in synergistic transcriptional upregulation of TEs (49), suggesting that H2A.W cooperates with the histone H1 and H3K9me2/non-CG pathways in TE silencing and heterochromatin organization. The highest transcriptional upregulation of TEs is observed in *h2a.w.6,7,12 suvh4*,*5*,*6* among combination mutations of *h2a.w.6,7,12* with *h1*, *cmt2,3*, or *suvh4*,*5*,*6* (49). However, we observed that *meiMIGS-H2A.W.6/7/12 meiMIGS-SUVH4/5/6* did not show an additive or synergistic increase in the number of heterochromatic crossovers compared to *meiMIGS-H2A.W.6/7/12* and *meiMIGS-SUVH4/5/6* (Fig. 2). This result indicates that the extent of transcriptional reactivation of TEs does not correlate with an increased number of heterochromatic crossovers in epigenetic mutants, which is consistent with observations in the *met1* and *ddm1* mutants where the number of pericentromeric crossovers instead decreased, even with ectopic transcriptional activation of TEs and increased heterochromatin accessibility (35, 45, 46). Therefore, to maximize heterochromatic crossovers and break linkage drag, other heterochromatin-organizing factors such as H3K27me1 and histones H1 or H3.1 should be investigated through a combination of genetic inactivation or knockdowns with *H2A.W* and H3K9me2/non-CG methylation, in addition to pro- and anti-crossover factor genes in the class I and II pathways.

## Materials and Methods

Plasmid constructs for *meiMIGS* transgenic plants and CRISPR/Cas9 knockout mutants were generated using the Golden Gate system, with primers listed in *SI Appendix*, Table S12. Crossover frequencies in the *CTL3.9* and *CEN3* reporter lines were measured by analyzing fluorescent seeds and pollen, respectively, using automated image analysis pipelines. GBS libraries were constructed using gDNA extracted from Col-0 × L*er* F_2_ plants and sequenced to identify crossover sites. MNase-seq libraries were prepared from flower buds and seedlings via nuclei isolation, MNase digestion, and nucleosomal DNA extraction, followed by library construction and 2 × 100-bp paired-end sequencing. Anthers dissected from buds were used for H3K4me2 immunostaining. Detailed materials, growth conditions, and methods are provided in *SI Appendix, Materials and Methods*.

## Supplementary Material

Appendix 01 (PDF)

Dataset S01 (XLSX)

Dataset S02 (XLSX)

Dataset S03 (XLSX)

Dataset S04 (PDF)

Dataset S05 (XLSX)

Dataset S06 (XLSX)

Dataset S07 (XLSX)

Dataset S08 (PDF)

## Data Availability

Sequencing data from GBS and MNase-seq are available in the ArrayExpress database at EMBL-EBI (http://www.ebi.ac.uk/arrayexpress) under the accession numbers provided in *SI Appendix,* Table S10. Images of H3K9me2 and ASY1 immunostaining are accessible via a public link (https://doi.org/10.6084/m9.figshare.c.7559811) (63). All other data are included in the manuscript and/or supporting information.
