# Supplementary material for: The histone variant H2A.W restricts heterochromatic crossovers in Arabidopsis
Source: Proc Natl Acad Sci U S A. 2025 Apr 4;122(14):e2413698122. doi: 10.1073/pnas.2413698122 (PMC12002335; doi:10.1073/pnas.2413698122)
Supplement: Supplementary file 1 — Appendix 01 (PDF) [file pnas.2413698122.sapp.pdf]

PNAS

Supporting Information for
The histone variant H2A.W restricts heterochromatic crossovers in *Arabidopsis*

Namil Son[1†], Heejin Kim[1†], Jaeil Kim[1], Jihye Park[1], Dohwan Byun[1], Sang-jun Park[1], Hyein Kim[1], Yeong Mi Park[1], Pierre Bourguet[2], Frédéric Berger[2], and Kyuha Choi[1]*

*Kyuha Choi
Email: kyuha@postech.ac.kr

This PDF file includes:

Materials and Methods
Figures S1 to S10
Tables S1 to S12
Legends for Datasets S1 to S8
SI References

Other supporting materials for this manuscript include the following:

Datasets S1 to S8

Materials and Methods

Plant Materials

The *Arabidopsis thaliana* accession Columbia-0 (Col-0) accession was used as the wild type. Plants were grown in controlled growth rooms under the conditions of 20°C, 50–60% humidity, and a 16-h light/8-h dark photoperiod. Fluorescence-tagged lines for seed scoring (*CTL3.9*) and pollen tetrad analysis (*CEN3*) were used as described (1–3). The *h2a.w-2* mutant was used as described (4). The Landsberg *erecta*-0 (L*er*) accession was used to generate Col × L*er* WT hybrids for genotyping-by-sequencing.

Generation of meiMIGS Transgenic Plants

To generate meiosis-specific microRNA-induced gene silencing (meiMIGS) vectors, a 1.5-kb DNA fragment containing the *DMC1* promoter, the 5′ UTR, two introns, and the third exon was PCR amplified from Col-0 genomic DNA using primers DMC1-1p_1.5kb-Lv0-GGAG-F and DMC1-1p_1.5kb-Lv0-CATT-R and cloned into the universal level 0 (Lv0) vector (pAGM9121) using the Golden Gate system (5). Level 0 full-length coding sequence modules for target genes were PCR amplified from Col-0 cDNA using forward primers containing the miR173 target sequence (miR173-ts) and reverse primers, and then cloned into the universal level 0 vector pAGM9121. For meiMIGS two- or three-gene cassettes for silencing, the *DMC1* promoter and each miR173-ts–coding sequence were assembled into level 1 (Lv1) position 2 (pICH47742), position 3 (pICH47751) and/or position 4 (pICH47761) vectors, with the terminator from the *Nopaline synthase gene* (*NOS*) (pICH41421). The Lv1 vectors containing each meiMIGS cassette were individually assembled into a level 2 (Lv2) binary vector (pAGM4723) with the Lv vector pICSL11017 containing the herbicide resistance gene *BAR* and the linker vector pICH41744 based on the Golden Gate cloning system. The final Lv2 binary vectors were introduced into *Agrobacterium* (*Agrobacterium tumefaciens*) strain GV3101-pSOUP via electroporation. Positive Agrobacterium colonies were then used for transformation of *A. thaliana* plants by floral dipping. All primers used for cloning are listed in *SI Appendix*, Table S12.

High-throughput Measurements of Crossover Frequency

The genetic distances (in cM) of the fluorescent seed crossover reporter *CTL3.9* (Columbia *Traffic Line 3.9*) were measured using epifluorescence microscopy imaging and the CellProfiler image analysis pipeline as described (6, 7). The CellProfiler pipeline allows

the quantification of the number of green-only fluorescent seeds (N_{Green}), red-only fluorescent seeds (N_{Red}), and total seeds (N_{Total}). The crossover frequency (in cM) is calculated using the following formula (6–8): cM = 100 × (1 − [1 − 2(N_{Green} + N_{Red})/N_{Total}]$^{1/2}$). Two-tailed Welch's *t*-tests or one-way analysis of variance (ANOVA) followed by Turkey's tests were used to examine the significance of differences in crossover frequencies between genotypes by calculating *P*-values.

Pollen from fluorescence-tagged lines (FTLs) produce a tetrad with the four pollen products of male meiosis that remain attached in the *qrt-1* background. Pollen tetrad analysis of the FTL *CEN3* interval expressing the fluorescent reporters *eYFP* and *dsRed* under the post-meiotic *LAT52* promoter was performed to measure crossover frequency (in cM) using DeepTetrad, a deep learning–based image analysis pipeline as described (3). The FTL *CEN3* generates three types of tetrads: parental ditype (PD), tetra type (T), and non-parental ditype (NPD). DeepTetrad allows quantification of the number of tetrad types and calculation of the crossover frequency (in cM) using the Perkin's equation:

$$cM = \frac{0.5T + 3NPD}{\left(PD + T + NPD\right)} * 100$$

Quantitative RT-PCR Analysis

Total RNA was extracted from the unopen flower buds of 6-week-old plants using Trizol reagent (Invitrogen) according to the manufacturer's instructions. Two micrograms of total RNA was used for first-strand cDNA synthesis using a reverse transcription (RT) kit (Enzynomics, EZ405S). Quantitative PCR (qPCR) was performed using a CFX real-time PCR detection system (Bio-Rad) and qPCR premix kit (Enzynomics, RT500). Expression levels were normalized to the expression of the meiosis-specific gene *DMC1*. Four biological replicates were performed for each sample (*SI Appendix*, Dataset S1).

Generation of *h2a.w* Knockout Alleles Using CRISPR/Cas9

To generate *h2a.w.6 h2a.w.7 h2a.w.12* knockout plants, a 20-bp DNA fragment for a single guide RNA (sgRNA) targeting *H2A.W.6*, *H2A.W.7*, or *H2A.W.12* was assembled into a Lv0 vector (pAGM9121), along with the *U6* promoter. To induce a large DNA deletion, a pair of sgRNAs was designed for each gene, and six sgRNAs were assembled into the Lv1 vector pICH47751. The resulting Lv1 vector was assembled into a Lv2 binary vector with the Lv1 vector (pICSL11017) containing the *BAR* gene and an *RPS5A* promoter-driven CRISPR/Cas9 expression Lv1 vector (KEJ-L1-42). The final Lv2 binary

vector was electroporated into *Agrobacterium* strain GV3101 and transformed into the *A. thaliana* Col-0 and L*er* accessions by floral dipping. The deletion alleles for *h2a.w.6*, *h2a.w.7*, and *h2a.w.12* in the Col-0 and L*er* backgrounds were identified by PCR and gel electrophoresis using genomic DNA from T_1 plants. The deletions of each of *H2A.W.6*, *H2A.W.7*, and *H2A.W.12* and the absence of the *Cas9*/sgRNAs transgene were confirmed in the T_2 and T_3 generations by PCR and Sanger sequencing (*SI Appendix*, Dataset S4).

Genotyping-By-Sequencing for Mapping Crossovers

For genotyping-by-sequencing (GBS) library preparation (*SI Appendix*, Table S11), individual F_2 plants were grown on soil for 3 weeks, and three mature leaves per plant were used to extract genomic DNA (gDNA) using a CTAB method. GBS libraries were prepared as described previously (5, 9). Briefly, 150 ng of gDNA for each F_2 plant was quantified using a Qubit dsDNA Broad Range assay kit (Thermo Fisher) and fragmented using dsDNA Shearase (Zymo Research) in a 15-μL reaction at 37°C for 20 min, followed by an incubation at 65°C for 5 min to inactivate the enzyme. The digested DNA was end-repaired in a reaction volume of 30 μL (3 units of T4 DNA polymerase [New England Biolabs], 10 units of T4 polynucleotide kinase [Thermo Fisher Scientific], 1.25 units of Klenow fragment [New England Biolabs], and 0.4 mM dNTPs) at 20°C for 30 min. The resulting DNA fragments were purified using AMPure XP magnetic SPRI beads (Beckman-Coulter, A63881), following the procedure as described (9). The DNA was A-tailed and ligated with barcoded Illumina adapters in a reaction volume of 20 μL, as detailed in reference (9). Eight DNA libraries were pooled, washed twice with 80% (v/v) ethanol for 30 s each, and eluted in 30 μL elution buffer (10 mM Tris-HCl, pH 8.0). The 30-μL mixture was combined with 16 μL of AMPure XP magnetic SPRI beads (Beckman-Coulter) in a tube. After 5 min of incubation at room temperature, the samples were placed on a magnetic rack for 2 min, and the supernatant (42 μL) was transferred to a fresh tube and mixed with 0.23 volume (9.5 μL) of SPRI beads. After another 5 min of incubation at room temperature, the tubes were returned to the magnetic rack for 2 min. The supernatant was discarded, and the beads were washed twice with 80% (v/v) ethanol for 30 s each. The beads were then air-dried for 10 min, and the DNA was eluted in 20 μL of 10 mM Tris-HCl, pH 8.0. Twelve μL of the eluate was subjected to 12 cycles of PCR amplification in a reaction volume of 50 μL using a KAPA HiFi HotStart ReadyMix P*CR* kit (Roche) and the specified DNA oligonucleotides from reference (9). The PCR products were then purified using SPRI beads and quantified using a Bioanalyzer. The 96 barcoded

libraries were pooled and sequenced on an Illumina HiSeqX instrument (Microgen, Korea) using paired-end 150-bp sequencing (*SI Appendix*, Table S11). The TIGER pipeline was used to analyze the data and map crossover sites as described (9).

Micrococcal Nuclease (MNase) Sequencing Library Preparation

The MNase-seq library was prepared as previously described (10). Briefly, 1 g of unopened flower buds that were smaller than approximately 2 mm or ~0.3–0.5 mm flower buds or 10-day-old seedlings was ground to a fine powder in liquid nitrogen using a mortar and pestle. To isolate nuclei, the resulting powder was transferred to ice-cold 40 mL Honda buffer (25 mM Tris-HCl, pH 7.5, 0.44 M sucrose, 10 mM $MgCl_2$, 0.5% [v/v] Triton X-100, 10 mM β-mercaptoethanol, 2 mM spermine, 0.4 mM PMSF, 1 μg pepstatin, and 1× protein inhibitor cocktails [Roche]) and kept on ice for 30 min with gentle mixing. The lysate was filtered through two layers of Miracloth (Merck Millipore) and centrifuged at 2,000*g* at 4°C for 20 min. The pellet was resuspended in 20 mL Honda buffer and centrifuged at 2,000*g* at 4°C for 15 min. The nuclei pellet was rinsed with 1 mL of TNE buffer (10 mM Tris-HCl, pH 8.0, 10 mM NaCl, 1 mM EDTA, 1× proteinase inhibitor cocktail), resuspended in the same buffer, and then centrifuged at 2,000*g* at 4°C for 5 min. Chromatin was digested with 0.05 units of micrococcal nuclease (MNase, NEB M0247S) in reaction buffer (10 mM Tris-HCl, pH 8.0, 10 mM NaCl, 1 mM EDTA, 4 mM $CaCl_2$, 0.5 μg/μL RNaseA) at 37°C for 12 min with vortexing at 1,000 rpm in a thermomixer (Eppendorf). The reaction was stopped by addition of EDTA to a final concentration of 50 mM, vortexing, incubation on ice for 5 min, and centrifugation at 14,000*g* at 4°C for 5 min. The supernatant was incubated with proteinase K (0.1 mg/mL) at 37°C for 30 min and purified by phenol-chloroform-isoamyl alcohol (25:24:1, v/v/v) extraction, followed by ethanol precipitation, electrophoresis on a 2% (w/v) agarose gel, and gel extraction of approximately 145–150 bp DNA. Three independent biological replicates were performed for high-throughput sequencing library preparation. Approximately 10 ng of purified nucleosomal DNA was used to generate a library for 2 × 100-bp paired-end sequencing using DNBseq sequencing technology (BGI, Hong Kong) (*SI Appendix*, Table S11). As a control for MNase-seq, sequencing data from Col-0 genomic DNA was used as previously described (10).

Bioinformatics Analysis of MNase-seq Data.

FASTQ files containing the paired-end data were aligned to the TAIR10 reference genome sequence using Bowtie2 with the following settings ″--very-sensitive --no-discordant --no-

mixed -p 4 -k 10″. To obtain uniquely aligning reads, sequencing reads with the SAM optional field ″XS:i″ and MAPQ scores below 42 were removed. A Python script was used to ensure proper pairs of reads. Reads with multiple valid alignments were further filtered to retain only those with MAPQ scores of at least 10, with preference given to alignments with the highest value. In the case of multiple alignments with equal scores, the alignments with the highest MAPQ score were retained, or randomly assigned. The resulting uniquely and multiply aligned reads were then deduplicated using SAMtools, combined, and used for downstream analysis. Coverage values from these reads were calculated using Rsamtools and normalized to the sum of coverage per library. To normalize MNase-seq data, a Col-0 genomic DNA sequencing library of paired-end 150-bp reads was used as described (10). These normalized data were aligned and processed as for the MNase-seq libraries and used to calculate $\log_2$(MNase/gDNA) values. For H2A.W chromatin immunoprecipitation sequencing (ChIP-seq) data, the same procedure as for MNase-seq was used and the data were used to calculate $\log_2$(ChIP/input) values using ChIP input and ChIP-seq data (*SI Appendix*, Table S11 and see below).

Bioinformatics Analysis of H2A.W ChIP-seq Data

For H2A.W ChIP-seq analysis, publicly available ChIP-seq data from H2A.W.6 (SRR5298545), H2A.W.7 (SRR5298546), and input control (SRR5298544) were used (*SI Appendix*, Table S11) (11). Reads were trimmed using Cutadapt (ver. 4.4) to remove adapter sequences, and aligned to the TAIR10 reference genome using Bowtie2 (ver. 2.5.1) with the following settings: ″--very-sensitive --no-mixed″. Only primary alignments with MAPQ ≥ 10 were retained using SAMtools view (ver. 1.17) with the following settings: ″-F 2308 -q 10″ to obtain reliable alignments. Duplicate reads were removed using Picard MarkDuplicates, and the resulting BAM files were used to calculate coverage for each position in the genome using Deeptools bamCoverage (ver. 3.5.4). These data were used to calculate $\log_2$(ChIP/Input) using Deeptools bigwigCompare. To define ChIP-seq peaks, MACS2 callpeak was used with the following settings: ″--keep-dup all --braod″. The defined ChIP-seq peaks were divided into those on chromosome arms and those on pericentromeres. H2A.W.6 and H2A.W.7 peaks that overlapped were merged and defined as shared peaks, while the remaining peaks were defined as H2A.W.6-specific or H2A.W.7-specific peaks.

Immunocytological Analysis

Immunostaining for H3K9me2 was performed on fresh flower buds. Anthers were dissected from 0.4-mm buds at floral stage 9, and transferred into 10 μL of enzyme digestion solution (0.4% [w/v] cytohelicase, 1.5% [w/v] sucrose, 1% [w/v] polyvinylpyrrolidone). The anthers were tapped with a brass rod and incubated in a moist box at 37°C for 3 min. Then, 10 μL of 1% (v/v) Lipsol was added and mixed with a needle for 1 min, followed by the addition of 20 μL 4% (w/v) paraformaldehyde. The slides were dried in a fume hood for 2 h. The slides were then incubated with primary antibodies at 4°C for 1 day. The slides were washed three times in phosphate buffered saline Triton X-100 (PBST, 1× PBS with 0.1% [v/v] Triton X-100) for 5 min each time. Secondary antibodies were added and incubated at 37°C for 1 h. The above washing step was repeated, and then 10 μL of DAPI/Vectashield was added before placing a coverslip on top of the slide. The following antibodies were used for immunostaining: α-ASY1 (rabbit, 1:500 dilution) (12), and α-H3K9me2 (mouse, 1:200 dilution; Abcam ab1220). Imaging was conducted using a Zeiss LSM 800 confocal laser scanning microscope with a ×63/1.42 oil objective lens. All slides and images were prepared and captured under identical conditions. Cell stages from late leptotene to early zygotene were determined based on the ASY1 and DAPI staining patterns. Individual nuclei were acquired as 0.14-μm *z*-stacks, and a sufficient number of stacks were captured to ensure that the nuclei were out of focus at both ends. Image processing and analysis were performed using Zeiss Zen 2.6 (blue edition) software. Maximum intensity projections along the *z*-axis were generated, and the total signal intensity within the nuclei was measured. H3K9me2 intensity was normalized to ASY1 signal intensity. Data were compiled from duplicate experiments.

Immunoblot analysis of H3K9me2 and H3

Approximately 100 mg of 10-day-old plants were used for nuclear protein extraction. The plant tissues were frozen in liquid nitrogen and ground with two steel beads using a laboratory mixer mill (MM400, Retsch). The powdered tissue was mixed with 1.5 mL of Honda buffer (25 mM Tris-HCl, pH 7.5, 0.44 M sucrose, 10 mM $MgCl_2$, 0.5% Triton X-100 10 mM β-mercaptoethanol, 2 mM spermine, protease inhibitor cocktail (Roche)) and incubated for 10 minutes at 4°C with gentle agitation. The suspension was filtered through Miracloth, followed by washing the Miracloth with 500 μL of Honda buffer. Nuclei were pelleted by centrifugation at 2,500 × g for 10 minutes at 4°C. The pellet was washed with 1.5 mL of Honda buffer and centrifuged again. The nuclear protein was extracted using

1× Laemmli buffer. For meiocytes, ~0.3–0.5 mm anthers were dissected, and ~1,500 male meiocytes were collected per genotype by squeezing between a glass slide and coverslip as described (13). The collected meiocytes were mixed with 1 mL of Honda buffer and incubated at 4°C for 1 hour with gentle agitation. Nuclei were isolated using the same procedure as for seedlings, but centrifugation was performed for 30 minutes at each step. The final nuclear pellet was suspended in 1× Laemmli buffer. Extracted nuclear proteins were separated by SDS–PAGE using 15% polyacrylamide gels and transferred to PVDF membranes (Immobilon-P, Merck Millipore). H3K9me2 and H3 on the membranes were detected using anti-H3K9me2 (Abcam, ab1220) and anti-H3 (Abcam, ab1791) antibodies, respectively.

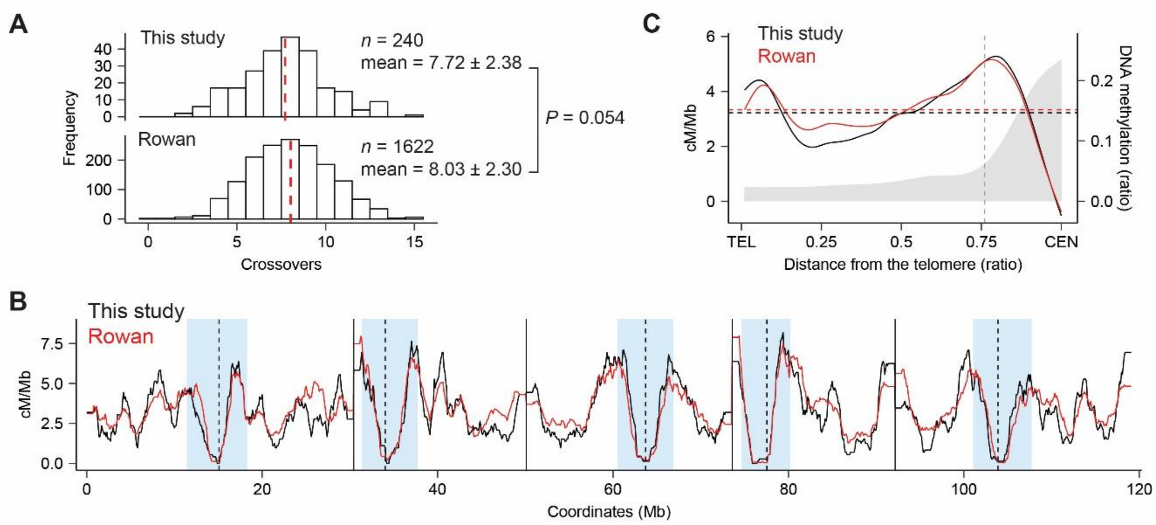

Figure S1. Comparison of WT Col-0 × L*er* F_2 GBS libraries constructed in this study with publicly available libraries.

(**A**) Histogram of crossover frequency of WT Col-0 × L*er* F_2 individuals measured from GBS libraries constructed in this study (n = 240) and publicly available GBS libraries (n = 1622) (14). Crossover frequency is shown as mean ± SD. Vertical red dotted lines indicate the mean number of crossovers per F_2 individual. Significance and *P*-value between two GBS datasets were determined using Welch′s *t*-test. (**B**) The genome-wide crossover frequency (cM/Mb) of the two WT GBS datasets. Pericentromeres are shaded blue. Vertical dotted lines and solid lines indicate centromeric assembly gaps and telomeric ends, respectively. (**C**) As for (**B**), but showing crossover frequency normalized along chromosome arms from the telomere (TEL) to the centromere (CEN). Horizontal dashed bars indicate mean crossover frequency (this study = 3.23 cM/Mb, Rowan = 3.37 cM/Mb).

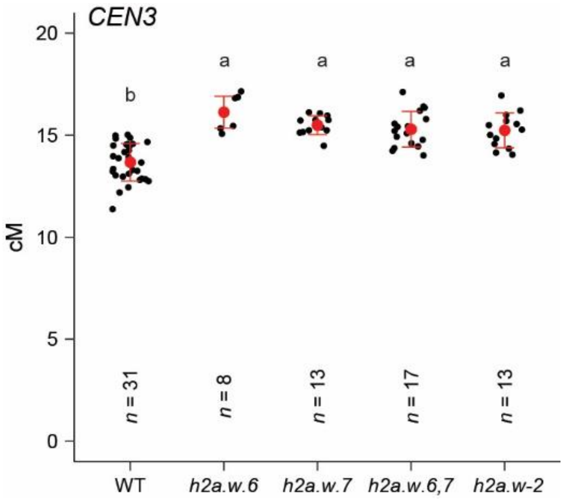

Figure S2. *CEN3* crossover frequency of WT, *h2a.w.6, h2a.w.7, h2a.w.6,7,* and *h2a.w-2.*

Crossover frequencies (cM) of *CEN3* interval in WT (Col-0), *h2a.w.6-2*, *h2a.w.7-1*, *h2a.w.6,7* (*h2a.w.6-2 h2a.w.7-1*), and *h2a.w-2* (*h2a.w.6-2 h2a.w.7-1 h2a.w.12-1*) were measured using DeepTetrad, which allows high-throughput pollen tetrad analysis. Approximately 500–1,000 pollen tetrads per independent plant were analyzed for each data point. Data are shown as mean ± SD (*SI Appendix*, Table S9). Different lowercase letters represent significant differences between the genotypes by the one-way ANOVA followed by the Tukey HSD test for multiple comparisons ($P < 0.05$). *n* indicates the number of independent plants.

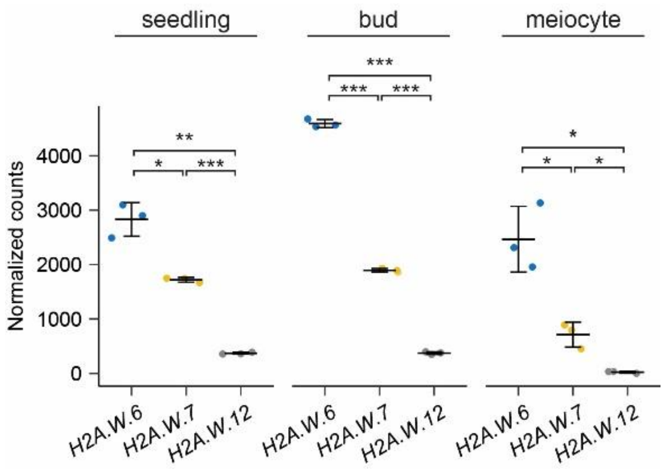

Figure S3. RNA expression levels of *H2A.W* paralogs in seedling, bud, and meiocyte.

Plot showing normalized RNA-seq read counts for *H2A.W.6*, *H2A.W.7*, and *H2A.W.12* in seedling, unopen floral bud (bud), and meiocyte (*SI Appendix*, Table S11). Data are shown as mean ± SD of three biological replicates. Asterisks indicate significant differences (**P* < 0.05, ***P* < 0.01, ****P* < 0.001; two-sided Welch's *t*-test).

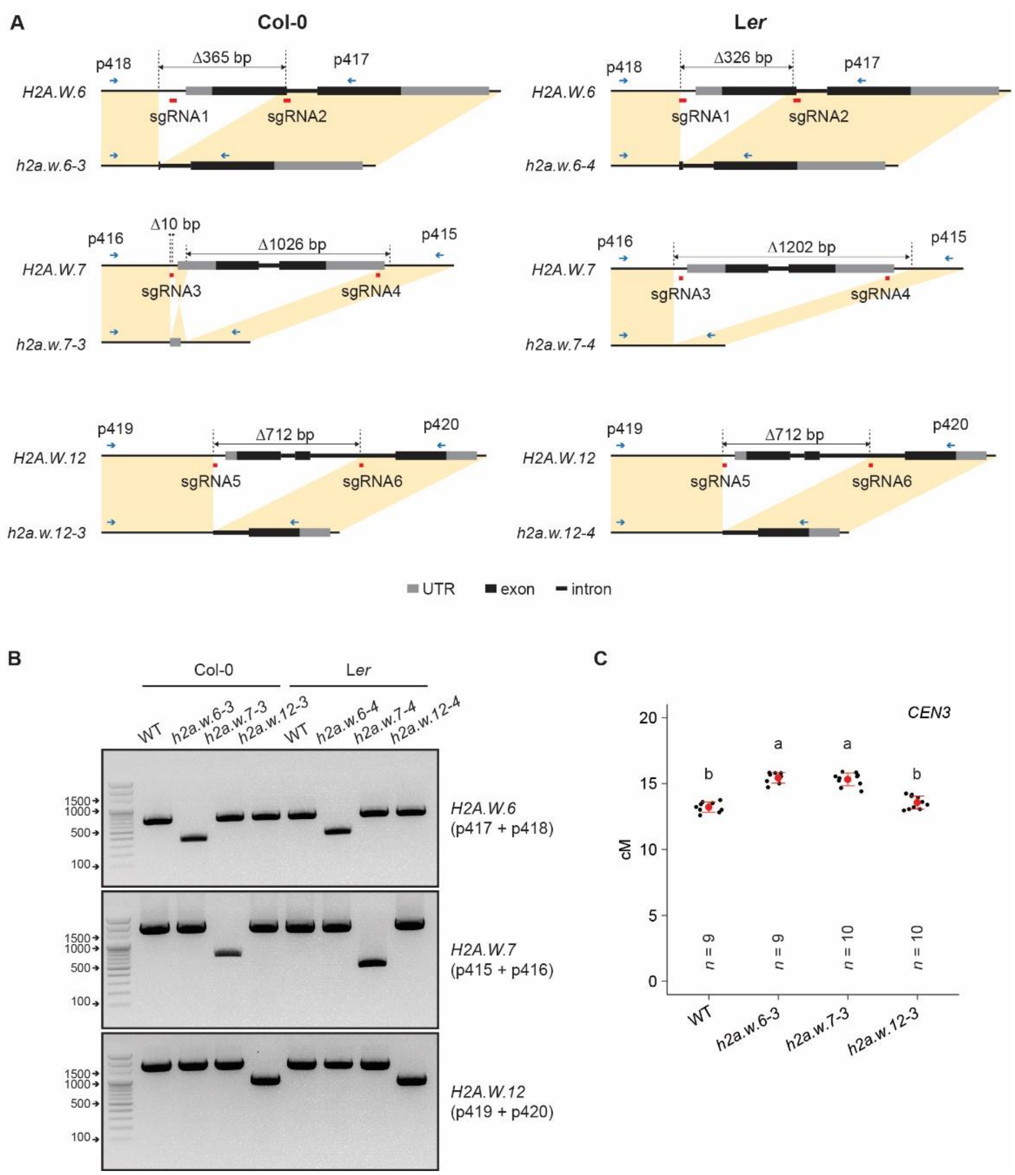

Figure S4. Generation of deletion alleles of *H2A.W* genes by CRISPR/Cas9-mediated mutagenesis.

(**A**) Diagrams of *H2A.W* genes and *h2a.w* mutant alleles in Col-0 (*left*) and L*er* (*right*) backgrounds. The single guide RNA (sgRNA) targets for CRISPR/Cas9-mediated deletion are marked with red bars, and the diagrams of the resulting deletion alleles are shown below the diagrams of the WT gene model. Sequence matches between WT and deletion alleles are connected by yellow shaded regions, and the length of the deletion sequences is marked. Blue arrows indicate the positions of the genotyping primers. (**B**) Agarose gel electrophoresis showing the genotyping result of *h2a.w* deletion mutants. Sanger sequences of the deletion alleles are available in *SI Appendix*, Dataset 3. (**C**) Crossover frequencies (cM) of *CEN3* interval in WT (Col-0), *h2a.w.6-3*, *h2a.w.7-3*, and *h2a.w.12-3.* Data are shown as mean ± SD (*SI Appendix*, Dataset S5). Different lowercase letters represent significant differences between the genotypes by the one-way ANOVA followed by the Tukey HSD test for multiple comparisons ($P < 0.05$). *n* indicates the number of independent plants.

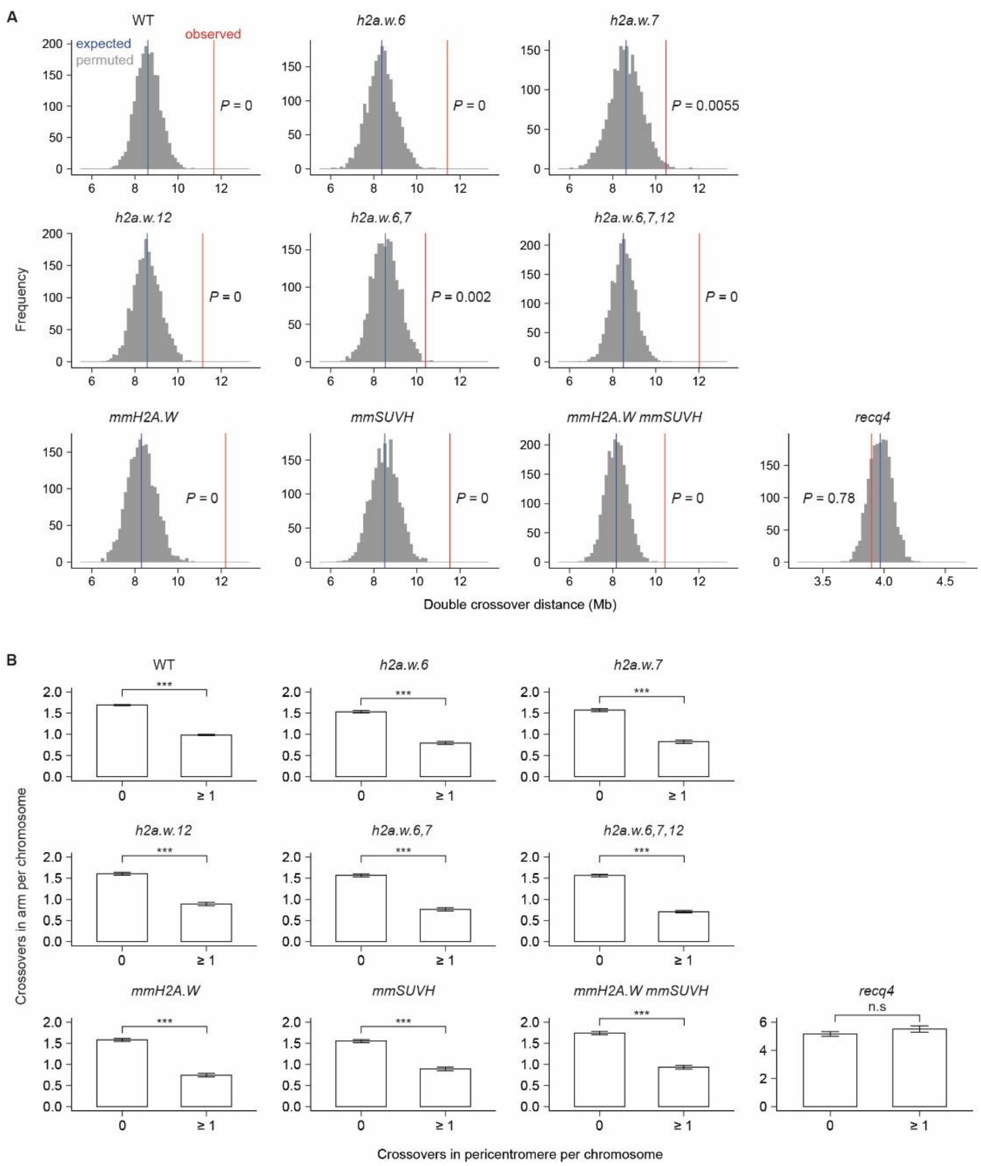

Figure S5. Analysis of double crossover distances and crossover patterning in WT, *h2a.w* mutants, *meiMIGS* lines, and *recq4a recq4b* (*recq4*) mutants.

(**A**) The mean of double crossover distances observed in each genotype are indicated by the red vertical line (observed), compared to 2,000 matched sets of randomly generated distances (permuted, gray). The mean distance of the random sets is indicated by the blue vertical line. Significance and *P*-value were determined using the permutation test. (**B**) Inhibitory effects of pericentromeric crossovers on crossover formation within the same chromosome arms in WT and *h2a.w* mutants, *meiMIGS* lines, and *recq4a recq4b* (*recq4*). Chromosomes were categorized based on whether they had zero (0) or one or more (≥ 1) crossovers in the pericentromeric regions. Data are presented as mean ± standard error. Statistical significance is indicated by asterisks (****P* < 0.0001; Wilcoxon test) or n.s. (not significant).

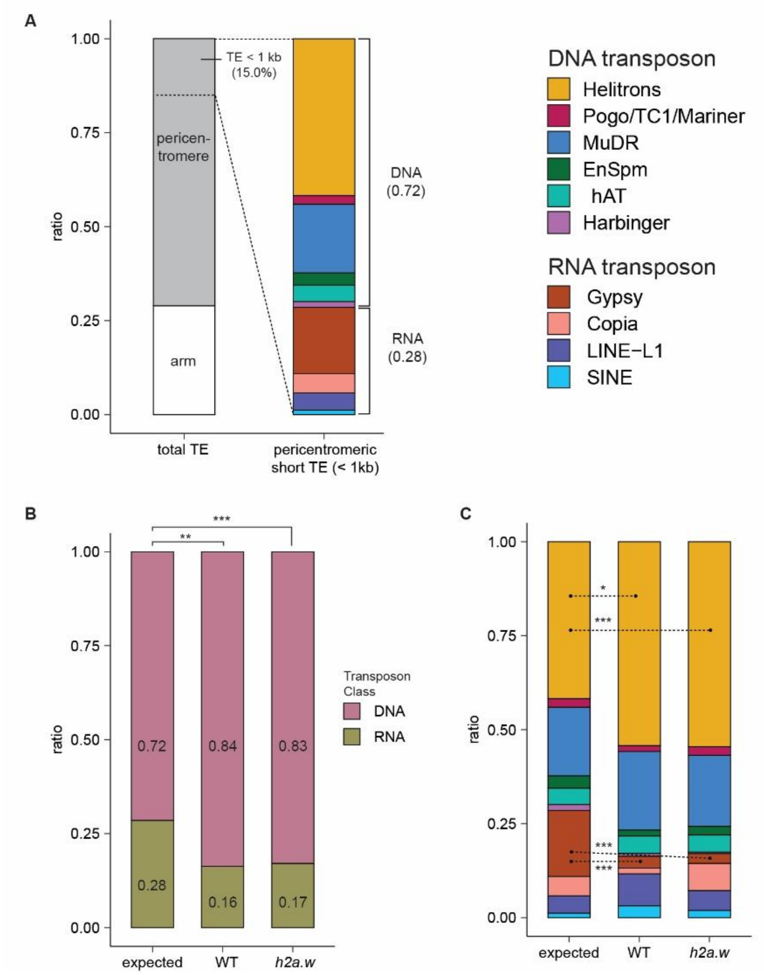

Figure S6. Analysis of short transposable elements (TE) associated with crossovers in pericentromeric regions.

(**A**) Bar plot comparing the ratio of total TE length in pericentromeric region versus chromosome arms, with short TEs (< 1kb) grouped by DNA and RNA TE superfamilies. (**B**) Ratio of short DNA and RNA TEs associated with pericentromeric crossovers in WT and *h2a.w* mutants (*h2a.w.6, h2a.w.7, h2a.w.6,7, h2a.w.6,7,12, mmH2A.W*), compared to the expected values based on pericentromeric TE length proportions. (**C**) As for (B), but with TEs categorized by superfamilies. For (**B**, **C**), asterisks indicate significant differences (*$P < 0.01$, **$P < 0.001$, ***$P < 0.0001$; Chi-square test). Datasets and P-values are provided in *SI Appendix*, Dataset S6.

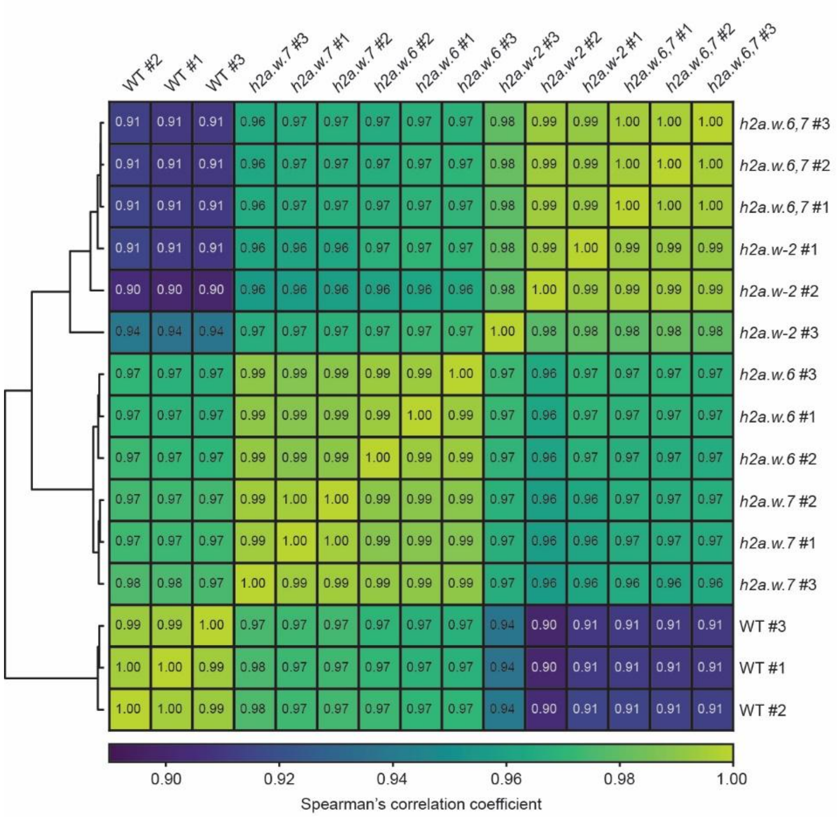

Figure S7. Correlation analysis of MNase-seq libraries used in this study.

MNase-seq data from three biological replicates (#1, #2, #3) for *h2a.w* mutants and WT Col-0 flower buds smaller than ~2 mm were hierarchically clustered based on Spearman correlation coefficients. The correlation coefficient value for each pair of data is shown in each box.

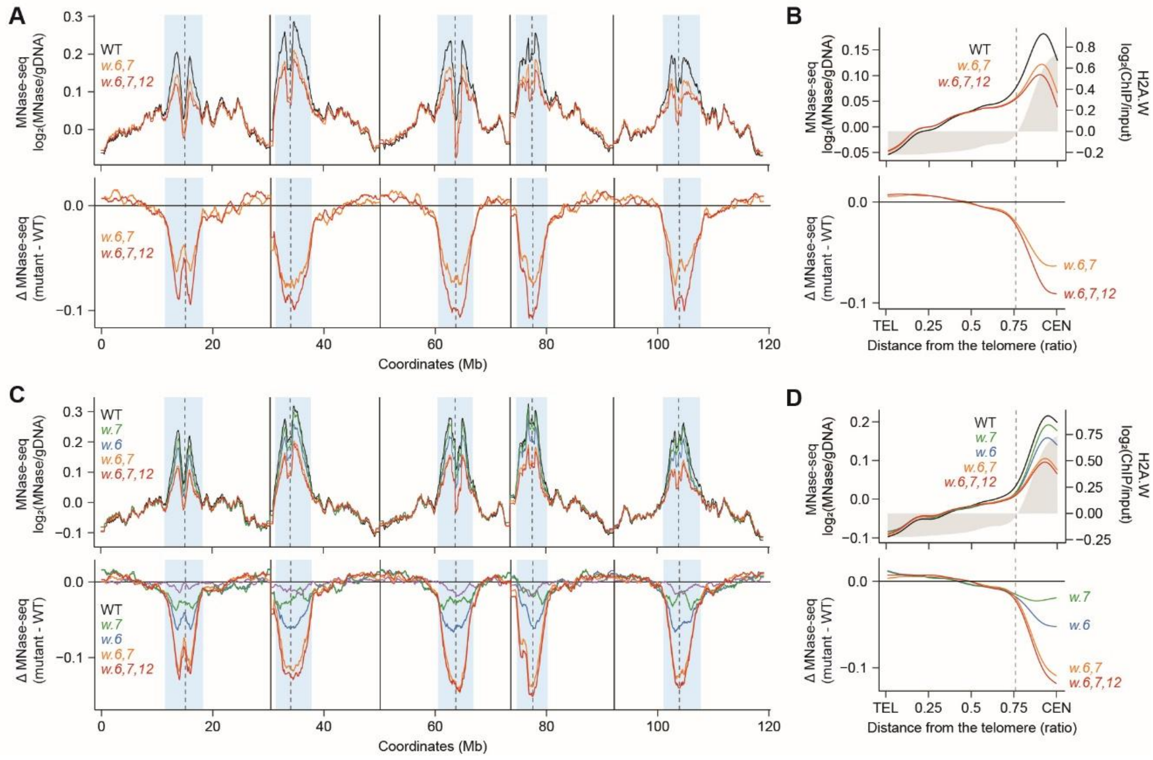

Figure S8. Genome-wide mapping of nucleosome density in 0.3–0.5 mm flower buds and 10-day-old seedlings of *h2a.w* mutants.

(**A**) Nucleosome density of the normalized MNase-seq data (as $\log_2$[MNase-seq/gDNA]) for ~0.3–0.5 mm flower buds along *A. thaliana* chromosomes was plotted on a continuous *x*-axis for WT Col-0 (black), *h2a.w.6*,*7* (*w.6,7,* orange), and *h2a.w.6,7,12* (*w.6,7,12,* red) mutants (*upper* plot), with a differential plot (mutant − WT = Δ MNase-seq) (*lower* plot). Dashed vertical lines indicate the centromere assembly gaps, and solid vertical lines represent telomere positions. The pericentromeric regions are shaded in light blue. (**B**) Normalized MNase-seq analyzed along chromosome telomere (TEL) to centromere (CEN) axes for WT and *h2a.w* mutants (*upper* plot), with the differential plot (mutant − WT = Δ MNase-seq) (*lower* plot). (**C, D**) As for (A, B), but showing plots for 10-day-old seedlings from WT Col-0 (black), *h2a.w.6-3* (*w.6,* blue), *h2a.w.7-3* (*w.7,* green), *h2a.w.6*,*7* (*w.6,7,* orange), and *h2a.w.6,7,12* (*w.6,7,12,* red).

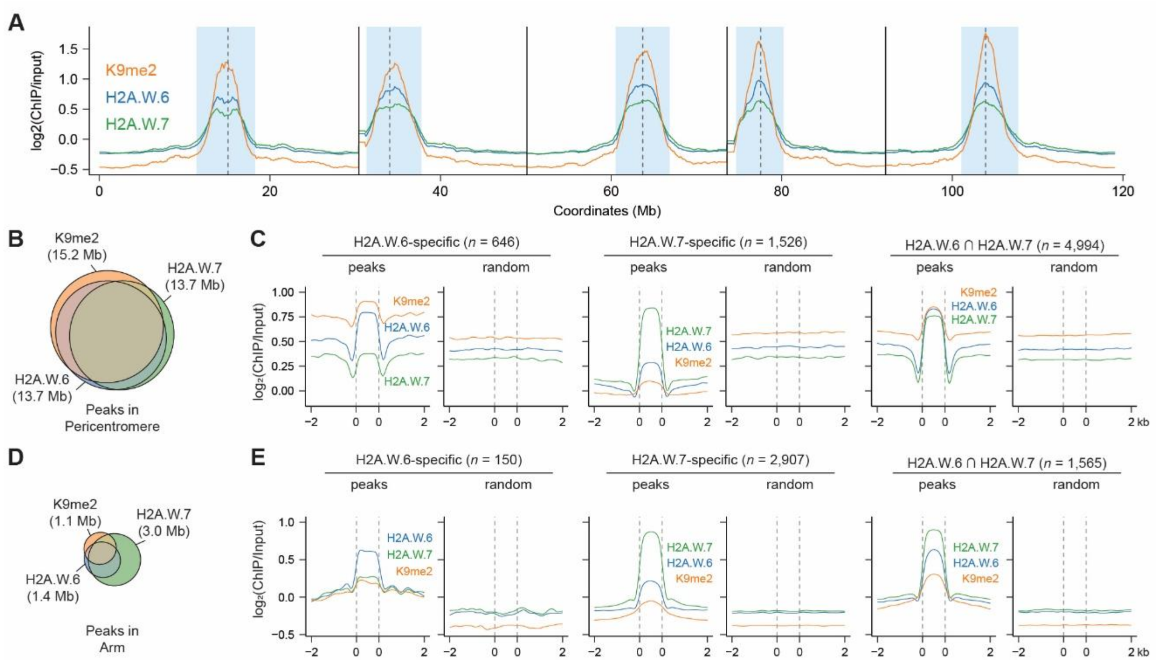

Figure S9. Distribution and overlaps of ChIP-seq peaks for H2A.W.6, H2A.W.7, and K9me2.

(**A**) Landscape of normalized ChIP-seq data for H2A.W.6, H2A.W.7, and H3K9me2 (K9me2) along *A. thaliana* chromosomes. (**B**) Venn diagram showing the overlaps of ChIP-seq peaks for H2A.W.6, H2A.W.7, and K9me2 in pericentromeric regions, with the size of each circle proportional to the total area covered by its ChIP-seq peaks. Peak coverage areas are indicated in parentheses. (**C**) Metaplot of ChIP-seq data for H2A.W.6, H2A.W.7, and K9me2 in a 4-kb window around start to end coordinates for H2A.W.6 peaks, H2A.W.7 peaks and shared H2A.W.6 and H2A.W.7 peaks in pericentromeres or for the same number of randomly selected positions. (**D, E**) As for (B, C), but analyzing chromosome arms.

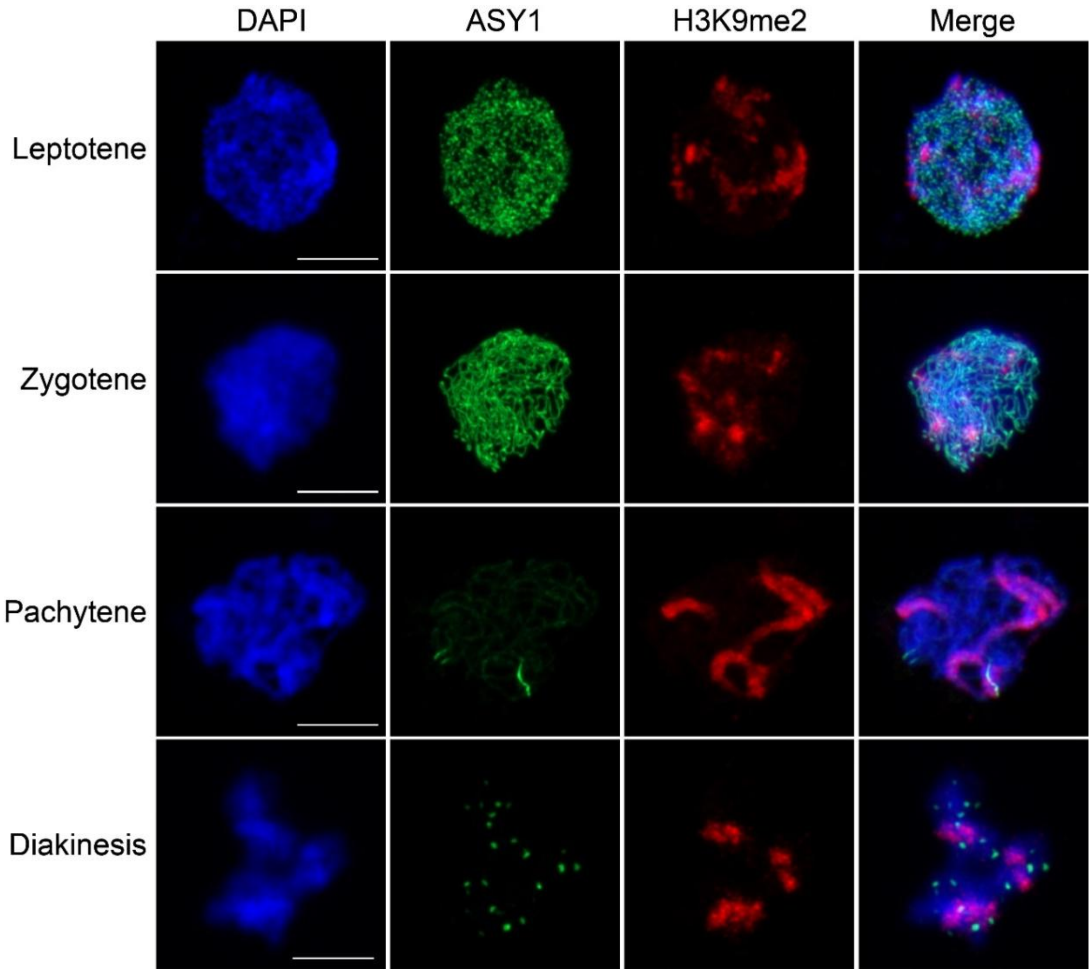

Figure S10. Co-immunostaining of H3K9me2 and ASY1 at different stages of meiotic prophase I.

Representative images of WT Col-0 male meiocytes co-immunostained for ASY1 (green) and H3K9me2 (red) and stained with DAPI (blue) are shown at different stages of meiotic prophase I. The H3K9me2 signals co-localize with heterochromatic regions that appear darkly stained with DAPI. Scale bar, 10 µm.

Table S1. *CTL3.9* crossover frequency (cM) in WT and *mmSUVH.* The crossover frequency (cM) within the *CTL3.9* interval was measured using CellProfiler as previously described (5) and calculated using the formula: cM = 100 × (1 - [1 - 2(N_{Green} + N_{Red})/N_{Total}]$^{1/2}$). The ratios R/nR and G/nG denote the ratio of red colored seed number (R) to non-red colored seed number (nR) and the ratio of green colored seed number (G) to non-green colored seed number (nG), respectively. Significance between WT and other genotypes was determined using two-sided Welch's *t*-test.

Genotype	Plant No.	R	G	None	Both	Total	cM	R/nR	G/nG	Mean	SD	*P*-value
WT (Col-0)	1	57	74	140	509	780	18.51	2.64	2.96	17.01	0.66	
WT (Col-0)	2	147	214	398	1,422	2,181	18.21	2.56	3.00			
WT (Col-0)	3	74	70	167	575	886	17.85	2.74	2.68			
WT (Col-0)	4	105	146	293	1,012	1,556	17.70	2.54	2.91			
WT (Col-0)	5	45	50	106	390	591	17.63	2.79	2.91			
WT (Col-0)	6	63	82	155	603	903	17.61	2.81	3.14			
WT (Col-0)	7	135	146	327	1,143	1,751	17.60	2.70	2.79			
WT (Col-0)	8	129	161	352	1,182	1,824	17.42	2.56	2.79			
WT (Col-0)	9	142	150	359	1,187	1,838	17.40	2.61	2.67			
WT (Col-0)	10	86	95	220	745	1146	17.29	2.64	2.75			
WT (Col-0)	11	149	173	399	1,328	2,049	17.19	2.58	2.74			
WT (Col-0)	12	130	156	326	1,209	1,821	17.18	2.78	2.99			
WT (Col-0)	13	173	172	395	1,481	2,221	16.97	2.92	2.91			
WT (Col-0)	14	102	122	247	972	1,443	16.96	2.91	3.13			
WT (Col-0)	15	27	33	76	252	388	16.89	2.56	2.77			
WT (Col-0)	16	113	142	332	1,067	1,654	16.83	2.49	2.72			
WT (Col-0)	17	118	125	293	1,042	1,578	16.81	2.78	2.84			
WT (Col-0)	18	140	141	337	1,208	1,826	16.80	2.82	2.83			
WT (Col-0)	19	133	156	337	1,254	1,880	16.78	2.81	3.00			
WT (Col-0)	20	38	42	106	337	523	16.69	2.53	2.63			
WT (Col-0)	21	145	133	346	1,201	1,825	16.61	2.81	2.72			
WT (Col-0)	22	92	84	186	797	1,159	16.56	3.29	3.17			
WT (Col-0)	23	156	147	393	1,323	2,019	16.34	2.74	2.68			
WT (Col-0)	24	165	146	405	1,362	2,078	16.29	2.77	2.65			
WT (Col-0)	25	84	109	260	842	1,295	16.22	2.51	2.76			
WT (Col-0)	26	65	83	191	668	1,007	15.97	2.68	2.93			
WT (Col-0)	27	134	153	350	1,317	1,954	15.96	2.88	3.04			
WT (Col-0)	28	129	135	352	1,187	1,803	15.91	2.70	2.75			
mmSUVH T_1	1	54	56	164	507	781	15.25	2.55	2.58	19.98	2.33	7.89×10^{-6}
mmSUVH T_1	2	138	138	244	936	1,456	21.2	2.81	2.81			
mmSUVH T_1	3	121	119	265	952	1,457	18.11	2.79	2.77			
mmSUVH T_1	4	120	95	226	735	1,176	20.35	2.66	2.4			
mmSUVH T_1	5	121	98	200	690	1,109	22.22	2.72	2.45			

mmSUVH T_1	6	162	124	289	1,058	1,633	19.39	2.95	2.62			
mmSUVH T_1	7	151	156	273	,1144	1,724	19.76	3.02	3.07			
mmSUVH T_1	8	133	103	269	907	1,412	18.41	2.8	2.51			
mmSUVH T_1	9	130	123	236	857	1,346	21	2.75	2.68			
mmSUVH T_1	10	142	129	269	998	1,538	19.53	2.86	2.74			
mmSUVH T_1	11	169	128	290	1,162	1,749	18.74	3.18	2.81			
mmSUVH T_1	12	173	161	349	1,192	1,875	19.77	2.68	2.59			
mmSUVH T_1	13	119	148	225	776	1,268	23.92	2.4	2.69			
mmSUVH T_1	14	87	126	274	1,087	1,574	14.60	2.94	3.36			
mmSUVH T_1	15	217	185	330	1,247	1,979	22.95	2.84	2.62			
mmSUVH T_1	16	148	160	280	999	1,587	21.78	2.61	2.71			
mmSUVH T_1	17	71	77	182	631	961	16.81	2.71	2.80			
mmSUVH T_1	18	165	138	279	959	1,541	22.11	2.70	2.47			
mmSUVH T_1	19	131	130	272	953	1,486	19.46	2.70	2.69			
mmSUVH T_1	20	115	103	235	763	1,216	19.91	2.60	2.47			
mmSUVH T_1	21	148	120	252	840	1,360	22.16	2.66	2.40			
mmSUVH T_1	22	160	121	233	908	1,422	22.23	3.02	2.62			
mmSUVH T_2-1	1	196	179	332	1,277	1,984	21.13	2.88	2.76	21.99	0.57	1.52×10^{-4}
mmSUVH T_2-1	2	138	113	218	810	1,279	22.06	2.86	2.59			
mmSUVH T_2-1	3	204	235	291	1,507	2,237	22.06	3.25	3.52			
mmSUVH T_2-1	4	177	250	278	1,415	2,120	22.72	3.02	3.66			
mmSUVH T_2-2	1	290	139	532	1,185	2,146	22.53	2.20	1.61	22.78	0.41	1.89×10^{-13}
mmSUVH T_2-2	2	196	99	359	802	1,456	22.88	2.18	1.62			
mmSUVH T_2-2	3	203	156	207	989	1,810	22.33	1.93	1.72			
mmSUVH T_2-2	4	244	133	429	996	1,825	23.39	2.12	1.62			
mmSUVH T_2-2	5	239	123	485	958	1,805	22.61	1.97	1.49			
mmSUVH T_2-2	6	263	108	481	947	1,799	23.35	2.05	1.42			
mmSUVH T_2-2	7	162	80	270	706	1,218	22.37	2.48	1.82			
mmSUVH T_2-3	1	173	268	362	1477	2,280	21.70	2.62	3.26	22.59	2.05	5.19×10^{-3}
mmSUVH T_2-3	2	205	145	273	1234	1,857	21.07	3.44	2.88			
mmSUVH T_2-3	3	231	154	466	825	1,676	26.48	1.70	1.40			
mmSUVH T_2-3	4	123	213	431	899	1,666	22.76	1.59	2.01			
mmSUVH T_2-3	5	169	160	271	1,155	1,755	20.94	3.07	2.99			
mmSUVH T_2-4	1	276	211	1364	364	2,215	25.15	2.85	2.46	21.81	1.78	5.25×10^{-3}
mmSUVH T_2-4	2	166	156	1092	243	1,657	21.81	3.15	3.05			
mmSUVH T_2-4	3	227	236	1602	356	2,421	21.42	3.09	3.15			
mmSUVH T_2-4	4	238	164	1411	366	2,179	20.56	3.11	2.61			
mmSUVH T_2-4	5	209	177	1403	346	2,135	20.10	3.08	2.85			

Table S2. Crossover frequency (cM) of *CEN3* in WT and *mmSUVH*. *CEN3* crossover frequency (cM) was measured by DeepTetrad using the two-color *CEN3* interval. The two-color interval (*CEN3*) of fluorescence-tagged line (FTL) produces parental ditype (PD), tetrad type (T), and non-parental ditype (NPD) tetrads. Fluorescent tetrad states were identified using DeepTetrad and the crossover frequency (cM) was calculated using Perkin′s equations (3). Significance between WT and other genotypes was determined using two-sided Welch′s *t*-test.

Genotype	Plant No.	NPD	T	Total	cM	Mean	SD	*P*-value
WT (Col-0)	1	11	360	1,563	13.63	12.90	0.76	
WT (Col-0)	2	4	200	974	11.50			
WT (Col-0)	3	9	279	1,259	13.22			
WT (Col-0)	4	4	242	1,096	12.14			
WT (Col-0)	5	8	192	900	13.33			
WT (Col-0)	6	6	243	1,008	13.84			
WT (Col-0)	7	4	116	567	12.35			
WT (Col-0)	8	11	326	1484	13.21			
mmSUVH T_1	1	7	432	1,253	18.91	15.84	1.21	6.05×10^{-7}
mmSUVH T_1	2	1	52	179	16.20			
mmSUVH T_1	3	1	50	181	15.47			
mmSUVH T_1	4	0	53	153	17.32			
mmSUVH T_1	5	5	296	1,105	14.75			
mmSUVH T_1	6	1	46	164	15.85			
mmSUVH T_1	7	4	157	628	14.41			
mmSUVH T_1	8	9	264	893	17.81			
mmSUVH T_1	9	3	64	262	15.65			
mmSUVH T_1	10	4	131	467	16.60			
mmSUVH T_1	11	4	263	1,013	14.17			
mmSUVH T_1	12	4	123	456	16.12			
mmSUVH T_1	13	4	124	462	16.02			
mmSUVH T_1	14	3	135	475	16.11			
mmSUVH T_1	15	1	160	533	15.57			
mmSUVH T_1	16	5	458	1,686	14.47			
mmSUVH T_1	17	3	173	636	15.02			
mmSUVH T_1	18	11	361	1,446	14.76			
mmSUVH T_2-1	1	1	77	272	15.26	16.44	1.88	2.78×10^{-3}
mmSUVH T_2-1	2	3	133	415	18.19			
mmSUVH T_2-1	3	1	118	428	14.49			
mmSUVH T_2-1	4	2	96	382	14.14			
mmSUVH T_2-1	5	7	224	675	19.70			
mmSUVH T_2-1	6	12	423	1,521	16.27			
mmSUVH T_2-1	7	10	296	1,045	17.03			

mmSUVH T_2-2	1	3	279	1,098	13.52	15.07	1.09	4.65×10^{-3}
mmSUVH T_2-2	2	7	259	1,010	14.90			
mmSUVH T_2-2	3	7	358	1,301	15.37			
mmSUVH T_2-2	4	7	262	913	16.65			
mmSUVH T_2-2	5	7	197	745	16.04			
mmSUVH T_2-2	6	2	134	523	13.96			
mmSUVH T_2-3	1	7	260	953	15.84	15.37	0.83	3.33×10^{-4}
mmSUVH T_2-3	2	4	374	1,350	14.74			
mmSUVH T_2-3	3	1	66	244	14.75			
mmSUVH T_2-3	4	5	185	643	16.72			
mmSUVH T_2-3	5	3	194	739	14.34			
mmSUVH T_2-3	6	3	103	382	15.84			
mmSUVH T_2-4	1	1	100	413	12.83	14.37	1.27	2.30×10^{-2}
mmSUVH T_2-4	2	6	64	323	15.48			
mmSUVH T_2-4	3	5	101	402	16.29			
mmSUVH T_2-4	4	3	102	480	12.50			
mmSUVH T_2-4	5	0	53	195	13.59			
mmSUVH T_2-4	6	2	115	431	14.73			
mmSUVH T_2-4	7	1	44	178	14.04			
mmSUVH T_2-4	8	2	61	236	15.47			

Table S3. *CTL3.9* crossover frequency (cM) in WT and *mmCMT*. The crossover frequency (cM) within the *CTL3.9* interval was measured using CellProfiler as previously described (5) and calculated using the formula: $cM = 100 \times (1 - [1 - 2(N_{Green} + N_{Red})/N_{Total}]^{1/2})$. The ratios R/nR and G/nG denote the ratio of red colored seed number (R) to non-red colored seed number (nR) and the ratio of green colored seed number (G) to non-green colored seed number (nG), respectively. Significance between WT and other genotypes was determined using two-sided Welch's *t*-test.

Genotype	Plant No.	R	G	None	Both	Total	cM	R/nR	G/nG	Mean	SD	*P*-value
WT (Col-0)	1	57	74	140	509	780	18.51	2.64	2.96	17.01	0.66	
WT (Col-0)	2	147	214	398	1422	2,181	18.21	2.56	3.00			
WT (Col-0)	3	74	70	167	575	886	17.85	2.74	2.68			
WT (Col-0)	4	105	146	293	1012	1,556	17.70	2.54	2.91			
WT (Col-0)	5	45	50	106	390	591	17.63	2.79	2.91			
WT (Col-0)	6	63	82	155	603	903	17.61	2.81	3.14			
WT (Col-0)	7	135	146	327	1,143	1,751	17.60	2.70	2.79			
WT (Col-0)	8	129	161	352	1,182	1,824	17.42	2.56	2.79			
WT (Col-0)	9	142	150	359	1,187	1,838	17.40	2.61	2.67			
WT (Col-0)	10	86	95	220	745	1,146	17.29	2.64	2.75			
WT (Col-0)	11	149	173	399	1,328	2,049	17.19	2.58	2.74			
WT (Col-0)	12	130	156	326	1,209	1,821	17.18	2.78	2.99			
WT (Col-0)	13	173	172	395	1,481	2,221	16.97	2.92	2.91			
WT (Col-0)	14	102	122	247	972	1,443	16.96	2.91	3.13			
WT (Col-0)	15	27	33	76	252	388	16.89	2.56	2.77			
WT (Col-0)	16	113	142	332	1,067	1,654	16.83	2.49	2.72			
WT (Col-0)	17	118	125	293	1,042	1,578	16.81	2.78	2.84			
WT (Col-0)	18	140	141	337	1,208	1,826	16.80	2.82	2.83			
WT (Col-0)	19	133	156	337	1,254	1,880	16.78	2.81	3.00			
WT (Col-0)	20	38	42	106	337	523	16.69	2.53	2.63			
WT (Col-0)	21	145	133	346	1,201	1,825	16.61	2.81	2.72			
WT (Col-0)	22	92	84	186	797	1,159	16.56	3.29	3.17			
WT (Col-0)	23	156	147	393	1,323	2,019	16.34	2.74	2.68			
WT (Col-0)	24	165	146	405	1,362	2,078	16.29	2.77	2.65			
WT (Col-0)	25	84	109	260	842	1,295	16.22	2.51	2.76			
WT (Col-0)	26	65	83	191	668	1,007	15.97	2.68	2.93			
WT (Col-0)	27	134	153	350	1,317	,1954	15.96	2.88	3.04			
WT (Col-0)	28	129	135	352	1,187	1,803	15.91	2.70	2.75			
mmCMT T_1	1	30	24	78	254	386	15.13	2.78	2.57	19.28	2.20	8.33×10^{-5}
mmCMT T_1	2	79	78	174	603	934	18.53	2.71	2.69			
mmCMT T_1	3	95	138	208	746	1,187	22.06	2.43	2.92			
mmCMT T_1	4	97	91	208	752	1,148	18.00	2.84	2.76			
mmCMT T_1	5	120	135	238	841	1,334	21.41	2.58	2.73			
mmCMT T_1	6	90	79	241	825	1,235	14.78	2.86	2.73			

mmCMT T_1	7	32	42	82	333	489	16.49	2.94	3.29			
mmCMT T_1	8	124	127	264	974	1,489	18.58	2.81	2.84			
mmCMT T_1	9	62	64	111	441	678	20.73	2.87	2.92			
mmCMT T_1	10	26	30	73	273	402	15.07	2.90	3.06			
mmCMT T_1	11	191	176	351	1,259	1,977	20.71	2.75	2.65			
mmCMT T_1	12	145	168	307	1,106	1,726	20.17	2.63	2.82			
mmCMT T_1	13	78	127	171	669	1,045	22.05	2.51	3.20			
mmCMT T_1	14	109	143	241	953	1,446	19.29	2.77	3.13			
mmCMT T_1	15	126	160	279	1,023	1,588	20.01	2.62	2.92			
mmCMT T_1	16	124	115	268	988	1,495	17.52	2.90	2.81			
mmCMT T_1	17	128	112	250	895	1,385	19.17	2.83	2.66			
mmCMT T_1	18	160	125	269	970	1,524	20.88	2.87	2.55			
mmCMT T_1	19	148	152	318	1,037	1,655	20.16	2.52	2.55			
mmCMT T_1	20	162	163	282	1,,244	1,851	19.45	3.16	3.17			
mmCMT T_1	21	161	167	292	1,242	1,862	19.52	3.06	3.11			
mmCMT T_1	22	126	131	204	847	1,308	22.09	2.90	2.96			
mmCMT T_1	23	59	68	117	411	655	21.76	2.54	2.72			
mmCMT T_2-1	1	181	209	293	1,355	2,038	21.43	3.06	3.30			
mmCMT T_2-1	2	264	178	327	1,326	2,095	23.97	3.15	2.54			
mmCMT T_2-1	3	211	218	334	1,290	2,053	23.71	2.72	2.77	22.81	0.95	1.37×10^{-4}
mmCMT T_2-1	4	284	173	402	1,400	2,259	22.84	2.93	2.29			
mmCMT T_2-1	5	227	181	316	1,352	2,076	22.09	3.18	2.82			
mmCMT T_2-2	1	230	193	354	1,455	2,232	21.20	3.08	2.82			
mmCMT T_2-2	2	197	213	316	1,395	2,121	21.68	3.01	3.13			
mmCMT T_2-2	3	132	229	267	1,252	1,880	21.52	2.79	3.71	21.93	0.65	2.20×10^{-5}
mmCMT T_2-2	4	234	237	390	1,447	2,308	23.07	2.68	2.70			
mmCMT T_2-2	5	165	237	327	1,310	2,039	22.17	2.62	3.14			
mmCMT T_2-3	1	147	181	246	1,059	1,633	22.65	2.82	3.16			
mmCMT T_2-3	2	188	184	364	1,344	2,080	19.86	2.80	2.77			
mmCMT T_2-3	3	211	217	340	1,455	2,223	21.58	2.99	3.03	20.66	1.09	4.55×10^{-4}
mmCMT T_2-3	4	211	164	329	1,362	2,066	20.19	3.19	2.83			
mmCMT T_2-3	5	193	169	372	1,318	2,052	19.55	2.79	2.63			
mmCMT T_2-3	6	229	187	442	1,440	2,298	20.13	2.65	2.42			
mmCMT T_2-4	1	144	177	318	1,196	1,835	19.37	2.71	2.97			
mmCMT T_2-4	2	212	183	329	1,271	1,995	22.28	2.90	2.69			
mmCMT T_2-4	3	155	152	309	1,161	1,777	19.10	2.85	2.83	20.28	1.10	8.07×10^{-4}
mmCMT T_2-4	4	189	161	313	1,274	1,937	20.09	3.09	2.86			
mmCMT T_2-4	5	155	123	271	1,013	1,562	19.75	2.96	2.67			
mmCMT T_2-4	6	207	159	322	1,252	1,940	21.09	3.03	2.67			

Table S4. Crossover frequency (cM) of *CEN3* in WT and *mmCMT*. *CEN3* crossover frequency (cM) was measured by DeepTetrad using the two-color *CEN3* interval. The two-color interval (*CEN3*) of fluorescence-tagged line (FTL) produces parental ditype (PD), tetrad type (T), and non-parental ditype (NPD) tetrads. Fluorescent tetrad states were identified using DeepTetrad and the crossover frequency (cM) was calculated using Perkin′s equations (3). Significance between WT and other genotypes was determined using two-sided Welch′s *t*-test. *mmCMT* indicates *meiMIGS-CMT2/3.*

Genotype	Plant No.	NPD	T	Total	cM	Mean	SD	*P*-value
WT (Col-0)	1	11	360	1,563	13.63	12.90	0.76	
WT (Col-0)	2	4	200	974	11.50			
WT (Col-0)	3	9	279	1,259	13.22			
WT (Col-0)	4	4	242	1,096	12.14			
WT (Col-0)	5	8	192	900	13.33			
WT (Col-0)	6	6	243	1,008	13.84			
WT (Col-0)	7	4	116	567	12.35			
WT (Col-0)	8	11	326	1,484	13.21			
mmCMT T_1	1	7	181	643	17.34	14.81	1.09	4.49×10^{-3}
mmCMT T_1	2	6	361	1,411	14.07			
mmCMT T_1	3	1	310	1,123	14.07			
mmCMT T_1	4	6	239	915	15.03			
mmCMT T_1	5	13	509	2,052	14.30			
mmCMT T_1	6	4	179	721	14.08			
mmCMT T_1	7	5	322	1,189	14.80			
mmCMT T_2-1	1	19	312	1,111	19.17	15.08	1.49	7.51×10^{-4}
mmCMT T_2-1	2	5	157	693	13.49			
mmCMT T_2-1	3	9	398	1,509	14.98			
mmCMT T_2-1	4	21	468	1,945	15.27			
mmCMT T_2-1	5	5	388	1,563	13.37			
mmCMT T_2-1	6	9	552	2,266	13.37			
mmCMT T_2-1	7	9	679	2,520	14.54			
mmCMT T_2-1	8	7	1,022	3,426	15.53			
mmCMT T_2-1	9	4	520	1,730	15.72			
mmCMT T_2-1	10	7	650	2,222	15.57			
mmCMT T_2-1	11	12	547	2,007	15.42			
mmCMT T_2-1	12	8	930	3,362	14.54			
mmCMT T_2-2	1	6	232	937	14.30	15.60	1.69	2.98×10^{-2}
mmCMT T_2-2	2	7	292	1,172	14.25			
mmCMT T_2-2	3	3	128	394	18.53			
mmCMT T_2-2	4	6	229	919	14.42			
mmCMT T_2-2	5	7	117	481	16.53			
mmCMT T_2-3	1	3	369	1,392	13.90	14.24	0.80	6.60×10^{-3}

mmCMT T_2-3	2	12	470	1,795	15.10			
mmCMT T_2-3	3	3	623	2,319	13.82			
mmCMT T_2-3	4	16	765	3,245	13.27			
mmCMT T_2-3	5	3	369	1,392	13.90			
mmCMT T_2-3	6	12	470	1,794	15.11			
mmCMT T_2-3	7	4	224	930	13.33			
mmCMT T_2-3	8	7	645	2,220	15.47			
mmCMT T_2-4	1	7	353	1,426	13.85	13.89	0.50	2.48×10^{-2}
mmCMT T_2-4	2	11	426	1,802	13.65			
mmCMT T_2-4	3	10	619	2,301	14.75			
mmCMT T_2-4	4	8	412	1,645	13.98			
mmCMT T_2-4	5	5	159	714	13.24			

Table S5. *CTL3.9* crossover frequency (cM) in WT and *mmH2A.W*. The crossover frequency (cM) within the *CTL3.9* interval was measured using CellProfiler as previously described (5) and calculated using the formula: $cM = 100 \times (1 - [1 - 2(N_{Green} + N_{Red})/N_{Total}]^{1/2})$. The ratios R/nR and G/nG denote the ratio of red colored seed number (R) to non-red colored seed number (nR) and the ratio of green colored seed number (G) to non-green colored seed number (nG), respectively. Significance between WT and other genotypes was determined using two-sided Welch's *t*-test.

Genotype	Plant No.	R	G	None	Both	Total	cM	R/nR	G/nG	Mean	SD	*P*-value
WT (Col-0)	1	74	70	167	575	886	17.85	2.74	2.68	16.98	0.50	
WT (Col-0)	2	105	146	293	1,012	1,556	17.70	2.54	2.91			
WT (Col-0)	3	45	50	106	390	591	17.63	2.79	2.91			
WT (Col-0)	4	63	82	155	603	903	17.61	2.81	3.14			
WT (Col-0)	5	135	146	327	1,143	1,751	17.60	2.70	2.79			
WT (Col-0)	6	129	161	352	1,182	1,824	17.42	2.56	2.79			
WT (Col-0)	7	142	150	359	1,187	1,838	17.40	2.61	2.67			
WT (Col-0)	8	86	95	220	745	1,146	17.29	2.64	2.75			
WT (Col-0)	9	149	173	399	1,328	2,049	17.19	2.58	2.74			
WT (Col-0)	10	130	156	326	1,209	1,821	17.18	2.78	2.99			
WT (Col-0)	11	173	172	395	1,481	2,221	16.97	2.92	2.91			
WT (Col-0)	12	102	122	247	972	1,443	16.96	2.91	3.13			
WT (Col-0)	13	27	33	76	252	388	16.89	2.56	2.77			
WT (Col-0)	14	113	142	332	1,067	1,654	16.83	2.49	2.72			
WT (Col-0)	15	118	125	293	1,042	1,578	16.81	2.78	2.84			
WT (Col-0)	16	140	141	337	1,208	1,826	16.80	2.82	2.83			
WT (Col-0)	17	133	156	337	1,254	1,880	16.78	2.81	3.00			
WT (Col-0)	18	38	42	106	337	523	16.69	2.53	2.63			
WT (Col-0)	19	145	133	346	1,201	1,825	16.61	2.81	2.72			
WT (Col-0)	20	92	84	186	797	1,159	16.56	3.29	3.17			
WT (Col-0)	21	156	147	393	1,323	2,019	16.34	2.74	2.68			
WT (Col-0)	22	165	146	405	1,362	2,078	16.29	2.77	2.65			
WT (Col-0)	23	84	109	260	842	1,295	16.22	2.51	2.76			
WT (Col-0)	24	65	83	191	668	1,007	15.97	2.68	2.93			
mmH2A.W T_1	1	185	191	289	1,171	1,836	23.16	2.83	2.87	20.82	1.45	1.45×10^{-9}
mmH2A.W T_1	2	227	238	352	1,525	2,342	22.35	2.97	3.04			
mmH2A.W T_1	3	94	135	173	740	1,142	22.61	2.71	3.28			
mmH2A.W T_1	4	114	120	208	786	1,228	21.33	2.74	2.81			
mmH2A.W T_1	5	210	206	319	1,446	2,181	21.35	3.15	3.12			
mmH2A.W T_1	6	152	163	307	1,104	1,726	20.31	2.67	2.76			
mmH2A.W T_1	7	108	119	214	869	1,310	19.16	2.93	3.07			
mmH2A.W T_1	8	104	165	242	994	1,505	19.84	2.70	3.35			
mmH2A.W T_1	9	38	55	85	309	487	21.38	2.48	2.96			
mmH2A.W T_1	10	128	124	257	926	1,435	19.45	2.77	2.73			

mmH2A.W T_1	11	84	96	176	647	1,003	19.93	2.69	2.86			
mmH2A.W T_1	12	164	135	254	893	1,446	23.42	2.72	2.46			
mmH2A.W T_1	13	142	170	282	1,150	1744	19.86	2.86	3.11			
mmH2A.W T_1	14	172	139	308	1,072	1,691	20.49	2.78	2.52			
mmH2A.W T_1	15	101	130	242	858	1,331	19.2	2.58	2.88			
mmH2A.W T_1	16	45	57	83	330	515	22.29	2.68	3.02			
mmH2A.W T_1	17	137	121	269	1,028	1,555	18.26	2.99	2.83			
mmH2A.W T_1	18	95	103	189	694	1,081	20.4	2.70	2.81			
mmH2A.W T_2-1	1	214	185	296	1,237	1,932	23.39	3.02	2.79	25.69	1.40	1.53×10^{-3}
mmH2A.W T_2-1	2	215	277	321	1,384	2,197	25.7	2.67	3.1			
mmH2A.W T_2-1	3	228	245	285	1,280	2,038	26.8	2.85	2.97			
mmH2A.W T_2-1	4	301	271	375	1,513	2,460	26.86	2.81	2.64			
mmH2A.W T_2-2	1	229	281	323	1,444	2,277	25.7	2.77	3.13	24.09	1.60	7.79×10^{-4}
mmH2A.W T_2-2	2	250	208	299	1,266	2,023	26.03	2.99	2.68			
mmH2A.W T_2-2	3	178	183	274	1,080	1,715	23.91	2.75	2.79			
mmH2A.W T_2-2	4	153	244	333	1,312	2,042	21.82	2.54	3.2			
mmH2A.W T_2-2	5	209	256	333	1,487	2,285	22.99	2.88	3.22			
mmH2A.W T_2-3	1	214	207	278	1,125	1,824	26.63	2.76	2.71	24.23	1.49	9.14×10^{-5}
mmH2A.W T_2-3	2	128	142	184	751	1,205	25.71	2.70	2.86			
mmH2A.W T_2-3	3	183	196	290	1,189	1,858	23.06	2.82	2.93			
mmH2A.W T_2-3	4	192	205	270	1,246	1,913	23.52	3.03	3.14			
mmH2A.W T_2-3	5	158	216	304	1,206	1,884	22.35	2.62	3.08			
mmH2A.W T_2-3	6	228	176	289	1,213	1,906	24.1	3.10	2.69			
mmH2A.W T_2-4	1	223	188	264	1,171	1,846	25.52	3.08	2.79	22.69	1.57	1.71×10^{-3}
mmH2A.W T_2-4	2	173	165	297	1,018	1,653	23.12	2.58	2.52			
mmH2A.W T_2-4	3	219	214	342	1,443	2,218	21.93	2.99	2.95			
mmH2A.W T_2-4	4	169	168	286	1,170	1,793	21	2.95	2.94			
mmH2A.W T_2-4	5	180	247	332	1,433	2,192	21.87	2.79	3.28			

Table S6. Crossover frequency (cM) of *CEN3* in WT and *mmH2A.W*. *CEN3* crossover frequency (cM) was measured by DeepTetrad using the two-color *CEN3* interval. The two-color interval (*CEN3*) of fluorescence-tagged line (FTL) produces parental ditype (PD), tetrad type (T), and non-parental ditype (NPD) tetrads. Fluorescent tetrad states were identified using DeepTetrad and the crossover frequency (cM) was calculated using Perkin′s equations (3). Significance between WT and other genotypes was determined using two-sided Welch′s *t*-test.

Genotype	Plant No.	NPD	T	Total	cM	Mean	SD	*P*-value
WT (Col-0)	1	11	360	1,563	13.63	12.90	0.76	
WT (Col-0)	2	4	200	974	11.50			
WT (Col-0)	3	9	279	1,259	13.22			
WT (Col-0)	4	4	242	1,096	12.14			
WT (Col-0)	5	8	192	900	13.33			
WT (Col-0)	6	6	243	1,008	13.84			
WT (Col-0)	7	4	116	567	12.35			
WT (Col-0)	8	11	326	1,484	13.21			
mmH2A.W T_1	1	4	297	1,047	15.33	16.20	1.56	3.58×10^{-6}
mmH2A.W T_1	2	3	259	979	14.15			
mmH2A.W T_1	3	3	119	421	16.27			
mmH2A.W T_1	4	3	181	662	15.03			
mmH2A.W T_1	5	7	103	364	19.92			
mmH2A.W T_1	6	9	324	1,176	16.07			
mmH2A.W T_1	7	1	58	211	15.17			
mmH2A.W T_1	8	5	213	798	15.23			
mmH2A.W T_1	9	3	213	780	14.81			
mmH2A.W T_1	10	3	87	312	16.83			
mmH2A.W T_1	11	6	59	258	18.41			
mmH2A.W T_1	12	5	123	425	18.00			
mmH2A.W T_1	13	6	330	1,218	15.02			
mmH2A.W T_1	14	12	288	1,082	16.64			
mmH2A.W T_2-1	1	2	154	515	16.12	16.29	1.26	2.94×10^{-6}
mmH2A.W T_2-1	2	10	418	1,619	14.76			
mmH2A.W T_2-1	3	16	522	1,745	17.71			
mmH2A.W T_2-1	4	10	492	1,785	15.46			
mmH2A.W T_2-1	5	4	123	389	18.89			
mmH2A.W T_2-1	6	7	398	1,412	15.58			
mmH2A.W T_2-1	7	4	141	463	17.82			
mmH2A.W T_2-1	8	3	136	498	15.46			
mmH2A.W T_2-1	9	7	316	1,068	16.76			
mmH2A.W T_2-1	10	8	405	1,495	15.15			
mmH2A.W T_2-1	11	5	163	623	15.49			
mmH2A.W T_2-2	1	2	114	419	15.04	15.80	0.71	1.28×10^{-6}

mmH2A.W T_2-2	2	4	121	442	16.40			
mmH2A.W T_2-2	3	21	621	2,148	17.39			
mmH2A.W T_2-2	4	10	527	1,894	15.50			
mmH2A.W T_2-2	5	4	131	448	17.30			
mmH2A.W T_2-2	6	7	283	1,055	15.40			
mmH2A.W T_2-2	7	3	187	645	15.89			
mmH2A.W T_2-2	8	8	237	933	15.27			
mmH2A.W T_2-2	9	2	115	404	15.72			
mmH2A.W T_2-2	10	8	156	661	15.43			
mmH2A.W T_2-2	11	5	432	1,442	16.02			
mmH2A.W T_2-2	12	0	57	184	15.49			
mmH2A.W T_2-2	13	4	220	795	15.35			
mmH2A.W T_2-2	14	2	47	194	15.21			
mmH2A.W T_2-2	15	8	369	1,385	15.05			
mmH2A.W T_2-2	16	7	167	637	16.41			
mmH2A.W T_2-3	1	16	346	1,135	19.47	16.79	1.70	9.43×10^{-7}
mmH2A.W T_2-3	2	2	73	247	17.21			
mmH2A.W T_2-3	3	6	307	1,153	14.87			
mmH2A.W T_2-3	4	2	27	94	20.74			
mmH2A.W T_2-3	5	11	355	1,188	17.72			
mmH2A.W T_2-3	6	2	149	453	17.77			
mmH2A.W T_2-3	7	6	123	499	15.93			
mmH2A.W T_2-3	8	4	361	1,204	15.99			
mmH2A.W T_2-3	9	5	154	522	17.62			
mmH2A.W T_2-3	10	2	121	418	15.91			
mmH2A.W T_2-3	11	1	87	278	16.73			
mmH2A.W T_2-3	12	1	81	282	15.43			
mmH2A.W T_2-3	13	4	94	401	14.71			
mmH2A.W T_2-3	14	4	85	363	15.01			
mmH2A.W T_2-4	1	8	365	1,333	15.49	17.32	1.61	1.94×10^{-7}
mmH2A.W T_2-4	2	6	172	563	18.47			
mmH2A.W T_2-4	3	1	134	415	16.87			
mmH2A.W T_2-4	4	5	134	450	18.22			
mmH2A.W T_2-4	5	3	68	239	17.99			
mmH2A.W T_2-4	6	5	102	350	18.86			
mmH2A.W T_2-4	7	4	97	414	14.61			
mmH2A.W T_2-4	8	2	131	438	16.32			
mmH2A.W T_2-4	9	4	134	487	16.22			
mmH2A.W T_2-4	10	2	111	327	18.81			

mmH2A.W T_2-4	11	13	208	691	20.69			
mmH2A.W T_2-4	12	8	358	1,258	16.14			
mmH2A.W T_2-4	13	15	827	2,795	16.40			

Table S7. *CTL3.9* crossover frequency (cM) in WT Col-0 × L*er*, *mmH2A.W* × L*er*, *mmSUVH* × L*er*, and *mmH2A.W mmSUVH* × L*er* F1 hybrid plants. The crossover frequency (cM) within the *CTL3.9* interval was measured using CellProfiler as previously described (5) and calculated using the formula: $cM = 100 \times (1 - [1 - 2(N_{Green} + N_{Red})/N_{Total}]^{1/2})$. The ratios R/nR and G/nG denote the ratio of red colored seed number (R) to non-red colored seed number (nR) and the ratio of green colored seed number (G) to non-green colored seed number (nG), respectively. One-way analysis of variance (ANOVA) followed by Tukey-Kramer's test was used to examine significance between genotypes.

Col-0 × L*er*	Plant No.	R	G	None	Both	Total	cM	R/nR	G/nG	Mean	SD	*P*-value
WT	1	101	87	177	547	912	23.34	2.45	2.28	21.17	1.24	
WT	2	58	70	127	399	654	21.99	2.32	2.54			
WT	3	102	106	121	674	1,003	23.50	3.42	3.50			
WT	4	72	93	165	600	930	19.68	2.60	2.92			
WT	5	67	83	152	527	829	20.12	2.53	2.79			
WT	6	66	86	137	548	837	20.20	2.75	3.12			
WT	7	88	112	161	632	993	22.72	2.64	2.99			
WT	8	85	97	137	660	979	20.74	3.18	3.41			
WT	9	98	96	190	678	1,062	20.33	2.71	2.69			
WT	10	106	106	192	759	1,163	20.29	2.90	2.90			
WT	11	93	89	155	672	1,009	20.05	3.14	3.07			
WT	12	95	156	238	771	1,260	22.44	2.20	2.78			
WT	13	99	100	226	649	1,074	20.66	2.29	2.30			
WT	14	87	113	193	626	1,019	22.06	2.33	2.64			
WT	15	119	94	217	753	1,183	20.01	2.80	2.52			
WT	16	94	118	201	736	1,149	20.57	2.60	2.89			
mmH2A.W	1	214	201	1,346	322	2,083	22.44	2.98	2.89	25.28	2.58	WT 4.43×10^{-5}<br>*mmSUVH* 0.444<br>*mmH2A.W mmSUVH* 0.950
mmH2A.W	2	129	112	727	154	1,122	24.47	3.22	2.96			
mmH2A.W	3	115	131	549	177	972	29.73	2.16	2.33			
mmH2A.W	4	148	114	692	176	1,130	26.77	2.90	2.49			
mmH2A.W	5	122	107	786	189	1,204	21.29	3.07	2.87			
mmH2A.W	6	167	149	947	239	1,502	23.89	2.87	2.70			
mmH2A.W	7	144	137	165	765	1,211	26.79	3.01	2.92			
mmH2A.W	8	172	169	184	890	1,415	28.03	3.01	2.97			
mmH2A.W	9	137	138	188	834	1,297	24.11	2.98	2.99			
mmSUVH	1	121	157	205	857	1,340	23.51	2.70	3.11	24.05	1.92	WT 7.63×10^{-4}<br>*mmH2A.W* 0.444<br>*mmH2A.W mmSUVH* 0.149
mmSUVH	2	93	119	162	651	1,025	23.43	2.65	3.02			
mmSUVH	3	102	135	193	722	1,152	23.28	2.51	2.91			
mmSUVH	4	123	153	206	752	1,234	25.66	2.44	2.75			
mmSUVH	5	98	130	187	692	1,107	23.31	2.49	2.88			
mmSUVH	6	115	128	201	756	1,200	22.86	2.65	2.80			
mmSUVH	7	114	122	197	767	1,200	22.11	2.76	2.86			

mmSUVH	8	120	146	186	844	1,296	23.22	2.90	3.24			
mmSUVH	9	122	159	197	937	1,415	22.36	2.97	3.44			
mmSUVH	10	52	55	78	314	499	24.43	2.75	2.84			
mmSUVH	11	95	110	143	559	907	25.98	2.58	2.81			
mmSUVH	12	132	149	221	870	1,372	23.16	2.71	2.89			
mmSUVH	13	135	173	240	1038	1,586	21.80	2.84	3.23			
mmSUVH	14	175	224	237	937	1,573	29.81	2.41	2.82			
mmSUVH	15	173	194	274	1088	1,729	24.14	2.69	2.87			
mmSUVH	16	162	192	240	983	1,577	25.77	2.65	2.92			
mmH2A.W mmSUVH	1	118	124	179	701	1,122	24.59	2.70	2.78	25.76	1.94	WT 3.10×10^{-6} *mmH2A.W* 0.950 *mmSUVH* 0.149
mmH2A.W mmSUVH	2	127	135	199	807	1,268	23.40	2.80	2.89			
mmH2A.W mmSUVH	3	166	153	197	840	1,356	27.23	2.87	2.74			
mmH2A.W mmSUVH	4	143	165	187	863	1,358	26.08	2.86	3.12			
mmH2A.W mmSUVH	5	114	123	193	793	1,223	21.74	2.87	2.98			
mmH2A.W mmSUVH	6	163	139	212	726	1,240	28.38	2.53	2.31			
mmH2A.W mmSUVH	7	125	134	220	680	1,159	25.63	2.27	2.36			
mmH2A.W mmSUVH	8	128	146	152	725	1,151	27.62	2.86	3.11			
mmH2A.W mmSUVH	9	79	73	99	425	676	25.82	2.93	2.80			
mmH2A.W mmSUVH	10	130	125	190	643	1,088	27.11	2.45	2.40			

Table S8. *CTL3.9* crossover frequency (cM) in WT and *h2a.w* mutants. The crossover frequency (cM) within the *CTL3.9* interval was measured using CellProfiler as previously described (5) and calculated using the formula: $cM = 100 \times (1 - [1 - 2(N_{Green} + N_{Red})/N_{Total}]^{1/2})$. The ratios R/nR and G/nG denote the ratio of red colored seed number (R) to non-red colored seed number (nR) and the ratio of green colored seed number (G) to non-green colored seed number (nG), respectively. ANOVA followed by Tukey-Kramer's test was used to examine significance between genotypes. The *P*-value for the comparison between WT and mutant is shown. The ANOVA result for all pairwise comparison are available in *SI Appendix,* Dataset S3.

Genotype	Plant No.	R	G	None	Both	Total	cM	R/nR	G/nG	Mean	SD	*P*-value
WT (Col-0)	1	152	180	338	1,358	2,028	17.99	2.92	3.14			
WT (Col-0)	2	183	180	411	1,518	2,292	17.34	2.88	2.86			
WT (Col-0)	3	161	152	297	1,231	1,841	18.76	3.10	3.02			
WT (Col-0)	4	155	164	352	1,408	2,079	16.75	3.03	3.10			
WT (Col-0)	5	143	150	314	1,221	1,828	17.57	2.94	3.00			
WT (Col-0)	6	124	119	240	1,036	1,519	17.53	3.23	3.17			
WT (Col-0)	7	149	171	328	1,297	1,945	18.09	2.90	3.08			
WT (Col-0)	8	173	167	364	1,396	2,100	17.77	2.95	2.91			
WT (Col-0)	9	143	150	317	1,183	1,793	17.95	2.84	2.90			
WT (Col-0)	10	125	156	320	1,215	1,816	16.90	2.82	3.08			
WT (Col-0)	11	135	132	284	1,098	1,649	17.77	2.96	2.94			
WT (Col-0)	12	121	134	308	1,121	1,684	16.50	2.81	2.93			
WT (Col-0)	13	142	161	374	1,338	2,015	16.38	2.77	2.91			
WT (Col-0)	14	157	157	317	1,384	2,015	17.03	3.25	3.25			
WT (Col-0)	15	128	148	296	1,109	1,681	18.05	2.79	2.96			
WT (Col-0)	16	180	171	357	1,317	2,025	19.17	2.84	2.77	17.90	1.11	
WT (Col-0)	17	169	146	366	1,361	2,042	16.84	2.99	2.82			
WT (Col-0)	18	113	153	235	915	1,416	20.99	2.65	3.07			
WT (Col-0)	19	117	135	242	933	1,427	19.58	2.79	2.97			
WT (Col-0)	20	97	95	187	792	1,171	18.02	3.15	3.12			
WT (Col-0)	21	110	127	264	961	1,462	17.79	2.74	2.91			
WT (Col-0)	22	134	116	231	997	1,478	18.65	3.26	3.05			
WT (Col-0)	23	156	149	299	1,126	1,730	19.54	2.86	2.80			
WT (Col-0)	24	139	139	285	1,216	1,779	17.09	3.20	3.20			
WT (Col-0)	25	101	121	256	971	1,449	16.72	2.84	3.06			
WT (Col-0)	26	106	95	222	851	1,274	17.27	3.02	2.88			
WT (Col-0)	27	84	103	226	780	1,193	17.14	2.63	2.85			
WT (Col-0)	28	58	82	137	606	883	17.36	3.03	3.53			
WT (Col-0)	29	123	122	235	862	1,342	20.32	2.76	2.75			
WT (Col-0)	30	151	158	349	1,267	1,925	17.60	2.80	2.85			
WT (Col-0)	31	134	166	279	1,127	1,706	19.48	2.83	3.13			

WT (Col-0)	32	105	93	213	828	1,239	17.51	3.05	2.90			
WT (Col-0)	33	137	114	276	1,055	1,582	17.38	3.06	2.83			
WT (Col-0)	34	143	150	347	1,294	1,934	16.51	2.89	2.95			
WT (Col-0)	35	125	123	244	939	1,431	19.17	2.90	2.88			
WT (Col-0)	36	75	85	167	657	984	17.85	2.90	3.07			
h2a.w.6-2	1	160	200	347	1,312	2,019	19.79	2.69	2.98			
h2a.w.6-2	2	185	196	378	1,439	2,198	19.17	2.83	2.90			
h2a.w.6-2	3	184	179	334	1,319	2,016	20.01	2.93	2.89			
h2a.w.6-2	4	182	191	357	1,481	2,211	18.60	3.03	3.10			
h2a.w.6-2	5	123	127	198	891	1,339	20.84	3.12	3.17			
h2a.w.6-2	6	95	86	217	736	1,134	17.49	2.74	2.63			
h2a.w.6-2	7	172	185	352	1,341	2,050	19.27	2.82	2.91	19.48	1.33	1.15×10^{-3}
h2a.w.6-2	8	107	93	163	651	1,014	22.18	2.96	2.76			
h2a.w.6-2	9	107	121	219	860	1,307	19.31	2.84	3.01			
h2a.w.6-2	10	170	159	313	1,124	1,766	20.79	2.74	2.66			
h2a.w.6-2	11	184	185	341	1,362	2,072	19.76	2.94	2.95			
h2a.w.6-2	12	129	149	294	1,175	1,747	17.43	2.94	3.13			
h2a.w.6-2	13	142	145	286	1,126	1,699	18.63	2.94	2.97			
h2a.w.7-1	1	146	159	283	1,252	1,840	18.24	3.16	3.29			
h2a.w.7-1	2	135	154	259	1,106	1,654	19.34	3.00	3.20			
h2a.w.7-1	3	143	160	283	1,224	1,810	18.44	3.09	3.25			
h2a.w.7-1	4	191	177	367	1,525	2,260	17.88	3.15	3.05			
h2a.w.7-1	5	155	157	303	1,181	1,796	19.22	2.90	2.92			
h2a.w.7-1	6	168	194	311	1,202	1,875	21.65	2.71	2.91			
h2a.w.7-1	7	171	176	340	1,356	2,043	18.74	2.96	3.00			
h2a.w.7-1	8	176	140	351	1,142	1,809	19.34	2.68	2.43			
h2a.w.7-1	9	194	169	374	1,451	2,188	18.26	3.03	2.85			
h2a.w.7-1	10	138	164	316	1,171	1,789	18.61	2.73	2.94	19.25	0.97	1.27×10^{-5}
h2a.w.7-1	11	190	150	318	1,200	1,858	20.37	2.97	2.66			
h2a.w.7-1	12	175	161	317	1,266	1,919	19.39	3.01	2.90			
h2a.w.7-1	13	213	160	298	1,261	1,932	21.65	3.22	2.78			
h2a.w.7-1	14	184	174	326	1,354	2,038	19.46	3.08	3.00			
h2a.w.7-1	15	189	203	364	1,477	2,233	19.45	2.94	3.04			
h2a.w.7-1	16	144	170	299	1,200	1,813	19.15	2.87	3.09			
h2a.w.7-1	17	181	177	316	1,347	2,021	19.64	3.10	3.07			
h2a.w.7-1	18	81	95	151	726	1,053	18.41	3.28	3.54			
h2a.w.7-1	19	127	149	236	1,044	1,556	19.67	3.04	3.29			
h2a.w.7-1	20	127	168	287	1,166	1,748	18.61	2.84	3.22			

h2a.w.7-1	21	160	159	327	1,230	1,876	18.76	2.86	2.85			
h2a.w.7-1	22	146	172	271	1,250	1,839	19.12	3.15	3.41			
h2a.w.12-1	1	97	128	249	848	1,322	18.78	2.51	2.82	18.53	1.06	9.45×10^{-2}
h2a.w.12-1	2	131	178	334	1,207	1,850	18.39	2.61	2.98			
h2a.w.12-1	3	105	98	210	844	1,257	17.72	3.08	2.99			
h2a.w.12-1	4	165	160	339	1,251	1,915	18.72	2.84	2.80			
h2a.w.12-1	5	146	140	257	1,080	1,623	19.53	3.09	3.03			
h2a.w.12-1	6	181	179	369	1,437	2,166	18.29	2.95	2.94			
h2a.w.12-1	7	64	68	143	586	861	16.73	3.08	3.16			
h2a.w.12-1	8	61	76	148	605	890	16.81	2.97	3.26			
h2a.w.12-1	9	59	72	129	500	760	19.05	2.78	3.04			
h2a.w.12-1	10	80	81	161	554	876	20.48	2.62	2.63			
h2a.w.12-1	11	67	57	134	461	719	19.06	2.76	2.58			
h2a.w.12-1	12	59	63	128	467	717	18.78	2.75	2.83			
h2a.w.6,7	1	94	51	118	534	797	20.24	3.72	2.76	20.53	1.21	2.73×10^{-5}
h2a.w.6,7	2	85	64	133	586	868	18.96	3.41	2.98			
h2a.w.6,7	3	73	50	123	488	734	18.46	3.24	2.74			
h2a.w.6,7	4	106	72	142	628	948	20.98	3.43	2.82			
h2a.w.6,7	5	106	84	164	718	1,072	19.66	3.32	2.97			
h2a.w.6,7	6	29	35	57	223	344	20.76	2.74	3.00			
h2a.w.6,7	7	31	26	51	187	295	21.67	2.83	2.60			
h2a.w.6,7	8	34	24	53	184	295	22.10	2.83	2.39			
h2a.w.6,7	9	32	20	48	180	280	20.72	3.12	2.50			
h2a.w.6,7	10	29	28	47	190	294	21.75	2.92	2.87			
h2a.w.6,12	1	103	89	177	735	1,104	19.24	3.15	2.94	19.37	1.43	2.38×10^{-2}
h2a.w.6,12	2	140	126	206	910	1,382	21.57	3.16	2.99			
h2a.w.6,12	3	160	139	247	1,101	1,647	20.19	3.27	3.05			
h2a.w.6,12	4	175	153	281	1,234	1,843	19.75	3.25	3.04			
h2a.w.6,12	5	197	176	309	1,333	2,015	20.64	3.15	2.98			
h2a.w.6,12	6	168	153	306	1,334	1,961	17.99	3.27	3.14			
h2a.w.6,12	7	145	176	331	1,347	1,999	17.61	2.94	3.20			
h2a.w.6,12	8	178	182	370	1,475	2,205	17.93	2.99	3.02			
h2a.w.7,12	1	115	135	247	966	1,463	18.87	2.83	3.04	19.54	1.06	1.11×10^{-2}
h2a.w.7,12	2	70	82	145	601	898	18.67	2.96	3.18			
h2a.w.7,12	3	100	89	193	786	1,168	17.76	3.14	2.99			
h2a.w.7,12	4	111	117	234	898	1,360	18.47	2.87	2.94			
h2a.w.7,12	5	148	164	274	1,092	1,678	20.75	2.83	2.98			
h2a.w.7,12	6	93	121	215	813	1,242	19.04	2.70	3.03			

h2a.w.7,12	7	108	127	233	915	1,383	18.75	2.84	3.06			
h2a.w.7,12	8	150	137	243	1,056	1,586	20.12	3.17	3.04			
h2a.w.7,12	9	158	174	312	1,261	1,905	19.29	2.92	3.05			
h2a.w.7,12	10	176	213	368	1,411	2,168	19.93	2.73	2.99			
h2a.w.7,12	11	94	112	173	684	1,063	21.74	2.73	2.98			
h2a.w.7,12	12	150	188	315	1,216	1,869	20.11	2.72	3.02			
h2a.w.7,12	13	161	168	328	1,214	1,871	19.48	2.77	2.83			
h2a.w.7,12	14	187	209	357	1,393	2,146	20.57	2.79	2.94			
h2a.w-2	1	127	113	213	860	1,313	20.35	3.03	2.86	20.44	1.12	4.92×10^{-5}
h2a.w-2	2	178	188	325	1,368	2,059	19.72	3.01	3.09			
h2a.w-2	3	157	168	313	1,268	1,906	18.82	2.96	3.06			
h2a.w-2	4	180	186	319	1,374	2,059	19.72	3.08	3.13			
h2a.w-2	5	199	191	346	1,478	2,214	19.52	3.12	3.06			
h2a.w-2	6	161	161	292	1,088	1,702	21.16	2.76	2.76			
h2a.w-2	7	165	177	284	1,091	1,717	22.44	2.72	2.82			
h2a.w-2	8	166	154	294	1,096	1,710	20.90	2.82	2.72			
h2a.w-2	9	196	210	363	1,360	2,129	21.35	2.72	2.81			
h2a.w-3	1	200	189	352	1,440	2,181	19.80	3.03	2.95	20.29	0.64	2.28×10^{-12}
h2a.w-3	2	212	181	353	1,462	2,208	19.75	3.13	2.91			
h2a.w-3	3	206	174	323	1,457	2,160	19.49	3.35	3.08			
h2a.w-3	4	172	172	322	1,285	1,951	19.54	2.95	2.95			
h2a.w-3	5	160	179	280	1,212	1,831	20.65	2.99	3.16			
h2a.w-3	6	191	169	323	1,285	1,968	20.37	3.00	2.83			
h2a.w-3	7	190	176	340	1,292	1,998	20.40	2.87	2.77			
h2a.w-3	8	186	192	352	1,319	2,049	20.56	2.77	2.81			
h2a.w-3	9	191	202	302	1,372	2,067	21.28	3.10	3.19			
h2a.w-3	10	204	176	301	1,281	1,962	21.73	3.11	2.89			
h2a.w-3	11	194	187	363	1,343	2,087	20.32	2.79	2.75			
h2a.w-3	12	189	173	355	1,263	1,980	20.35	2.75	2.64			
h2a.w-3	13	186	174	380	1,312	2,052	19.43	2.70	2.63			
h2a.w-3	14	158	217	350	1,318	2,043	20.45	2.60	3.02			
h2a.w-3	15	134	130	257	930	1,451	20.24	2.75	2.71			
h2a.w-2 × *h2a.w-3*	1	177	168	340	1,259	1,944	19.68	2.83	2.76	20.04	0.64	1.26×10^{-8}
h2a.w-2 × *h2a.w-3*	2	191	191	353	1,409	2,144	19.77	2.94	2.94			
h2a.w-2 × *h2a.w-3*	3	192	197	354	1,418	2,161	20.00	2.92	2.96			
h2a.w-2 × *h2a.w-3*	4	146	160	292	1,147	1,745	19.42	2.86	2.98			
h2a.w-2 × *h2a.w-3*	5	156	179	340	1,179	1,854	20.09	2.57	2.74			
h2a.w-2 × *h2a.w-3*	6	110	174	258	970	1,512	20.98	2.50	3.11			

h2a.w-2 × h2a.w-3	7	148	159	260	1,195	1,762	19.28	3.21	3.32			
h2a.w-2 × h2a.w-3	8	158	141	277	1,069	1,645	20.22	2.94	2.78			
h2a.w-2 × h2a.w-3	9	129	134	233	947	1,443	20.28	2.93	2.99			
h2a.w-2 × h2a.w-3	10	154	138	273	1,117	1,682	19.20	3.09	2.94			
h2a.w-2 × h2a.w-3	11	114	129	233	873	1,349	20.02	2.73	2.89			
h2a.w-2 × h2a.w-3	12	125	127	203	876	1,331	21.18	3.03	3.06			
h2a.w-2 × h2a.w-3	13	129	139	250	1,007	1,525	19.47	2.92	3.02			
h2a.w-2 × h2a.w-3	14	108	101	158	733	1,100	21.26	3.25	3.14			
h2a.w-2 × h2a.w-3	15	123	127	244	888	1,382	20.11	2.73	2.77			
h2a.w-2 × h2a.w-3	16	84	107	162	723	1,076	19.69	3.00	3.37			

Table S9. Crossover frequency (cM) of *CEN3* in WT and *h2a.w* mutants. *CEN3* crossover frequency (cM) was measured by DeepTetrad using the two-color *CEN3* interval. The two-color interval (*CEN3*) of fluorescence-tagged line (FTL) produces parental ditype (PD), tetrad type (T), and non-parental ditype (NPD) tetrads. Fluorescent tetrad states were identified using DeepTetrad and the crossover frequency (cM) was calculated using Perkin′s equations (3). ANOVA followed by Tukey-Kramer′s test was used to examine significance between genotypes.

Genotype	Plant No.	NPD	T	Total	cM	Mean	SD	*P*-value
WT (Col-0)	1	3	366	1,445	13.29			
WT (Col-0)	2	2	163	680	12.87			
WT (Col-0)	3	3	301	1,206	13.23			
WT (Col-0)	4	4	264	1,031	13.97			
WT (Col-0)	5	7	289	1,137	14.56			
WT (Col-0)	6	5	491	1,803	14.45			
WT (Col-0)	7	5	400	1,483	14.50			
WT (Col-0)	8	2	232	936	13.03			
WT (Col-0)	9	3	382	1,335	14.98			
WT (Col-0)	10	2	87	406	12.19			
WT (Col-0)	11	0	367	1,385	13.25			
WT (Col-0)	12	5	580	2,153	14.17			
WT (Col-0)	13	5	375	1,390	14.57			
WT (Col-0)	14	3	415	1,669	12.97			
WT (Col-0)	15	4	235	970	13.35			
WT (Col-0)	16	0	367	1,433	12.81	13.67	0.92	
WT (Col-0)	17	4	518	1,823	14.87			
WT (Col-0)	18	3	238	1,125	11.38			
WT (Col-0)	19	3	376	1,545	12.75			
WT (Col-0)	20	7	353	1,418	13.93			
WT (Col-0)	21	2	391	1,386	14.54			
WT (Col-0)	22	5	592	2,373	13.11			
WT (Col-0)	23	10	637	2,715	12.84			
WT (Col-0)	24	3	525	2,180	12.45			
WT (Col-0)	25	4	669	2,499	13.87			
WT (Col-0)	26	5	527	1,898	14.67			
WT (Col-0)	27	7	684	2,416	15.02			
WT (Col-0)	28	5	475	1,701	14.84			
WT (Col-0)	29	5	238	981	13.66			
WT (Col-0)	30	2	752	2,821	13.54			
WT (Col-0)	31	3	458	1,670	14.25			
h2a.w.6-2	1	5	321	1,145	15.33	16.13	0.78	WT 1.15×10^{-9}

h2a.w.6-2	2	7	384	1,379	15.45			*h2a.w.7*<br>0.439<br>*h2a.w.6,7*<br>0.141<br>*h2a.w-2*<br>0.132
h2a.w.6-2	3	9	405	1,523	15.07			
h2a.w.6-2	4	8	404	1,344	16.82			
h2a.w.6-2	5	3	283	930	16.18			
h2a.w.6-2	6	5	194	693	16.16			
h2a.w.6-2	7	6	244	830	16.87			
h2a.w.6-2	8	9	542	1,738	17.15			
h2a.w.7-1	1	9	476	1,749	15.15	15.49	0.46	WT<br>4.47×10^{-8}<br>*h2a.w.6*<br>0.439<br>*h2a.w.6,7*<br>0.965<br>*h2a.w-2*<br>0.937
h2a.w.7-1	2	2	235	816	15.13			
h2a.w.7-1	3	8	470	1,789	14.48			
h2a.w.7-1	4	7	181	694	16.07			
h2a.w.7-1	5	5	313	1,125	15.24			
h2a.w.7-1	6	2	317	1,068	15.40			
h2a.w.7-1	7	9	493	1,737	15.75			
h2a.w.7-1	8	16	496	1,879	15.75			
h2a.w.7-1	9	18	804	2,900	15.72			
h2a.w.7-1	10	17	728	2,696	15.39			
h2a.w.7-1	11	8	418	1,445	16.12			
h2a.w.7-1	12	17	757	2,818	15.24			
h2a.w.7-1	13	15	880	3,038	15.96			
h2a.w.6-2,7-1	1	4	239	845	15.56	15.29	0.87	WT<br>9.41×10^{-8}<br>*h2a.w.6*<br>0.141<br>*h2a.w.7*<br>0.965<br>*h2a.w-2*<br>1.000
h2a.w.6-2,7-1	2	6	451	1,580	15.41			
h2a.w.6-2,7-1	3	3	239	783	16.41			
h2a.w.6-2,7-1	4	14	686	2,355	16.35			
h2a.w.6-2,7-1	5	2	237	843	14.77			
h2a.w.6,7	6	8	419	1,640	14.24			
h2a.w.6-2,7-1	7	4	258	893	15.79			
h2a.w.6-2,7-1	8	1	152	512	15.43			
h2a.w.6-2,7-1	9	8	392	1,285	17.12			
h2a.w.6-2,7-1	10	5	281	960	16.20			
h2a.w.6-2,7-1	11	7	612	2,276	14.37			
h2a.w.6-2,7-1	12	6	611	2,309	14.01			
h2a.w.6-2,7-1	13	10	418	1,571	15.21			
h2a.w.6-2,7-1	14	13	772	2,911	14.60			
h2a.w.6-2,7-1	15	4	463	1,685	14.45			
h2a.w.6-2,7-1	16	8	758	2,668	15.10			
h2a.w.6-2,7-1	17	6	815	2,850	14.93			
h2a.w-2	1	3	303	1,032	15.55	15.24	0.86	WT<br>2.16×10^{-6}

h2a.w-2	2	3	256	857	15.99			*h2a.w.6*<br>0.132<br><br>*h2a.w.7*<br>0.937<br><br>*h2a.w.6,7*<br>1.000
h2a.w-2	3	4	392	1,386	15.01			
h2a.w-2	4	6	507	1,753	15.49			
h2a.w-2	5	9	390	1,580	14.05			
h2a.w-2	6	10	585	1,903	16.95			
h2a.w-2	7	8	565	2,138	14.34			
h2a.w-2	8	6	225	805	16.21			
h2a.w-2	9	8	498	1,739	15.70			
h2a.w-2	10	3	278	997	14.84			
h2a.w-2	11	11	748	2,793	14.57			
h2a.w-2	12	11	620	2,425	14.14			
h2a.w-2	13	16	621	2,347	15.27			

Table S10. Summary of next-generation sequencing libraries used in this study. The next-generation sequencing libraries used in this study are listed. All libraries were generated from plants in a Columbia-0 (Col) background, except for the GBS libraries, which were generated from Col-0 × L*er* F_2 plants.

Library	Dataset accession	Genotype	Run accession	Read length	Tissue	References
Input control	GSE95557	Col-0	SRR5298544	2×50 bp	Leaf	(11)
H2A.W.6 ChIP-seq		Col-0	SRR5298545	2×50 bp	Leaf	(11)
H2A.W.7 ChIP-seq		Col-0	SRR5298546	2×50 bp	Leaf	(11)
Input control	E-MTAB-7370	Col-0	ERR3813868	2×75 bp	Floral bud	(15)
H3K9me2 ChIP-seq		Col-0	ERR3813867	2×75 bp	Floral bud	(15)
Bisulfite-seq	E-MTAB-10657	Col-0	ERR6151793	2×100 bp	Floral bud	(6)
RNA-seq	E-MTAB-12699	Col-0	ERR10958076 - ERR10958078	2×100 bp	Seedling	(7)
RNA-seq	E-MTAB-12699	Col-0	ERR10958076 - ERR10958078	2×100 bp	Floral bud	(7)
RNA-seq	GSE86583	Col-0	SRR4204534-SRR4204536, SRR4204538-SRR4204340	1×50 bp	Meiocyte	(16)
gDNA	E-MTAB-6257	Col-0	ERR2215865	2×100 bp	Floral bud	(10)
MNase-seq	E-MTAB-14134 (< 2 mm bud)<br>E-MTAB-14673 (0.3–0.5 mm bud)<br>E-MTAB-14674 (seedling)	Col-0	ERR13179228-ERR13179230	2×100 bp	Floral bud	This study
MNase-seq		*h2a.w.6-3*	ERR13179237-ERR13179239	2×100 bp	Floral bud	This study
MNase-seq		*h2a.w.7-3*	ERR13179240-ERR13179242	2×100 bp	Floral bud	This study
MNase-seq		*h2a.w.6-3,7-3*	ERR13179234-ERR13179236	2×100 bp	Floral bud	This study
MNase-seq		*h2a.w-2*	ERR13179231-ERR13179233	2×100 bp	Floral bud	This study
GBS	E-MTAB-8165<br>E-MTAB-10168<br>E-MTAB-10657	WT<br>Col-0 × L*er*	ERR3482183-ERR3484102<br>ERR5385353-ERR5385496<br>ERR9361319-ERR9361414	2×150 bp	Leaf	(5–7, 14)
GBS	E-MTAB-14154	*mmH2A.W* × L*er*	ERR13230781-ERR13230972	2×150 bp	Leaf	This study
GBS	E-MTAB-14139	*mmSUVH* × L*er*	ERR13189179-ERR13189368	2×150 bp	Leaf	This study
GBS	E-MTAB-14153	*mmH2A.W mmSUVH* × L*er*	ERR13295589-ERR13295779	2×150 bp	Leaf	This study
GBS	E-MTAB-14136	*h2a.w.6-3* × *h2a.w.6-4*	ERR13185881-ERR13186071	2×150 bp	Leaf	This study
GBS	E-MTAB-14137	*h2a.w.7-3* × *h2a.w.7-4*	ERR13191376-ERR13191566	2×150 bp	Leaf	This study
GBS	E-MTAB-14146	*h2a.w.12-3* × *h2a.w.12-4*	ERR13295790-ERR13295981	2×150 bp	Leaf	This study
GBS	E-MTAB-14138	*h2a.w.6-3,7-3* × *h2a.w.6-4,7-4*	ERR13194666-ERR13194857	2×150 bp	Leaf	This study
GBS	E-MTAB-14161	*h2a.w-3* × *h2a.w.6-4*	ERR13244357-ERR13244643	2×150 bp	Leaf	This study

Table S11. Analysis of H2A.W ChIP-seq peaks. The H2A.W peaks used in this study are summarized. H2A.W.6 and H2A.W.7 ChIP-seq peaks were grouped into H2A.W.6-specific, H2A.W.7-specific, and their shared (H2A.W.6 ∩ H2A.W.7) peaks, and categorized into arm and pericentromeric regions. *n* indicates the number of ChIP-seq peaks corresponding to each group and region. The ratio between peaks in the arm and pericentromere of each group is shown.

Group	Region	*n*	Ratio (%)	Width (bp)	
				Mean	SD
H2A.W.6-specific	arm	150	18.84	262.62	118.62
	pericentromere	646	81.16	316.84	214.71
H2A.W.7-specific	arm	2,907	65.58	399.54	249.40
	pericentromere	1,526	34.42	381.49	243.47
H2A.W.6 ∩ H2A.W.7	arm	1,565	23.86	1,209.52	1,263.18
	pericentromere	4,994	76.14	3,019.20	5,001.54

Table S12. List of oligonucleotides used in this study.

Name	Nucleotide sequence (5′ to 3′)
<Cloning>	
*H2A.W.6-MIGS*_L0_F	TGTGGTCTCACCATGTGATTTTTCTCTACAAGCGAACACTCGTTTCCTGAAGAAAGG
H2A.W.6-MIGS _L0_R	TGTGGTCTCACATTCTTTCTTTGGAGACTTGACTGG
*H2A.W.7-MIGS*_L0_F	TGTGGTCTCAAATGGTGATTTTTCTCTACAAGCGAAATTTCCCGTTGGTCGTATCG
*H2A.W.7-MIGS*_L0_R	TGTGGTCTCAACCTTTAGGAGATTTGGTAGCAGAAG
*H2A.W.12-MIGS*_L0_F	TGTGGTCTCAAGGTGTGATTTTTCTCTACAAGCGAACCGGAACCAAAGTGAAGAAA
*H2A.W.12-MIGS*_L0_R	TGTGGTCTCAAAGCGCAACAGAACAAAACCCACA
*SUVH4-MIGS*_L0_F	CCGAAGACGGCTCACCATGTGATTTTTCTCTACAAGCGAACTGATTTGAAGGGTGTAA CTGA
*SUVH4-MIGS*_L0_R	CCGAAGACGGCTCGCATTTGACCACGAGTTACTCTGACAGGC
*SUVH5-MIGS*_L0_F	CCGAAGACGGCTCAAATGGTGATTTTTCTCTACAAGCGAAAGAGATGGTGGCAATTTC AAAGTGG
*SUVH5-MIGS*_L0_R	CCGAAGACGGCTCGACCTCCACCAGATACAATCCATCGTAAACATAATTC
*SUVH6-MIGS*_L0_F	CCGAAGACGGCTCAAGGTGTGATTTTTCTCTACAAGCGAAGCTTGTTGCGGGTAGAA AGAAGGC
*SUVH6-MIGS*_L0_R	CCGAAGACGGCTCGAAGCACTTGTCGCAACTTTGGCCTTACC
*CMT3-MIGS*_L0_F	CCGAAGACGGCTCAAATGGTGATTTTTCTCTACAAGCGAATTAAGGAGTGGGAGAAG CTATGCATTCATTTC
*CMT3-MIGS*_L0_R	CCGAAGACGGCTCGACCTCACTGATTCCTTGGCATGGTGGC
*CMT2-MIGS*_L0_F	CCGAAGACGGCTCAAGGTGTGATTTTTCTCTACAAGCGAATGCTCGGTTCCGGTGAC TGG
*CMT2-MIGS*_L0_R	CCGAAGACGGCTCGAAGCTGAGCAAGATGCCAGAGACATCCCTTTTCCTTTAGACTT GCC
*H2AW.6*_sgRNA1_F	ATTGTTCTTTTTTTTTCATTTACC
*H2AW.6*_sgRNA1_R	AAACGGTAAATGAAAAAAAAAGAA
*H2AW.6*_sgRNA2_F	ATTGCGCCGCAGAAGTAAGTGTTT
*H2AW.6*_sgRNA2_R	AAACAAACACTTACTTCTGCGGCG
*H2AW.7*_sgRNA1_F	ATTGCGTGTAATGTCATACAAAGA
*H2AW.7*_sgRNA1_R	AAACTCTTTGTATGACATTACACG
*H2AW.7*_sgRNA2_F	ATTGGTTGATTTAAACAATTTAGG
*H2AW.7*_sgRNA2_R	AAACCCTAAATTGTTTAAATCAAC
*H2AW.12*_sgRNA1_F	ATTGACTTCTTTCGCTATCTTACG
*H2AW.12*_sgRNA1_R	AAACCGTAAGATAGCGAAAGAAGT
*H2AW.12*_sgRNA2_F	ATTGTTTTGAGAATCTGAATACCT
*H2AW.12*_sgRNA2_R	AAACAGGTATTCAGATTCTCAAAA
<Genotyping>	
*hta6-2*_gt_F	AATTTCAGTAATCGATAACCGTAGC

*hta6-2*_gt_R	CTTCCCACTGGGAATTGAAGACCGGAT
*hta6-3*_gt_F (p417)	CACTCAGAAGTTTCCCCAATTCTTCATC
*hta6-3*_gt_R (p418)	CCAAAAACCCAAAATAAAGCAAGCGC
*hta7-1*_gt_F (p415)	GAATCGAACCAGAAAATTCATCATCGC
*hta7-1*_gt_R (p416)	GTCATGGAGCTTAGAAGATTACAGTTG
*hta7-3*_gt_F (p419)	GACACACAGATGGTGATTTCATATGGG
*hta7-3*_gt_R (p420)	CTTAGGGGATTTGGTTGCCTTTGATGG
*hta12-1*_gt_F	AGTTTCCTGTCGGTAGGATCG
*hta12-1*_gt_R	TCAGTTTTGTTTTTCCATCGG
*hta12-3*_gt_F	CTTCTCCTAAATTTTGGAGTATTGCTATTAATCC
*hta12-3*_gt_R	GCTCGAGAACCTAAATCGACAACATATATATAACG
*MIGS-H2AW*_gt_F	GTGATTTTTCTCTACAAGCGAACCG
*MIGS-H2AW*_gt_R	CACATGTGCATCCTCTAGTAGCG
*MIGS-SUVH*_gt_F	TTGAGGTTGGTGATGAGTTCC
*MIGS-SUVH*_gt_R	CACATGTGCATCCTCTAGTAGCG
*MIGS-CMT*_gt_F	TGCTCGGTTCCGGTGACTG
<RT-qPCR>	
*SUVH6*_RT_F	TTGAGGTTGGTGATGAGTTCC
*SUVH6*_RT_R	CGTTTCCGCCTTGACCAGTG
*SUVH5*_RT_F	GAACCCTGTGCGGGTCATAAG
*SUVH5*_RT_R	CTCTTTCCAGGGAAGCTCGG
*SUVH4*_RT_F	ACTGCTGCGAATATAATGTGCCTG
*SUVH4*_RT_R	TTCTGGTTGCCCCTCCAATC
*CMT2*_RT_F	GGTTCCGGTTACCAAAGGGG
*CMT2*_RT_R	AAATTTCCATCCACCGCCCT
*CMT3*_RT_F	TGTCCCCACTGTTGTCACAC
*CMT3*_RT_R	TTGGGTGGGCCAAAGAGTTT
*HTA6*_RT_F	GAGCTCTGGTGAATGTAGAAATTTG
*HTA6*_RT_R	GGACCAAAAGATTAGACGAAGCG
*HTA7*_RT_F	GTTTTAGAGGATTTGTGTAGGTATCT
*HTA7*_RT_R	AATCAACAAGTACGAAACCCAG
*HTA12*_RT_F	GCAGCTTCAACTACAAAAACACC
*HTA12*_RT_R	CTCACAACAACAAAGAGATATTCTG

Dataset S1 (separate file). Statistical analysis in Figure 1.

Dataset S2 (separate file). Statistical analysis in Figure 2.

Dataset S3 (separate file). Statistical analysis in Figure 3.

Dataset S4 (separate file). Sanger sequences for CRISPR/Cas9-mediated deletion alleles of *H2A.W.6*, *H2A.W.7*, and *H2A.W.12*.

Dataset S5 (separate file). Measurement of statistical analysis of crossover frequency over the *CEN3* genetic interval in WT and *h2a.w.6-3*, *h2a.w.7-3*, and *h2a.w.12-3* in *SI Appendix* Fig. 4*C.*

Dataset S6 (separate file). Statistical analysis in *SI Appendix* Figure S6.

Dataset S7 (separate file). Statistical analysis in Figure 5 *A* and *B*.

Dataset S8 (separate file). Images of immunoblot replicates in Figure 5*C.*

SI References

1. A. De Muyt, *et al.*, E3 ligase Hei10: A multifaceted structure-based signaling molecule with roles within and beyond meiosis. *Genes Dev* **28**, 1111–1123 (2014).
2. K. E. Francis, *et al.*, Pollen tetrad-based visual assay for meiotic recombination in Arabidopsis. *Proc Natl Acad Sci U S A* **104**, 3913–8 (2007).
3. E. C. Lim, *et al.*, DeepTetrad: high-throughput image analysis of meiotic tetrads by deep learning in Arabidopsis thaliana. *Plant Journal* **101**, 473–483 (2020).
4. P. Bourguet, *et al.*, The histone variant H2A.W and linker histone H1 co-regulate heterochromatin accessibility and DNA methylation. *Nat Commun* **12**, 1–12 (2021).
5. D. C. Nageswaran, *et al.*, HIGH CROSSOVER RATE1 encodes PROTEIN PHOSPHATASE X1 and restricts meiotic crossovers in Arabidopsis. *Nat Plants* **7**, 452–467 (2021).
6. J. Kim, *et al.*, Arabidopsis HEAT SHOCK FACTOR BINDING PROTEIN is required to limit meiotic crossovers and HEI10 transcription. *EMBO J* **41**, e109958 (2022).
7. H. Kim, *et al.*, Control of meiotic crossover interference by a proteolytic chaperone network. *Nat Plants* **10**, 453–468 (2024).
8. C. Melamed-Bessudo, E. Yehuda, A. R. Stuitje, A. A. Levy, A new seed-based assay for meiotic recombination in Arabidopsis thaliana. *Plant J* **43**, 458–66 (2005).
9. B. A. Rowan, V. Patel, D. Weigel, K. Schneeberger, Rapid and inexpensive whole-genome genotyping-by-sequencing for crossover

localization and fine-scale genetic mapping. *G3: Genes, Genomes, Genetics* 5, 385–398 (2015).
10. K. Choi, *et al.*, Nucleosomes and DNA methylation shape meiotic DSB frequency in Arabidopsis thaliana transposons and gene regulatory regions. *Genome Res* 28, 532–546 (2018).
11. Z. J. Lorković, *et al.*, Compartmentalization of DNA Damage Response between Heterochromatin and Euchromatin Is Mediated by Distinct H2A Histone Variants. *Current Biology* 27, 1192–1199 (2017).
12. S. J. Armstrong, A. P. Caryl, G. H. Jones, F. C. H. Franklin, Asy1, a protein required for meiotic chromosome synapsis, localizes to axis-associated chromatin in Arabidopsis and Brassica. *J Cell Sci* 115, 3645–3655 (2002).
13. J. Walker, *et al.*, Sexual-lineage-specific DNA methylation regulates meiosis in Arabidopsis. *Nat Genet* 50, 130–137 (2018).
14. B. A. Rowan, *et al.*, An Ultra High-Density Arabidopsis thaliana Crossover Map That Refines the Influences of Structural Variation and Epigenetic Features. *Genetics* 213, 771–787 (2019).
15. C. Lambing, *et al.*, Interacting genomic landscapes of REC8-cohesin, chromatin and meiotic recombination in Arabidopsis thaliana. *Plant Cell* 32, tpc.00866.2019 (2020).
16. J. Walker, *et al.*, Sexual-lineage-specific DNA methylation regulates meiosis in Arabidopsis. *Nat Genet* 50, 130–137 (2018).
