# Supplementary material for: The histone variant H2A.W restricts heterochromatic crossovers in Arabidopsis
Source: Proc Natl Acad Sci U S A. 2025 Apr 4;122(14):e2413698122. doi: 10.1073/pnas.2413698122 (PMC12002335; doi:10.1073/pnas.2413698122)
Supplement: Supplementary file 5 — Dataset S04 (PDF) [file pnas.2413698122.sd04.pdf]

Sequences for CRISPR-mediated deletion alleles of *H2A.W* paralogs.

Annotation codes:

5' UTR
exon
intron
3'UTR
sgRNA target sequence
Sequence deleted in mutants
Sequence inverted in mutants

H2A.W.6 (AT5G59870; Col-0)
deletion allele = *h2a.w.6-3*

catacctctttttgattggttaatacatagtcacgcggatcgtgctttatttgaacatccaccgtcgatagactaaatccaacggataataatcctctccc***ttctttttttttttcatttacc***tataaatatcacagagtacccttcaactttaaatcacaaatcttcaacttccgatactttcaatctctctaaactctcaatttcagtaatcgataaccgtagcaATGGAATCCACCGGAAAAGTGAAGAAAGCTTTCGGAGGAAGAAAACCACCTGGTGCCCCAAAAACCAAATCGGTTTCGAAATCGATGAAAGCCGGTCTTCAATTCCCAGTGGGAAGAATCACTCGTTTCCTGAAGAAAGGACGATACGCTCAGAGACTTGGTGGTGGTGCTCCGGTTTACATGGCCGCCGTTCTTGAATACCT***CGCCGCAGAAgtaagtgttt***cccgatctggattttctagtaagattttttttttacatttcaaaatcaattttctgattcgaatttattgatctcagGTTCTGGAGCTTGCTGGTAACGCTGCGAGAGATAACAAGAAATCAAGGATAATTCCGAGGCATCTTCTTCTCGCGATAAGGAACGATGAAGAATTGGGGAAACTTCTGAGTGGTGTCACAATCGCTCACGGTGGTGTTTTGCCTAACATCAACTCTGTTCTATTGCCTAAGAAGTCTGCCACTAAACCAGCTGAAGAAAAGGCTACCAAATCACCAGTCAAGTCTCCAAAGAAAGCTTAAtctgctagagttttcgttgctagtttgtgtttgagctctggtgaatgtagaaatttgaagcttttggatcttagtttctatgtatttggtgatttagaatgttgttcaaaatccttttcctaatcataagaatttatgatctatctattatacgcttcgtctaatcttttggtccactcgtcgtaatgtcattagtgaatatttaataaacaactttgtcatcgacattaacgaacccttttattcgctgtgctaaatttttcttttaggtgaagccaaatctaa

H2A.W.6 (AT5G59870; Ler-0)
deletion allele = *h2a.w.6-4*

gtcgatagaataaatccaacggataataatcctctccc***ttctttttttttttcatttacc***tataaatatcacagagtacccttcaactgtaaatcacaaatcttcaacttccgatactttcaatctctctaaactctcaatttcagtaatcgataaccgtagcaATGGAATCCACCGGAAAAGTGAAGAAAGCTTTCGGAGGAAGAAAACCACCTGGTGCCCCAAAAACCAAATCGGTTTCGAAATCGATGAAAGCCGGTCTTCAATTCCCGGTGGGAAGAATCACTCGTTTCCTGAAGAAAGGACGATACGCTCAGAGACTTGGTGGTGGTGCTCCGGTTTACATGGCCGCCGTTCTTGAATACCT***CGCCGCAGAAgtaagtgttt***cccgatctggattttctagtaagatttttttttttcatttcaaaatcaattttctgattcgaatttattgatctcagGTTCTGGAGCTTGCTGGTAACGCTGCGAGAGATAACAAGAAATCAAGGATAATTCCGAGGCATCTTCTTCTCGCGATAAGGAACGATGAAGAATTGGGGAAACTTCTGAGTGGTGTCACAATCGCTCACGGTGGTGTTTTGCCTAACATCAACTCTGTTCTATTGCCTAAGAAGTCTGCCACTAAACCAGCTGAAGAAAAGGCTACCAAATCACCAGTCAAGTCTCCAAAGAAAGCTTAAtctgctagagttttcgttgctagtttgtgtttgagctctggtgaatgtagaaatttgaagcttttggatcttagtttctatgtatttggtgatttagaatgttgttcaaaatccttttcctaatcataagactttatgatctatctattatacgcttcgtctaatcttttggtccactcgtcgtaatgtcattagtgaatatttaataaacaactttgtcatcgacattaacgaacccttttattcgctgtgctaaatttttcttttaggtgaagc

H2A.W.7 **(*AT5G27670*; Col-0)**
deletion allele = *h2a.w.7-3*

gtggggaatgaatctaacggctgatatcactcaagtgttcttttccaccttccttttacaacaccca***cgtgtaatgtcatacaaaga***agtcattac
gaccgttagatcaaagccaacaagatccaatcttaacggctaagataaattactacacggatcgccaacgtggcaatacgtggtatatacata
cacgtcgttctttcctcattttaagcaaatcgtaaaccgccacaaaaccgaaaaaaacactaattgtgctttccctttagattcatttgtattttcttttgg
agcttttgaacaATGGAGTCATCACAAGCAACGACGAAGCCAACGAGAGGAGCAGGAGGAAGGAAAG
GTGGAGATAGGAAGAAGAGTGTTAGTAAATCTGTTAAAGCTGGTCTTCAATTTCCCGTTGGTCGTA
TCGCTCGTTACTTGAAGAAAGGTCGGTACGCTCTCCGATACGGTTCCGGTGCTCCGGTTTACCTC
GCCGCCGTTCTCGAATACCTAGCCGCCGAGgtatattcaatctcagatctcgttgcattttgaatcgatttattttgtgtatctatta
gatctgtttaattttgaagttctaatgaattgaaccggtttggtttagGTACTTGAGCTAGCTGGGAACGCAGCGAGAGATAA
TAAGAAGAACAGGATAAACCCTAGGCATCTATGTTTAGCGATAAGGAACGATGAGGAATTGGGGAG
ATTGCTTCATGGAGTTACTATTGCTAGTGGTGGTGTTCTTCCAAACATTAATCCAGTTCTTCTTCCT
AAGAAATCAACAGCTTCTTCTTCTCAAGCGGAGAAAGCTTCTGCTACCAAATCTCCTAAGAAGGCT
TGAtaaagaatagtatcgatgttgctttttggttatattcggatcttagatgaagaagaagaagaagaagaaacaacttgttttttgttttagaggat
ttgtgtaggtatctgaaatcttcttctctttgttttggtttgtcttatgtaaaaaccatgggaagatgattatgtttgttaacgcaatttgtaatggaaaataa
ttaagttctgggattagtaacttcatctgtctaattaatttctgggtttcgtactt***gttgatttaaacaatttagg***tggattaattgaaatggttttggtata
cacatggaaagattcagtacagttaatgacattaattaaagtagataataatcacgaaaaacatgacattaattaagaaaatgattg

H2A.W.7 **(*AT5G27670*; L*er*-0)**
deletion allele = *h2a.w.7-4*

aaaaagacataaagctgattatcttgtagaacgtgtggggaatgaatctaacggctgatatcactcaagtgttcttttccaccttccttttacaacac
cca***cgtgtaatgtcatacaaaga***agtcattacaaccgttagatcaaagccaacaagatccaatcttaacggctaagataaattactacacgg
atcgccaacgtggcaatacgtggtatatacatacacgtcgttccttcctcattttacgcaaatcgtaaaccgccacaaaaccgaaaaaaacact
aattgtgctttccctttagattcatttgtatttacttttggagcttttgaacaATGGAGTCATCACAAGCAACGACGAAGCCAACG
AGAGGAGCAGGAGGAAGGAAAGGTGGAGATAGGAAGAAGAGTGTTAGTAAATCTGTTAAAGCTG
GTCTTCAATTTCCCGTTGGTCGTATCGCTCGTTACTTGAAGAAAGGTCGTTACGCTCTCCGATACG
GTTCCGGTGCTCCGGTTTCCCTCGCCGCCGTTCTCGAATACCTAGCCGCCGAGgtaaattcaacttcaga
tctcgttgcattttgaatcggtttattttgtgtatctattagatctgtttaattttggagatctaacgaattgaaccgggttggtttagGTACTTGAGC
TAGCTGGGAACGCAGCGAGAGATAATAAGAAGAACAGGATAAACCCTAGGCATCTATGTTTAGCGA
TAAGGAACGATGAAGAATTGGGGAGATTGCTTCATGGAGTTACTATTGCAAGTGGTGGTGTTCTTC
CTAACATTAATCCAGTTCTTCTTCCTAAGAAATCAACAGCTTCTTCTTCTCAAGCTGAGAAAGCTTC
TGCTACCAAATCTCCTAAGAAGGCTTGAtaaagaatagtatcgatgttgctttttggttatattcggatcttagatgaagaagaa
gaagaagaagaaacaacttgtttttttttttagaggatttgtgtaggtatctgaaatcttcttctctttgttttggtttgtcttatgtaaaaaccatgggaag
atgattatgtttcttacgcaatttgtaatggaaaataagtaatttctgggattagtaacatcatctgtctaattaatttctgggtttcgtactt***gttgatttaa***
acaatttaggtggattaattgaaatggttttggtatacatagaaagagtcagcaccattaattaaagtagataataatcacgaaaaacatgacat
taattaagaaaatgattgttgggcttagatgataggcccattaaaatttatgtgcttggttcatctacgagattctggaa

***H2A.W.12* (*AT5G02560*; Col-0)**
deletion allele = *h2a.w.12-3*

cacttccaatccaaaagctaacattcatcaactgacaaaaccaaccaaccaacca***<u>acttctttcgctatcttacg</u>***ccaaagctctcttaattcc
tccgtttgcatattttccggtcagatcaaaatcagaatcagaatcaaatttctcgtcgtgtcggagtaaatcaagccATGGATTCCGGAAC
CAAAGTGAAGAAAGGAGCCGCTGGAAGAAGAAGTGGTGGAGGTCCTAAGAAGAAACCGGTTTCC
CGTTCGGTTAAATCCGGTCTACAGTTTCCTGTCGGTAGGATCGGTCGGTATCTTAAGAAAGGTCGT
TATTCGAAGCGTGTCGGAACCGGAGCTCCGGTCTATCTCGCCGCCGTCCTCGAGTATCTTGCTGC
TGAGgtaataaagttctgaattcagatcagctaatcatttcatcggaattatcgcagtttcatcgatttcactagAATTCTTGTGGGTTT
TGTTCTGTTGCTTCGTTGACCATCTATAGGTGTAGAATGTCTTCTTCTGATTTTAGGgtaaattgataatcat
ctgaggttgtaaaattgaatttgttagatactatatcacgagtagatcaacctcaagacatggtttcactttcaattaggtttaacatctttgctttgcaa
atctcaaaatcttagatagagatatattagcgttacataaaaactaaagttgcatagtcaataaaacctaaataaaacatctgcaagtaaacttc
attgagaatctatcatcatgtaacaccg***<u>ttttgagaatctgaatacct</u>***tggactgatgtgcatgttacatgtaactcttgtcaacaaatctctgagta
actaggatatgcaaatattgcatactaatctttttgatcgaatgtgacaaaaccccattttaaagtttacaagtctgatccgttatatatatgttgtcgat
ttagGTTCTCGAGCTTGCTGGTAACGCTGCAAGAGATAACAAAAAGAACCGTATTATACCACGCCAT
GTTCTATTAGCGGTGAGGAACGACGAGGAGCTAGGGACACTACTCAAAGGCGTAACCATTGCACA
CGGCGGTGTTTTACCAAACATAAACCCAATACTCCTCCCAAAGAAGTCTGAGAAAGCAGCTTCAA
CTACAAAAACACCCAAATCACCATCAAAGGCAACCAAATCCCCTAAGAAATCTTAGtacttctttcttcattcct
ctgtataacctactgtttctatctctctgtacgtttctctgtaaagacagaacagaatatctctttgttgttgtgagaaagcttagtttctctgatcgtcgttg
tgaaataaaaaatgcaacgtttcatatagattttgcacaatcaaaaagtattcatataaacaatgtattattattcgactatcat

***H2A.W.12* (*AT5G02560*; L*er*-0)**
deletion allele = *h2a.w.12-4*

gttgattcactttcaatccaaaagctaacattcatcaactgacaaaaccaaccaaccaacca***<u>acttctttcgctatcttacg</u>***ccaaagctttctt
aattcctccgtttgcatattttccggtcagatcaaaatcagaatcagaatcaaatttctcgtcgtgtcggagtaaatcaagccATGGATTCCG
GAACCAAAGTGAAGAAAGGAGCCGCTGGAAGAAGAAGTGGTGGAGGTCCTAAGAAGAAACCGG
TTTCCCGTTCGGTTAAATCCGGTCTACAGTTTCCTGTCGGTAGGATCGGTCGGTATCTTAAGAAAG
GTCGTTATTCGAAGCGTGTCGGAACCGGAGCTCCGGTCTATCTCGCCGCCGTCCTCGAGTATCTT
GCTGCTGAGgtaataaagttctgaattcagatcagctaatcatttcatcggaattatcgcagtttcatcgatttcactagAATTCTTGTG
GGTTTTGTTCTGTTGCTTCGTTGACCATCTATAGGTGTAGAATGTCTTCTTCTGATTTTAGGgtaaattg
ataatcatctgaggttgtaaaattgaatttgttagatactatatcacgagtagatcaacctcaagacatggtttcactttcaattaggtttaacatctttg
ctttgcaaatctcacaatcttagatagagatatattagcgttacataaaaactaaagttgcatagtcaataaaacctaaataaaacatctgcaggt
aaacttcattgagaatctatcatcatgtaacaccg***<u>ttttgagaatctgaatacct</u>***tggactgatgtgcatgttacatgtaactcttgtcaacaaatct
ctgagtaactaggatatgcaaatattgcatactaatctttttgatcgaatgtgacaaaaccccattttaaagtttacaagtctgatcctttatatatatgt
tgtcgatttagGTTCTCGAGCTTGCTGGTAACGCTGCAAGAGATAACAAAAAGAACCGTATTATACCACG
CCATGTTCTATTAGCGGTGAGGAACGACGAGGAGCTAGGGACACTACTCAAAGGCGTAACCATTG
CACACGGCGGTGTTTTACCAAACATAAACCCAATACTCCTCCCAAAGAAGTCTGAGAAAGCAGCT
TCAACTACAAAAACACCCAAATCACCATCAAAGGCAACCAAATCCCCTAAGAAATCTTAGttcttctttcttc
attcctctgtataacctactgtttctatctctctgtacgtttctctgtaaagacagaacagaatatctctttgttgttgtgagaaagcttagtttctctgatcg
tcgttgtgaaataaaaaatgcaacgtttcatatagattgtgcac
