# Supplementary material for: The histone variant H2A.W restricts heterochromatic crossovers in Arabidopsis
Source: Proc Natl Acad Sci U S A. 2025 Apr 4;122(14):e2413698122. doi: 10.1073/pnas.2413698122 (PMC12002335; doi:10.1073/pnas.2413698122)

Dataset S8

Original western blot membrane images for Figure 5C, D are presented. Dashed rectangles in the full membrane images mark the specific regions shown in Figure 5C.

meiocyte

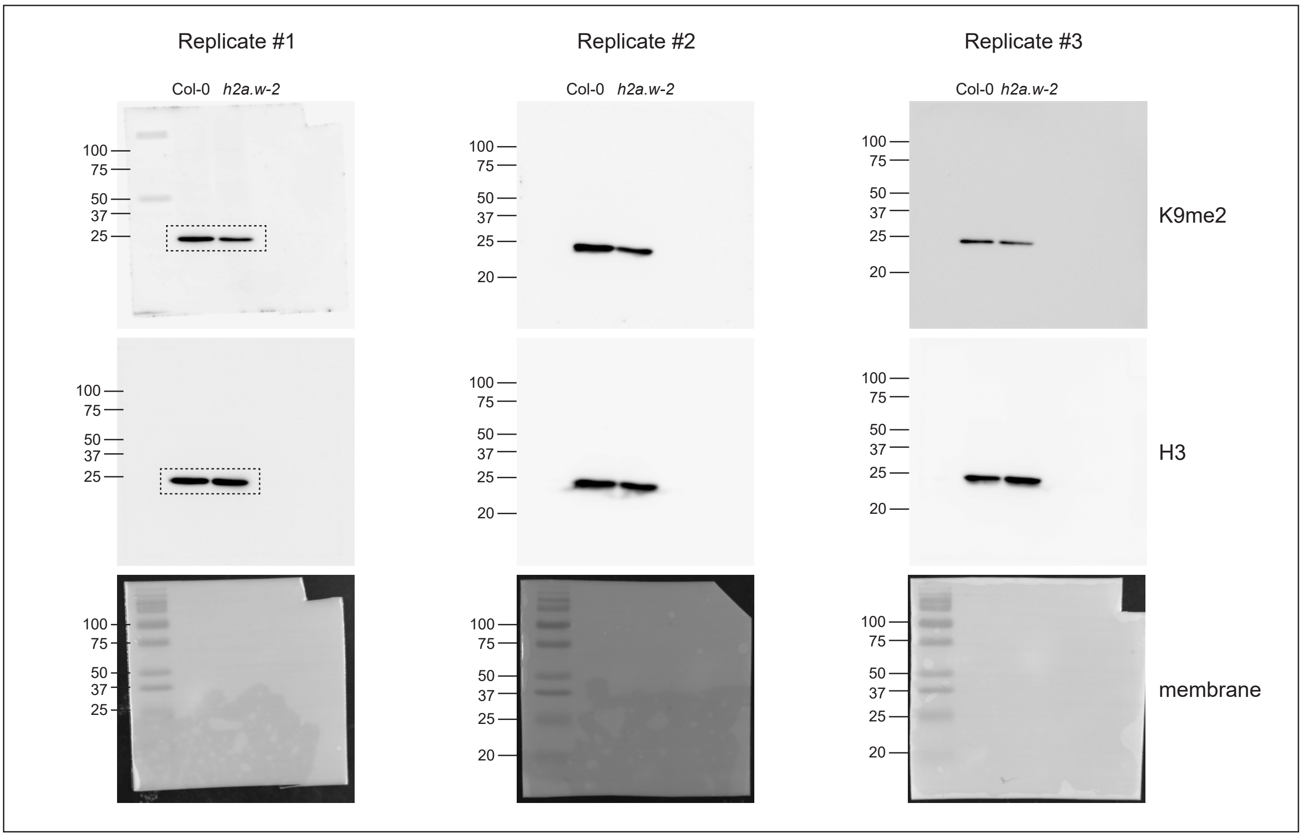

seedling

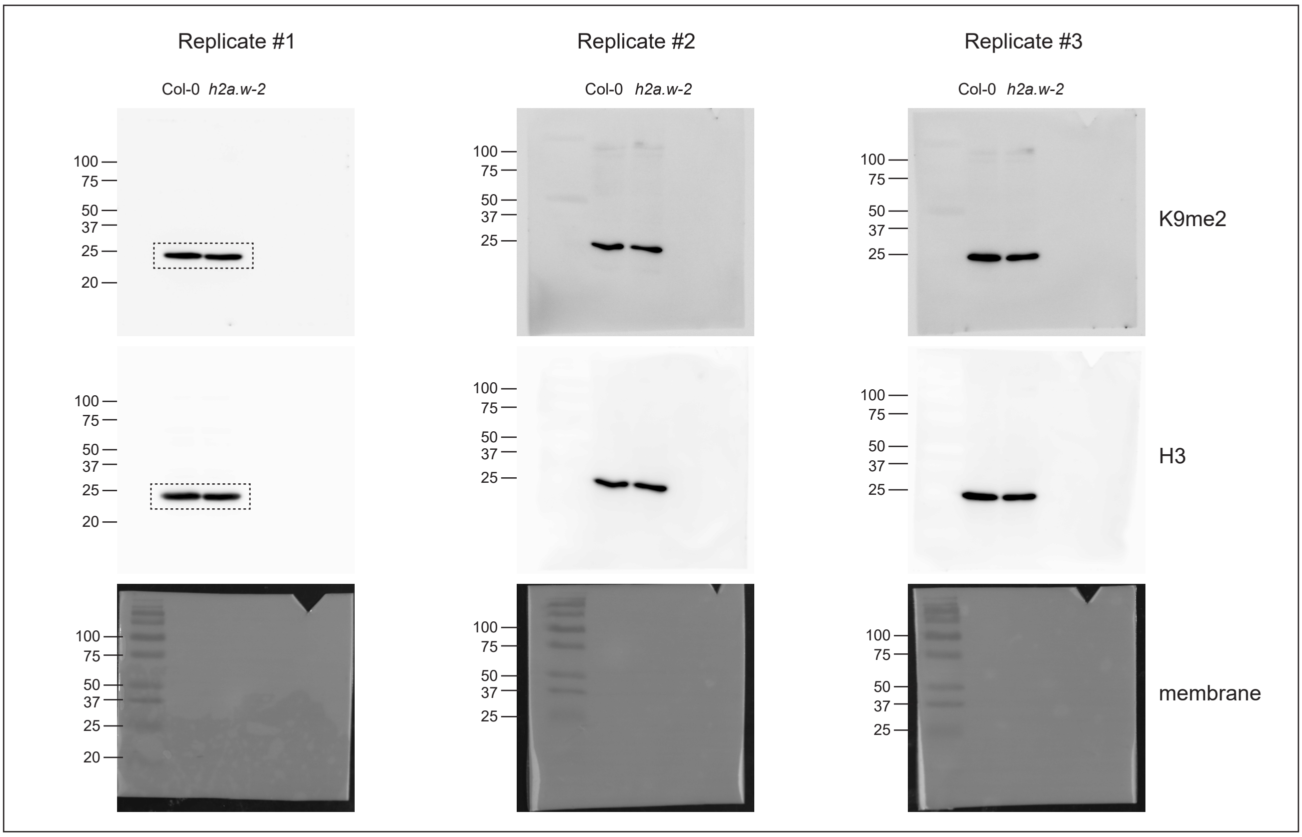

Supplement: Supplementary file 9 — Dataset S08 (PDF) [file pnas.2413698122.sd08.pdf]
